# Catalytic Activity and Electrochemical Stability of Ru_1–*x*_M_*x*_O_2_ (M = Zr, Nb, Ta): Computational and Experimental Study of the Oxygen Evolution Reaction

**DOI:** 10.1021/acsami.4c01408

**Published:** 2024-03-19

**Authors:** Francisco Ospina-Acevedo, Luis A. Albiter, Kathleen O. Bailey, José Fernando Godínez-Salomón, Christopher P. Rhodes, Perla B. Balbuena

**Affiliations:** † Department of Chemical Engineering, Texas A&M University, College Station, Texas 77843, United States; ‡ Materials Science, Engineering and Commercialization Program, Texas State University, San Marcos, Texas 78666, United States; § Department of Chemistry and Biochemistry, Texas State University, San Marcos, Texas 78666, United States

**Keywords:** oxygen evolution reaction, electrocatalysis, ruthenium oxide, metal substitution, proton exchange membrane water electrolysis

## Abstract

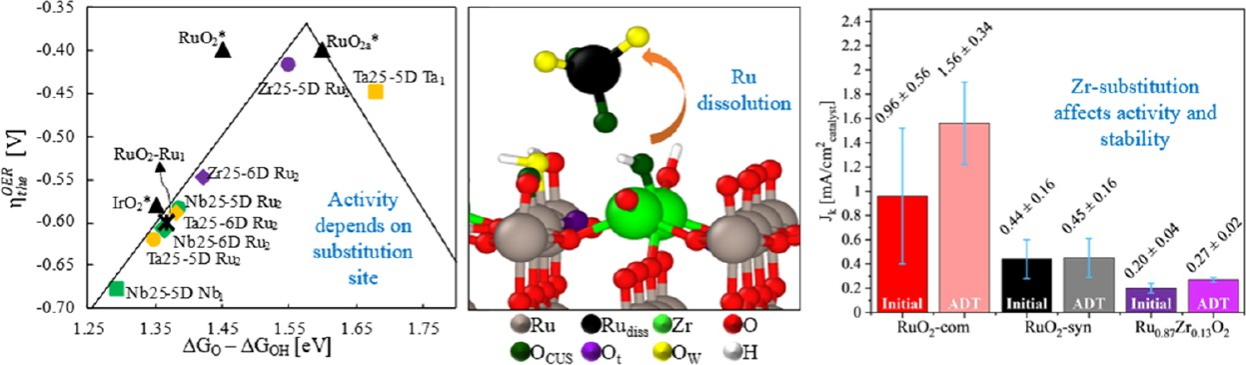

We use computations and experiments to determine the effect of substituting zirconium, niobium, and tantalum within rutile RuO_2_ on the structure, oxygen evolution reaction (OER) mechanism and activity, and electrochemical stability. Calculated electronic structures altered by Zr, Nb, and Ta show surface regions of electron density depletion and accumulation, along with anisotropic lattice parameter shifts dependent on the substitution site, substituent, and concentration. Consistent with theory, X-ray photoelectron spectroscopy experiments show shifts in binding energies of O-2s, O-2p, and Ru-4d peaks due to the substituents. Experimentally, the substituted materials showed the presence of two phases with a majority phase that contains the metal substituent within the rutile phase and a second, smaller-percentage RuO_2_ phase. Our experimental analysis of OER activity shows Zr, Nb, and Ta substituents at 12.5 atom % induce lower activity relative to RuO_2_, which agrees with computing the average of all sites; however, Zr and Ta substitution at specific sites yields higher theoretical OER activity than RuO_2_, with Zr substitution suggesting an alternative OER mechanism. Metal dissolution predictions show the involvement of cooperative interactions among multiple surface sites and the electrolyte. Zr substitution at specific sites increases activation barriers for Ru dissolution, however, with Zr surface dissolution rates comparable to those of Ru. Experimental OER stability analysis shows lower Ru dissolution from synthesized RuO_2_ and Zr-substituted RuO_2_ compared to commercial RuO_2_ and comparable amounts of Zr and Ru dissolved from Zr-substituted RuO_2_, aligned with our calculations.

## 1 Introduction

Hydrogen produced from water electrolysis in a proton exchange membrane (PEM) electrolyzer is one of the most promising processes to store renewable energy in the form of hydrogen fuel^1,2^ and can be powered by clean, renewable sources including wind and solar energy.^3^ Electrochemical water splitting (overall reaction: 2H_2_O → O_2_ + 2H_2_) in acid occurs via the anodic oxygen evolution reaction (OER: 2H_2_O → O_2_ + 4 H^+^ + 4 e^–^) and the cathodic hydrogen evolution reaction (HER: 2H^+^ + 2e^–^ → H_2_). However, the efficiency of PEM electrolyzers is hindered by the sluggish OER reaction kinetics resulting in high overpotentials and significant efficiency losses.^4−6^ In contrast to the slow kinetics of the OER, the HER involves fast kinetics and allows high voltage efficiencies at high current densities and high pressures.^4,7^ Research efforts, for both acidic and alkaline applications,^8−13^ focus on the development of more active, more stable, and lower-cost OER electrocatalysts^14^ by synthesizing new materials to modify their composition and structure that reduces material losses while enhancing the OER kinetics.

Noble-metal-based catalysts and their oxides^15−20^ are usually the top candidates for acidic electrochemical catalysis. However, prior work indicates that acidic OER catalysts with reasonable activity are very unstable and dissolve under highly oxidative potentials (≥1.5 V_RHE_) and strongly acidic environment (pH ≤ 1) of PEM electrolyzers.^3,21,22^ Thus, one of the main challenges for the wide-scale adoption of PEM electrolyzers is to obtain active, stable, and low-cost OER electrocatalysts.^3,23,24^ Currently, iridium-based catalysts are considered to exhibit the best balance of activity and stability^25^ and consequently have been frequently the focus of both experimental and theoretical research efforts.^4,5,7,14,22,26,27^ Iridium-based materials have high costs driven by their limited global supply,^21^ which is a key issue affecting their utilization, and the development of non-Ir catalysts is of great interest.^24^

Ruthenium in both oxide (RuO_*x*_)^28,29^ and metallic^22,29^ forms has shown appreciably higher acidic OER activity compared to Ir and IrO_*x*_, requiring lower-energy input due to their lower overpotentials. In addition, Ru has a higher global supply^30^ and hence lower cost^31^ compared to Ir, and Ru is an excellent candidate for large-scale catalytic applications. However, Ru-based catalysts show a high degree of instability at operating conditions,^22,32^ resulting in significant metal dissolution into the electrolyte that leads to higher and faster catalyst degradation.^4^ Metal substitution and interaction with support materials have been widely investigated as an alternative to improve both activity and stability,^33−41^ and recent work on metal-substituted Ir and Ru oxides as OER catalysts^41−43^ has highlighted the main challenges of the OER on Ru and Ir-oxide-based catalysts. The simultaneous onset potential of OER and Ru or Ir dissolution is a significant issue because of the impact of the dissolution of active sites and ultimately on the catalytic activity. The OER catalytic mechanism has also been discussed and debated extensively in numerous studies.^41−43^ Two main mechanisms are usually cited: the adsorbate evolution mechanism (AEM) and the lattice oxygen participation mechanism (LOM), and because the various characterization experiments measure the catalytic activity on average surface structures, the exact influence of one or the other mechanism is difficult to assess. Thus, at this moment, our best option is to combine the analyses of first-principles simulations on well-defined surfaces with multimodal experimental interrogation. Following this strategy, our previous density functional theory (DFT) calculations showed that doping of Co within the RuO_2_ surface influences the electron density and alters the adsorption energy of intermediate species, resulting in structures with lower activation energies.^39^ Our recent study on Ti substitution within both the bulk structure and surface of RuO_2_ also showed specific effects on the electron density of the structures, highly dependent on dopant location, and affecting both the Ru d band and O-2p band centers toward higher binding energies, in agreement with experimental X-ray photoelectron spectroscopy (XPS) analyses.^44^ We have also shown that specific substituents can act as components that are able to mitigate the dissolution process. This is because some of these elements can offer a sacrificial dissolution of specific sites based on electronic effects that may induce surface rearrangements. Theoretical analyses of Ti-substituted Ru oxide surfaces showed that the lowest barriers for catalytic OER exist at penta-coordinated Ru sites, while hexa-coordinated Ru sites have the lowest energetic barriers for dissolution.^44^ Thus, the substitution of Ru by other elements with different oxidation potentials may contribute to the stabilization of the Ru active sites.^45−55^

In addition to titanium, understanding the effects of substitution of other metals within RuO_2_ affects the structure, OER activity, and mechanism, and stability is of interest. Zirconium, niobium, and tantalum are particularly interesting substituents based on their ability to form passivating surface layers that prevent further corrosion in acid and their domains of passivity from Pourbaix diagrams.^56,57^ Goodenough and Manoharan investigated the electrochemical properties of RuZr metal alloys in acid and showed that the presence of Zr shifts the Ru corrosion reaction to more positive voltages, which was attributed to the presence of an anodically formed film containing more basic Zr–O–Ru bonds.^58^ Mixtures of RuO_2_–ZrO_2_ thin films supported on Ti have been investigated as dimensionally stable anodes (DSAs) for chlor-alkali production.^59,60^ However, these studies^59,60^ and others^61−64^ have not been able to produce a solid solution or single-phase Ru_1–*x*_Zr_*x*_O_2_ material. Obtaining a solid solution phase containing the metal substituent is advantageous to modify the electrochemical properties of the catalyst system.^65^ A bimetallic catalyst system consisting of Nb_2_O_5_ and RuO_2_ (Ru_*x*_Nb_1–*x*_O_2_) showed that the OER activity decreased as the concentration of Nb_2_O_5_ increased, and at 20% Nb_2_O_5_ addition, the stability was higher than that of RuO_2_.^40^ In another study, Nb_0.1_Ru_0.9_O_2_ exhibited high OER activity and a low degradation rate when compared to RuO_2_, and increasing Nb doping above 20% decreased the OER activity.^66^ A prior study of RuO_2_–Ta_2_O_5_ showed the addition of up to 10% of Ta_2_O_5_ to RuO_2_ resulted in a solid solution (phase separation occurred beyond 10 atom % of Ta), and the 90%RuO_2_-10%Ta_2_O_5_ catalyst was found to be more active and stable when compared to RuO_2_ when tested at high temperatures (150 °C).^67^

The studies mentioned above involving various compositions of Ru and Nb, Ta, or Zr have shown improvements in the performance of these materials in terms of OER stability, OER activity, and corrosion resistance. However, there is still room for development of a single-phase metal-substituted OER catalyst with high activity and stability and to understand the effect of dopant species within the OER mechanism and catalyst activity, dissolution, and electronic structure. In addition, due to the important advances in computational chemistry and surface science techniques, combined evaluations using both simulation and experimental analysis are becoming essential to accelerate the discovery, development, and design of materials with desired properties to improve the efficiency of electrocatalyst materials.^14^ In this work, we combine DFT calculations with experimental analysis to evaluate the effect of substituents M on the atomic and electronic structure, OER activity, and catalyst stability of substituted Ru_1–*x*_M_*x*_O_2_ (M = Zr, Nb, Ta) at different M atomic concentrations. The analysis and comparison of both theoretical and experimental findings bring new insights into the effect of doping on catalytic materials and help the development of new materials for more active and stable OER electrocatalysts.^14^

## 2 Methods

### 2.1 Computational Methods

All calculations were performed using the density functional theory (DFT) method as implemented in the Vienna Ab Initio Simulation Package (VASP—version 5.4.4),^68−70^ with the plane-wave basis set including spin polarization to describe the valence electrons using a cutoff energy of 400 eV, based on the maximum energy cutoff for the plane-wave basis set for each species present. In our models, the metals (Ru, Zr, Nb, and Ta) exhibit maximum energy cutoffs of 240, 229, 293, and 223 eV, respectively, while O and H exhibit 400 and 250 eV, respectively. Hence, it is expected that the presence of oxygen species rules the energy cutoff in our systems, being 400 eV large enough for the description of the plane-wave basis for the metals included in our calculations. The treatment of the core-electron interactions was done with the recommended projector augmented wave pseudopotentials (PAW),^71,72^ i.e., Ru_pv, Zr_sv, Nb_sv, Ta_pv, O, and H. We used the revised Perdew–Burke–Ernzerhof generalized gradient approximation (revPBE-GGA)^73,74^ for the exchange–correlation functional since its use has shown improvement in the energetics of adsorption phenomena on transition-metal surfaces.^75^ The integration of Brillouin zone was done with the γ centered Monkhorst–Pack grid method,^76^ and the Gaussian smearing method with an energy smearing set to 0.02 eV allowed for determining the electronic occupancies in all calculations. The energies and forces convergence criteria were set to 10^–5^ eV and 0.02 eV/Å, respectively, while long-range van der Waals interactions were incorporated using the Grimme’s D3-dispersion correction.^77,78^ Inclusion of the Hubbard *U* correction as proposed by Dudarev et al.^79^ to the Zr, Nb, and Ta revPBE-GGA functionals is done. Due to the variable nature of the *U* correction, several methods have been proposed to calculate an accurate *U* value.^80,81^ We adopted the use of the linear response ansatz as proposed by Cococcioni et al. The values of the *U* correction for Zr, Nb, and Ta used in this work are 3.2, 1.1, and 3.5, respectively.

The RuO_2_ unit cell used to model the Ru_1–*x*_M_*x*_O_2_ bulk systems and their facets was taken from our previous report,^44^ whose lattice parameters are in excellent agreement with experimentally reported values.^82−85^ After optimization of the RuO_2_ bulk structure, a 2 × 2 supercell was made to evaluate the effect of various M substituents (M = Zr, Nb, Ta) at bulk concentrations of 12.5, 25, and 50%, and theoretical X-ray diffraction patterns were obtained using the software VESTA^86,87^ for the calculated structures of each bulk system. For the surface calculations, a periodic three-layer, 2 × 1 (110)-RuO_2_ slab with a surface area of 6.22 × 6.34 Å^2^ and 10 Å of vacuum space on top of the surface was used to avoid any periodic interactions. We used surface atomic concentrations of 25 and 50% as previously reported in our group^14,26,39^ to compare with experimental surface concentrations, as discussed in the text.

**Figure 1:**
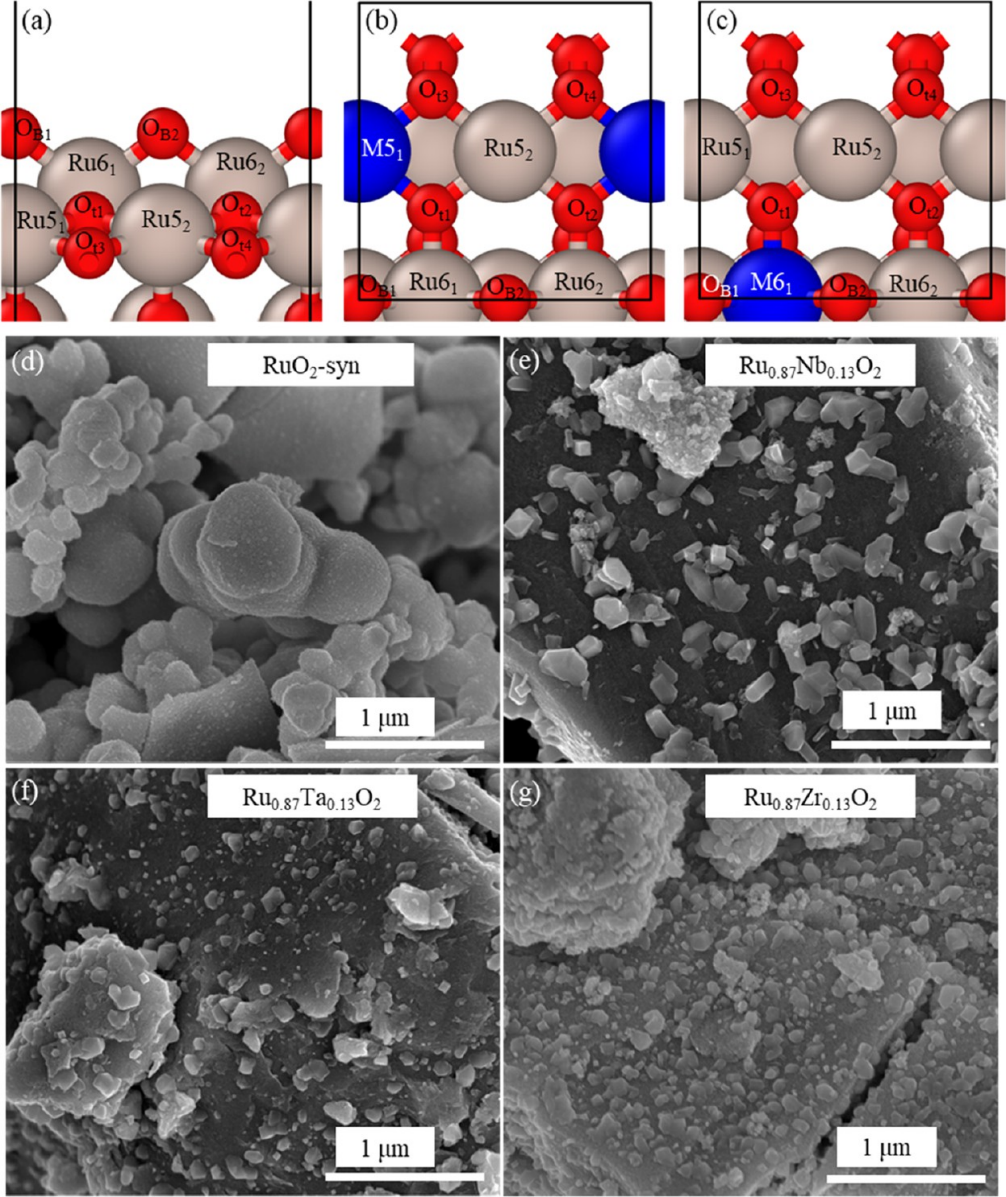
Top: (a) Pristine RuO_2_ surface and Ru_1–*x*_M_*x*_O_2_-doped surface models for (b) 25% M-5D and (c) 25% M-6D slabs. For each surface, starting from the left, sites are notated as Metal–Coordination_Site Number_ where Metal = Ru or M, coordination = 5 or 6, and Site Number = 1–2 (1–4 for O_t_ sites). Color code: red, oxygen; silver, ruthenium; blue, M species (M = Zr, Nb, Ta). Bottom: scanning electron microscopy (SEM) images of (d) RuO_2_-syn, (e) Ru_0.87_Nb_0.13_O_2_, (f) Ru_0.87_Ta_0.13_O_2_, and (g) Ru_0.87_Zr_0.13_O_2_.

For all surface calculations, the bottom layer, i.e., the lowest four metal atoms and eight oxygen atoms are fixed. From this slab, metal sites on the top layer were substituted with M species (M = Zr, Nb, Ta) to achieve 25 and 50% atomic concentration. However, computational analysis showed a high degree of instability at the highest concentration and our previously reported work on Ti substitution within RuO_2_ showed clear phase separation at 50 atom % metal substitution;^44^ so, hereafter, we report the results only for the 25% substituted surface slabs, as shown in the top row of Figure 1. We use the nomenclature 5D and 6D to indicate the surfaces where M is substituted on the penta- and hexa-coordinated sites, respectively, and to describe specific sites within each of the slabs, we used the notation Metal–Coordination_Site Number_ where Metal = Ru or M, coordination = 5 or 6, and Site Number = 1 or 2. Activation energy and transition-state (TS) calculations were performed using the climbing-image nudged elastic band (CI-NEB) method,^88−90^ while the charge transfer was evaluated via the Bader charge analysis.^91−93^ As complementary analysis, we carried out the integrated projected crystal orbital Hamilton population (IpCOHP) calculations, as allowed by the local orbital basis suite toward electronic-structure reconstruction (LOBSTER)^94−97^ software package, to better understand the effect for doping Ru_1–*x*_M_*x*_O_2_ (M = Zr, Nb, Ta) in M–O bonding interactions.

### 2.2 Experimental Methods

#### 2.2.1 Chemicals

Zirconium(IV) isopropoxide isopropanol complex (Zr(OCH(CH_3_)_2_)_4_·(CH_3_)_2_CHOH, 99.9%), niobium(V) ethoxide (Nb(OCH_2_CH_3_)_5_, 99.95%), tantalum(V) ethoxide (Ta(OCH_2_CH_3_)_5_, 99.98%), ammonia solution (NH_3_, 2.0 M in ethanol), and Nafion perfluorinated resin solution (5 wt %, 15–20% water) were obtained from Sigma-Aldrich. Ruthenium(III) nitrosyl nitrate (Ru 32.3%) was obtained from Alfa Aesar. Anhydrous ethanol (200 proof, ≤0.2% water content) was obtained from Pharmco-Aaper. Ultrapure water (≥18 MΩ·cm) from an ELGA water purification system was used throughout this investigation. All electrochemical measurements were carried out in 0.1 M HClO_4_ diluted from 70% HClO_4_ (Veritas Double Distilled, 0.000001% Cl^–^).

#### 2.2.2 Material Synthesis

Synthesized ruthenium oxide and M-substituted (M = Zr, Nb, Ta) ruthenium oxide, Ru_*x*_M_1–*x*_O_2_ (*x* = 0.125) are notated as RuO_2_-syn, Ru_0.87_Nb_0.13_O_2_, Ru_0.87_Ta_0.13_O_2_, and Ru_0.87_Zr_0.13_O_2_ (the notation Ru_0.87_M_0.13_O_2_ was implemented to simplify the stoichiometric representation). The 12.5 atom % M concentration was selected on the basis of our previous analysis on Ti substitution, where we found that the 12.5 atom % Ti material had a higher OER activity compared with materials using higher substituent concentrations.^44^ In addition, for a number of Ru-metal oxide mixtures, phase separation is observed at high substituent concentrations.^40,63,67^ The materials were synthesized by modification of a hydrothermal reaction previously reported.^44^ Inside an Ar-filled glovebox ([H_2_O] ≤ 1 ppm), 0.87 mmol of ruthenium nitrosyl nitrate was dissolved in 10 mL of anhydrous ethanol and magnetically stirred for 30 min. Then, 0.125 mmol of zirconium(IV) isopropoxide isopropanol complex, niobium(V) ethoxide, or tantalum(V) ethoxide were added and stirred for an additional 30 min. The resulting solution, along with the magnetic stir bar, was transferred into a plastic centrifuge tube and capped with a rubber stopper. The centrifuge containing the solution was brought outside the glovebox and was hydrolyzed by quickly adding 20 mL of a freshly prepared 0.25 M NH_3_/ethanol solution (2.5 mL of 2 M NH_3_/ethanol and 17.5 mL of anhydrous ethanol) using a syringe and was stirred for 5 min. Following this, 4.4 mL of 30% hydrogen peroxide (H_2_O_2_) was added at a rate of 0.2 mL/min using a syringe pump. A second needle was inserted to avoid overpressurization inside the plastic centrifuge tube. The solution turned into a brown milky consistency after the addition of hydrogen peroxide. Following the addition of H_2_O_2_, the solution was kept under stirring for 30 min. The as-prepared material was recovered using a Thermo Scientific Sorvall Lynx 6000 centrifuge at 5000 rpm (4304 RCF) for 5 min. The supernatant was decanted, and the precipitate was dissolved by adding 30 mL of ultrapure water. The aqueous solution was transferred to a poly(tetrafluoroethylene) (PTFE) hydrothermal reactor and then placed into a stainless steel (SS) jacket. The SS jacket was placed into a previously heated nest (130 °C) and kept under stirring for 190 min. The solution was allowed to cool to room temperature overnight. The precipitate was washed and collected using 40 mL of ultrapure water and centrifuged at 20,000 rpm (47,850 RCF) for 5 min. The precipitate was rinsed a total of three times and then dried overnight at 60 °C. The Ru_*x*_M_1–*x*_O_2_ precursor was calcinated in air in a Thermo Scientific Thermolyne small benchtop muffle furnace at 450 °C for 30 min using a a ramp rate of 10 °C/min. The sample was slowly cooled inside the muffle furnace before recovering. The synthesized, thermally treated samples are noted as described above.

#### 2.2.3 Physical and Structural Characterization

Powder X-ray diffraction (XRD) measurements were conducted using a Bruker AXS D8 Advance powder X-ray diffractometer with a Cu Kα (λ = 1.5406 Å) radiation source, operating at 40 kV and 25 mA and a high-resolution energy-dispersive 1D Linxeye XE detector. PDXL software (Rigaku) was used to analyze the diffraction patterns and perform Rietveld refinement to determine crystallographic constants and crystallite sizes. Rietveld refinement was conducted on XRD data in the 20–120° 2θ range, obtained using a slit width of 0.1 mm, a scan rate of 0.2°/min, and a step size of 0.02°. The rutile RuO_2_ powder diffraction file number 01-071-482 (International Centre for Diffraction Data) was used as a reference. For Rietveld refinement, the diffraction profile was fitted using a pseudo-Voigt peak shape, and the lattice parameters, profile, occupancies, Lorentz polarization correction, and measurement conditions were refined to obtain an optimized structure. Rietveld fitting utilized a tetragonal rutile phase with the space group *P*4_2_/*mnm*, point group *D*_4*h*_^14^, and two molecular formula units per unit cell (*Z* = 2). Initially, site occupancies were set to the metal atomic ratios obtained from energy-dispersive X-ray spectroscopy measurements (described below) and were allowed to vary during the refinement process. The quality of the Rietveld fitting is supported by the fit parameters of a residual whole pattern (*R*_wp_) < 10%, low residual profile (*R*_p_), goodness of fit (*S*) < 2, and χ^2^ of ∼1,^98^ and good agreement between the experimental powder patterns and the calculated patterns (Figure S2).

The morphology and elemental distribution of the synthesized Ru_*x*_M_1–*x*_O_2_ (M = Zr, Nb, Ta) were determined by scanning electron microscopy (SEM) and energy-dispersive X-ray spectroscopy (EDS) using a SEM FEI-Helios Nanolab 400. The samples were prepared for SEM/EDS analysis by suspending the samples in isopropyl alcohol and drop-casting the suspension onto an aluminum low-profile SEM pin mount holder. X-ray photoelectron spectroscopy (XPS) analysis was conducted using a Thermo Scientific Nexsa G2 Surface X-ray Photoelectron Spectrometer System with a monochromatic Al K_α_ X-ray source (1486.6 eV) that was focused to give a spot size of 400 μm diameter onto the sample within a copper powder sample holder. Survey and high-resolution spectra were collected using an analyzer, operated in Constant Analyzer Energy mode, CAE, with pass energies of 200 and 10 eV, respectively, for valence and core regions. Data analysis was performed using AVANTAGE v5.91 software (Thermo Fisher Scientific) using a Shirley-type background subtraction and a pseudo-Voigt function with Gaussian (70%)–Lorentzian (30%) for each component. XPS analysis was carried out from multiple independent batches of each catalyst material. Calibration of XPS binding energies using the C 1s peak was not utilized due to the significant overlap of the C 1s and Ru 3d regions and the low concentration of carbon within the materials. Therefore, the binding energies of all XPS peaks were calibrated using the binding energy of Ru 3p peaks from previous reported values for RuO_2_.^99^

#### 2.2.4 Electrochemical Characterization

Electrochemical measurements were conducted at 25 °C in a three-electrode cell using a thin-film rotating disk electrode (TF-RDE) configuration in a 0.1 M HClO_4_ electrolyte solution, following best practices.^100^ The electrochemical data were collected using an Autolab PGSTAT128N potentiostat and rotation control (Pine Instruments). A gold disk electrode (Pine Research, 0.196 cm^2^) was polished with alumina (0.05 μm) and was used as the substrate. The catalyst layer was prepared by pipetting 10 μL of an aqueous suspension catalyst (0.5 μg_catalyst_/μL), giving a catalyst loading of 25.5 μg_catalyst_/cm_geo_^2^. The electrode was mounted in an inverted RDE shaft and dried under rotation (500 rpm) at room temperature. After drying, 10 μL of a water/Nafion solution (5 wt % Nafion), diluted 100/1 by volume, was pipetted onto the catalyst layer and dried under the same conditions. A Pt mesh and a freshly prepared reversible hydrogen electrode (RHE) were used as counter and reference electrodes, respectively. The electrochemical surface area (ECSA) was determined using the double layer capacitance (DLC) technique by cycling at different scan rates (50, 40, 30, 20, 10 mV/s) in the range of 1.18–1.25 V_RHE_. The average of the absolute value of the anodic and cathodic current (*A*) at 1.15 V_RHE_ was plotted versus the scan rate (V/s), and the calculated slope was assigned as the double layer capacitance in Faradays (*F*).^101^ A Coulombic charge of 0.035 mF/cm^2^ was used as the conversion factor.^102^ The OER activity was evaluated using linear scan voltammetry (LSV) by sweeping at 20 mV/s from 1.2 to 1.6 V_RHE_ under rotation (2500 rpm) and the current at 1.51 V_RHE_ was used to calculate the OER activity, which was normalized by both ECSA (specific activity) and catalyst mass (mass activity). The ohmic drop (∼14 Ω) from the electrolyte was compensated by the potentiostat. The background current from the bare Au electrode, estimated under the same operating conditions, was subtracted from LSV and DLC calculations. The accelerated durability test (ADT) was performed at a constant 1.6 V_RHE_ (*iR*-corrected) potential while rotating at 2500 rpm for 13.5 h. In addition to the synthesized material, a commercially obtained RuO_2_ (Thermo Fisher, Cat. no: A10816.06, Lot: 10245212), notated as RuO_2_-com, was also tested. Following the ADT protocol, the solution was replaced with fresh electrolyte, and the ECSA and oxygen evolution activity measurements were repeated using the same electrochemical parameters described above. The electrolyte solution collected following the ADT was analyzed using an Agilent 8900 Triple Quadrupole inductively coupled-plasma mass spectrometer (ICP-MS) to determine the amount of Ru and Zr dissolved during the ADT. Ru and Zr standard solutions were obtained from Agilent and used for ICP-MS calibration. The stock standard solutions were diluted using a 3% HNO_3_ matrix solution (prepared from 70% HNO_3_, Aristar Ultra). An appropriate amount of 70% HNO_3_ was added to the electrolyte samples to obtain an overall 3% HNO_3_ matrix solution, as was used for the standard solutions. MassHunter software was used for ICP-MS data acquisition and analysis.

## 3 Results and Discussion

### 3.1 Substitution Effects on the Crystalline Structure, Morphology, and Elemental Composition of Ru_1–*x*_M_*x*_O_2_ (M = Zr, Nb, Ta)

The effect of substituents on the structure of Ru_1–*x*_M_*x*_O_2_, (M = Zr, Nb, Ta) was evaluated using computational and experimental analyses. For our computational model, the slab’s top layer is formed by four Ru atoms (two penta- and two hexa-coordinated) and six O atoms (two bridge, O_B_, being the most exposed oxygen atoms on the surface, and four tricoordinated, O_t_, at the same height level as the metal atoms), as shown in the top row of Figure 1a–c. It is known that a perfectly cleaved (110) rutile-type oxide has all metal sites on the surface saturated, which results in two types of oxygen atoms on the surface: oxygens in bridge sites (O_B_, two-coordinated) and coordinatively unsaturated sites (O_CUS_, one-fold coordinated).^26,103^ However, the O_CUS_ species were not included in our model because they tend to react with water or among them after thermal treatment to form molecular oxygen, which will produce a vacancy, leaving exposed the penta-coordinate metal active sites for water adsorption.^103,104^ We modeled substitution of Zr, Nb, and Ta in the bulk Ru_1–*x*_M_*x*_O_2_ structure at three atomic concentrations: 12.5, 25, and 50%.

**Figure 2:**
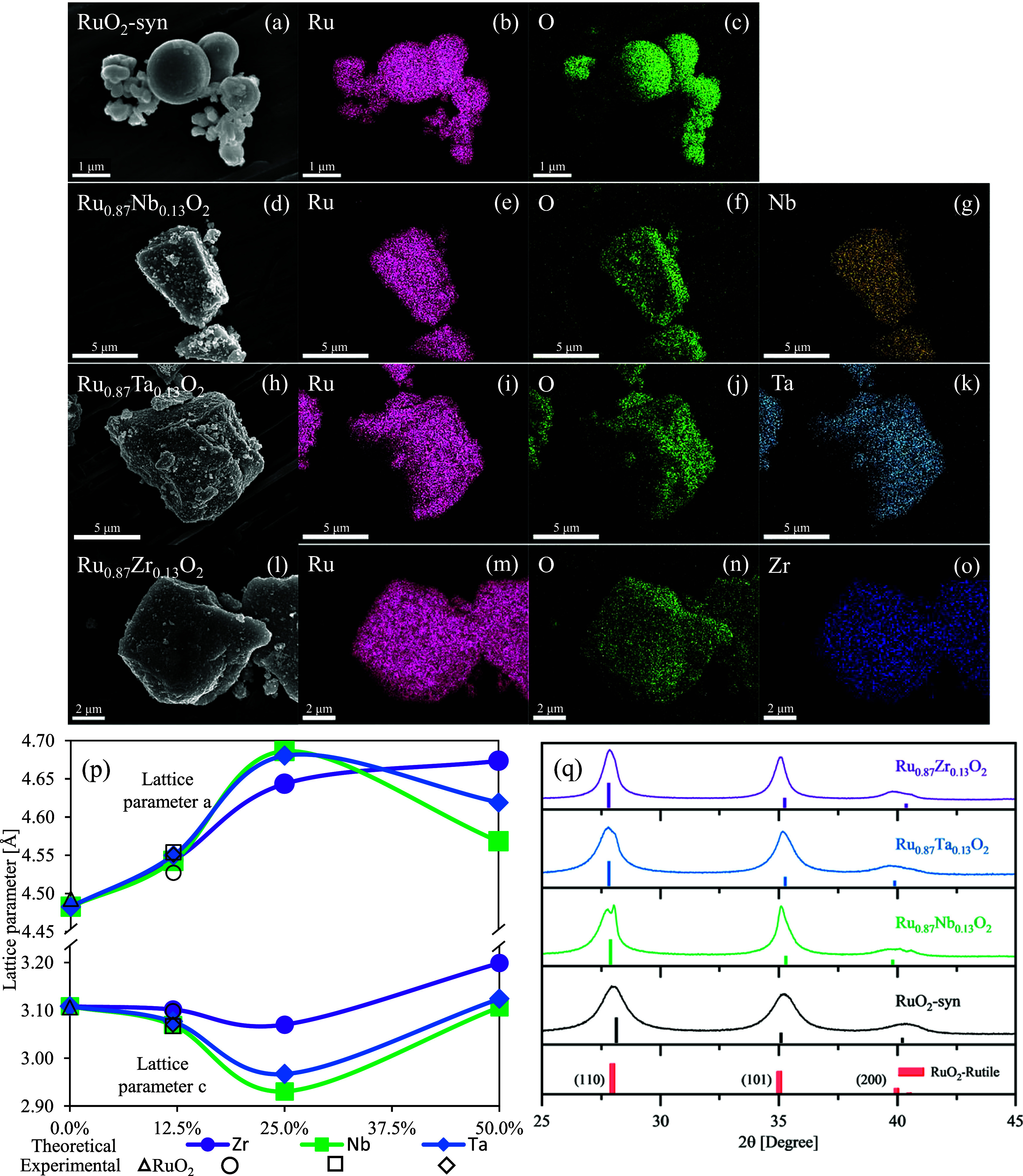
Scanning electron microscopy (SEM) images and energy-dispersive X-ray spectroscopy (EDS) elemental mapping analysis of (a–c) RuO_2_-syn, (d–g) Ru_0.87_Nb_0.13_O_2_, (h–k) Ru_0.87_Ta_0.13_O_2_, and (l–o) Ru_0.87_Zr_0.13_O_2_ showing distribution of ruthenium, oxygen, niobium, tantalum, and zirconium within the structures. Effect of metal substituents on the crystalline structure: (p) “*a*” and “*c*” lattice parameter evolution after M (M = Zr, Nb, Ta) addition into the Ru_1–*x*_M_*x*_O_2_ bulk system for the theoretical (filled shapes, continuous lines) and experimental (unfilled shapes) systems; (q) experimental (soft lines) and theoretical (vertical lines) powder X-ray diffraction (XRD) patterns of RuO_2_-syn (black), Ru_0.87_Nb_0.13_O_2_ (green), Ru_0.87_Ta_0.13_O_2_ (blue), and Ru_0.87_Zr_0.13_O_2_ (purple) and patterns for rutile RuO_2_ reference (PDF: 01-071-4825).

We synthesized Ru_1–*x*_M_*x*_O_2_, (M = Zr, Nb, Ta) using a hydrothermal method adapted from our previously reported study.^44^ The Ru_1–*x*_M_*x*_O_2_ samples were imaged using scanning electron microscopy (SEM), and representative images are shown in Figure 1d–g. SEM images for the synthesized RuO_2_ (RuO_2_-syn) show a distribution of particle sizes ranging from ∼0.25 to 1 μm in diameter. The change in morphology observed once 12.5 atom % of Nb, Ta, or Zr is added to the RuO_2_ material is attributed to a change in the crystal nucleation and growth mechanism during the condensation reaction in the synthesis. The condensation reaction can be influenced by the pH, different metal electronegativities, and the type of precursors used and these factors play a critical role on the crystal structure, size distribution, and morphology obtained.^105^ The SEM images suggest that the addition of Nb, Ta, or Zr to RuO_2_ increases the particle size when compared to pure RuO_2_. SEM images of Ru_0.87_Nb_0.13_O_2_, Ru_0.87_Ta_0.13_O_2_, and Ru_0.87_Zr_0.13_O_2_ show large agglomerates (∼5 μm) composed of smaller particles with diameters of about 50–200 nm. From the elemental mapping images obtained from energy-dispersive spectroscopy (EDS) of the SEM images, Figure 2a–o shows a uniform distribution of Ru, O, and Nb, Ta, or Zr throughout the material at the resolution of the EDS mapping. The bulk compositions of Ru_0.87_Nb_0.13_O_2_, Ru_0.87_Ta_0.13_O_2_, and Ru_0.87_Zr_0.13_O_2_ obtained from EDS are summarized in Table S1. The Zr atomic percent calculated from EDS analysis (Ru_0.88_Zr_0.12_) is comparable to the nominal synthesis ratio (Ru_0.875_Zr_0.125_). For the Ru_0.87_Nb_0.13_O_2_ and Ru_0.87_Ta_0.13_O_2_ samples, EDS analysis showed a higher atom % of Nb (Ru_0.82_Nb_0.18_) and Ta (Ru_0.76_Ta_0.24_) when compared to the nominal synthesis ratios (Ru_0.875_Nb_0.125_ and Ru_0.875_Ta_0.125_). Differences observed between atom % values obtained from EDS and nominal synthesis ratios can be explained by a loss of Ru during the thermal treatment due to the formation of volatile ruthenium tetroxide (RuO_4_)^106^ and the differences in hydrolysis and condensation rates of the Ru, Nb, Ta, or Zr precursors. It has been observed that the oxidation states of the metals and the steric hindrance from the ligands can affect the reactivity towards hydrolysis.^107^

We used computations and experiments to evaluate the effect of substituents on the crystalline structure of Ru_1–*x*_M_*x*_O_2_ (M = Zr, Nb, Ta), which provides a basis to understand the materials’ OER activity and electrochemical stability. The calculated evolution of the main lattice parameters, “*a*” (= “*b*”) and “*c*”, as a function of the M substituent species concentration is shown in Figure 2p. The patterns suggest that the structure experiences a progressive rearrangement allowing the lattice parameters “*a*” and “*b*” to elongate (*a* = *b* within the rutile unit cell), while the parameter “*c*” is reduced at the relative low substituent concentrations of 12.5 and 25%. However, this rearrangement is highly dependent on substituent concentrations. At 25% of Zr substitution, the system exhibits the smallest reduction of 1.23% with respect to the “*c*” parameter of pristine RuO_2_ (*c* = 3.070 Å), while the largest effect is at 50%, with a “*c*” elongation of up to 3.199 Å (2.91% larger than in RuO_2_). Nb and Ta substitutions show a similar behavior, having the largest effect in the bulk structure at 25% atomic concentration, with “*c*” lattices of 2.930 and 2.967 Å (5.73 and 4.55% shorter), respectively, while at 50% their “*c*” values are 3.106 and 3.124 Å, which are larger than at other concentrations and almost unaltered from the original bulk structure. The “*a*” lattice constant increases with the presence of the substituent, the largest effect is found at 25% M concentration (4.644, 4.686, and 4.680 Å for Zr, Nb, and Ta, respectively, compared to 4.483 Å for RuO_2_). Interestingly, the increasing trend is reversed for Nb and Ta that at 50% show a relative reduction in the “*a*” parameter with respect to lower concentrations. The nonlinear behavior of the lattice parameters of Ru_1–*x*_M_*x*_O_2_ (M = Zr, Nb, Ta) with metal concentration (Figure 2p) is in line with a prior study of Ru_1–*x*_Si_*x*_O_2_ solid solutions, which determined opposite nonmonotonic trends between the “*a*” and “*c*” lattice parameters.^108^

**Figure 3:**
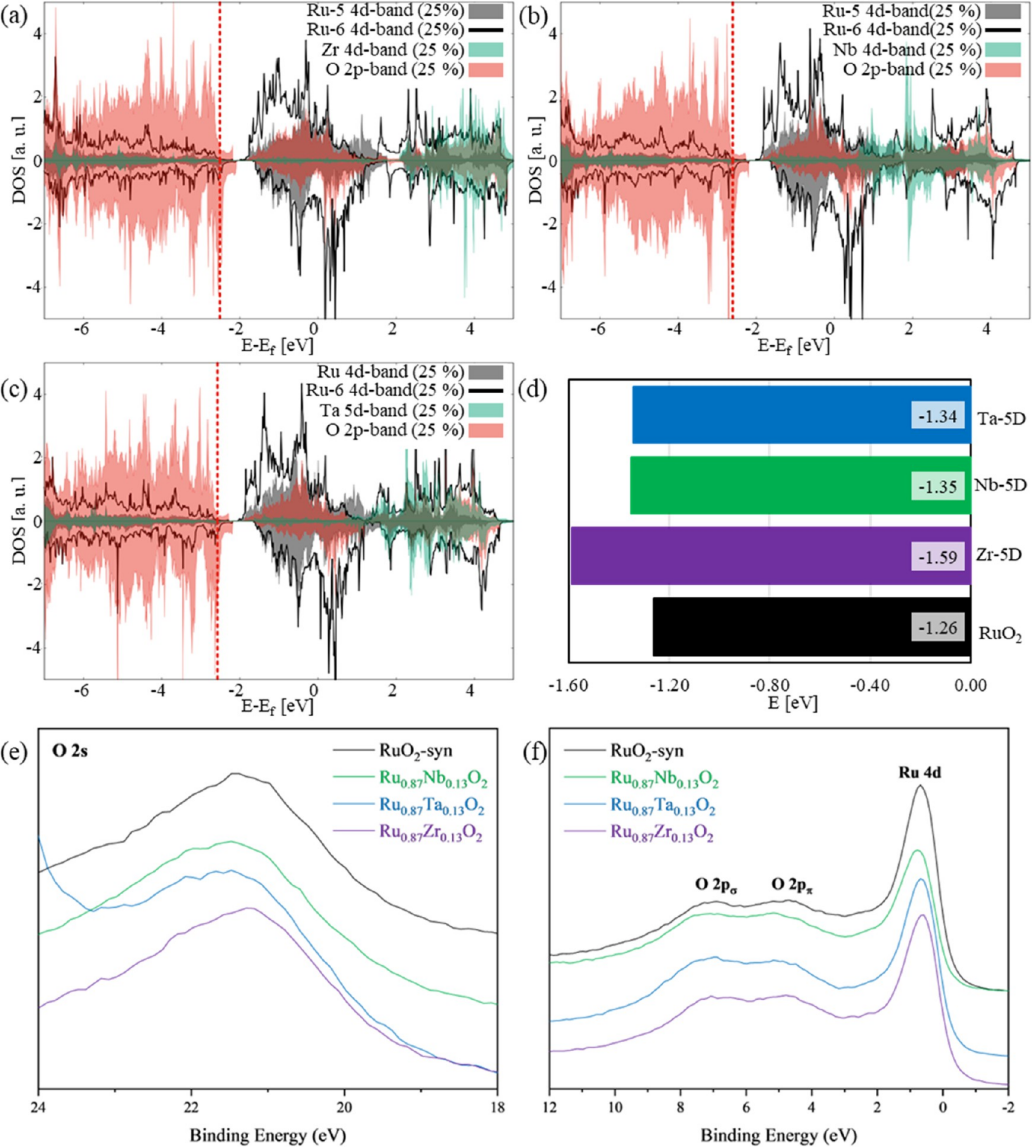
Contributions of Ru-5C-4d, Ru-6C-4d, O-2p, and M-5D-d (Zr-4d, Nb-4d, and Ta-5d) bands to the (110) top layer density of states (TDOS), respectively, for each system at 25% M-5D. The Ru-5C-4d band is represented in gray, Ru-6C-4d band in solid black line, O-2p band in red, and M-d band in green for (a) Ru_0.75_Zr_0.25_O_2_, (b) Ru_0.75_Nb_0.25_O_2_, and (c) Ru_0.75_Ta_0.25_O_2_. The vertical red dashed line in each figure represents the O-2p band center;(d) 4-d band centers for all systems; experimental X-ray photoelectron spectra (XPS) of RuO_2_, Ru_0.87_Nb_0.13_O_2_, Ru_0.87_Ta_0.13_O_2_, and Ru_0.87_Zr_0.13_O_2_ in the (e) O-2s region and (f) valence band region.

The experimental and XRD patterns of RuO_2_-syn, Ru_0.87_Nb_0.13_O_2_, Ru_0.87_Ta_0.13_O_2_, Ru_0.87_Zr_0.13_O_2_, and a RuO_2_ rutile reference pattern are shown in Figure 3b, and the 2θ° positions obtained from the DFT calculated structures are also presented. The full-range XRD patterns, Rietveld-fitted patterns and refinement details, lattice parameters, and experimental and theoretical values of the (110), (101), and (200) planes are provided in Figures S1, S2 and Tables S2–S4. The XRD peaks and Rietveld fitting of the experimental materials are consistent with the material adopting a tetragonal rutile phase or phases. The RuO_2_-syn material exhibited XRD peaks consistent with a single phase, which is also supported by Rietveld fitting. For the metal-substituted materials, Rietveld fitting analysis determined the presence of two phases: (i) a majority phase that contained the metal substituent within the rutile phase with a similar atomic concentration as observed from EDS analysis and (ii) a smaller-percentage second RuO_2_ phase that did not contain the metal substituent (Tables S3 and S4). The Ru_0.87_Nb_0.13_O_2_ sample showed the presence of two distinct (110) peaks and had the highest degree of phase separation. For Ru_0.87_Ta_0.13_O_2_, fitting analysis identified a majority phase (77 phase %) containing Ta. The Ru_0.87_Zr_0.13_O_2_ sample showed the predominate phase (90%) contained Zr, and the material had a small percentage (10%) of a separate RuO_2_ phase. The different percentages of the phases within the metal-substituted materials may be related to the substituents’ effect on hydrolysis and condensation reaction and crystal growth rates, as discussed above. From Rietveld refinement, we observed larger crystallite sizes for Ru_0.87_Nb_0.13_O_2_, Ru_0.87_Ta_0.13_O_2_, and Ru_0.87_Zr_0.13_O_2_ when compared to RuO_2_-syn, which may result from differences in growth kinetics. Lattice parameters of the majority phase containing the metal substituent are presented in Figure 2p, and lattice parameters for all phases are presented in Table S3. The existence of two phases within metal-substituted materials complicates the structural and electrochemical analysis; however, we focus our analysis and discussion on the majority phase, which contained the substituent metal within the rutile phase.

From the experiments, the lattice parameters determined from Rietveld refinement of the predominant phase show an expansion of the “*a*” (and “*b*”) lattice parameter and a compression of the “*c*” lattice parameter for the substituted samples (Figure 2p), in agreement with our theoretical analysis. The theoretical XRD patterns and the calculated 2θ° values (vertical color-matched lines) are shown in Figure 2q. The asymmetric changes in the unit cell (generally elongation of “*a*” (and “*b*”) and compression of “*c*” parameter) due to the substituents are reflected in the theoretical XRD. The comparison of the theoretical and experimental 2θ° values is shown in Table S2. The theoretical analysis shows that the main (110) plane peak shifts to the left in all structures, whereas for the systems with Nb and Ta, the largest shift occurs at 25%, in agreement with the largest “*a*” lattice expansion and “*c*” contraction. For the (101) plane, Ru_1–*x*_Zr_*x*_O_2_ is shifted to the right compared to the original RuO_2_ oxide at the lowest concentration evaluated (12.5%); however, it becomes left-shifted as the concentration of Zr increases. On the other hand, the (200) plane shows a constant left shift in all cases.

The interesting shifts in lattice parameters with metal-substituent concentration observed from both experimental and computational analysis (Figure 2p) do not follow linear trends (i.e., Vegard’s law)^109^ related to the different metals’ ionic radii (62 pm for Ru^4+^, 64 pm for Nb^5+^ and Ta^5+^, and 72 pm for Zr^4+^).^110^ Numerous examples of deviations from linear trends of lattice parameters with concentration have been previously reported,^108,111−113^ and a nonlinear behavior has been attributed to how electron density is distributed within the lattice.^112,113^ Our analysis, presented in the following section, supports that understanding anisotropic changes to the lattice parameters requires consideration of how the substituents within specific substitution sites alter electron density, which induces changes into the geometric structure.

### 3.2 Substitution Effects on the Electronic Structure of Ru_1–*x*_M_*x*_O_2_ (M = Zr, Nb, Ta)

#### 3.2.1 Effect of Substituents on the Calculated Total Density of States

Figures S3–S5 summarize the contributions of the Ru-4d, O-2p, Zr-4d, Nb-4d, and Ta-5d bands to the TDOS, respectively, for each bulk system at 12.5, 25, and 50% substituent concentrations. It is found that the effect of each substituent species M (M = Zr, Nb, Ta) on the Ru and O electronic structure depends on their intrinsic properties as well as on their concentration. For example, in Zr-substituted Ru systems, both the 4d states of the Ru atoms and the O-2p of the oxide become spin-polarized, while the spin polarization features are less affected by Nb substitution, although for both Zr and Nb, there is low contribution of d-electron population. Table S4 shows the values of the Ru-4d band and O-2p band centers theory calculated for each bulk system including 12.5, 25, and 50 atom % substitution ratios. Note that the d band center shifts to left and right of the Fermi level are usually associated with lower or higher reactivity, respectively.

The DOS spectra (Figures S3–S5) show that the general conducting characteristic (no band gap) from pristine RuO_2_ is not altered by the introduction of the substituent, but the effect of the different species is observed in the reduction of the states’ population on and below the Fermi energy (smaller spectra), especially for Zr and Ta. Interestingly, common patterns among the different substituent species can be found. For instance, Zr and Ta reduce the electron density population on and below the Fermi level at high surface concentrations while inducing an important extent of spin polarization in the spectra, as clearly observed in the green patterns in Figure S5a–c (Zr, Nb, and Ta at 50%, respectively). On the other hand, Nb induces a less severe effect on the d-population of the system, as shown in Figure S5b. Nb and Ta share a similar behavior regarding the shift of the Ru-4d band centers (ε_d_). At lower substituent concentrations, i.e., at 12.5 and 25%, the d band centers shift progressively to the left toward higher binding energies (−1.19 and −1.40 eV for Nb; – 1.19 and −1.39 eV for Ta, respectively); however, at 50% concentration, the shift is to the right relative to the value in the RuO_2_ oxide (−0.98 and −0.96 eV, respectively). Hence, the effect of these species on the electronic structure is highly dependent on their concentrations, similar to the effects observed in the geometric structure. On the other hand, Zr follows a similar trend as Ti regarding the d band center value,^44^ where the Ru-4d band center shifts progressively toward higher binding energies as the dopant species concentration increases (from −1.24 to −1.32 eV for Zr, respectively). In summary, compared to the pristine oxide, the d band center values suggest overall less reactive Ru sites due to the presence of the substituents.

Regarding the effect of the substituent species on the O-2p band, the centroid of the projected density of states of the O-2p orbitals relative to the Fermi energy, or O-2p band center, has been used as an electronic descriptor for a variety of properties in metal oxides.^114,115^ The O-2p band center values calculated for the Ru_1–*x*_M_*x*_O_2_ bulk systems are shown as vertical red, dashed lines in Figures S3–S5 and included in Table S5. Following the same trend as the d band, the O-2p band center behavior is highly dependent on the substituent species and their concentrations. Zr, for example, induces changes toward higher (less negative) values when the atomic concentration is 12.5% (−2.99 or 1.97% higher p-band center compared to −3.05 from RuO_2_) and 50% (increment to −2.89 eV p-band center), with the largest shift being a product of the higher concentration, while at 25% the p-band center value is almost identical as the one for the original oxide (−3.04), with a small increment of only 0.33% compared to the original oxide. On the other hand, Nb and Ta behave similarly in the sense that the p-band center remains almost unaltered at the lowest concentration of 12.5% (−3.04 and −3.07, respectively); however, as their concentration increases, the p-band center moves toward lower values (more negative) with values shifted up to 0.61 and 0.85 eV for Nb and Ta at 50%, respectively (p-band center down to −3.66 and −3.90 eV, respectively).

To better understand the catalytic activity, it is important to analyze the surface electronic structure. Figure 3a–c shows the contributions of the Ru-5C-4d, Ru-6C-4d, O-2p, and M-5D-d (Zr-4d, Nb-4d, and Ta-5d) bands to the Ru_0.75_M_0.25_O_2_-5D(110) top layer density of states (TDOS), respectively, for each surface system, while Figures S6 and S7 summarize the top layer Ru-5C- and 6C-4d, O-2p, Zr-4d, Nb-4d, and Ta-5d bands contributions to the surface TDOS for the Ru_0.75_M_0.25_O_2_-6D(110) and Ru_0.5_M_0.5_O_2_-(110) surfaces, respectively, and the calculated values for the Ru-5C 4d band and O_B_ 2p band centers are included in Table S5. These surface calculations reveal that only Zr-6D sites induce Ru-5C d band centers that shift toward higher reactivity regions, compared to Zr-5D sites, while Nb- and Ta-6D shift the d band center descriptors to lower values (higher binding energies) compared to the same species on the 5D sites. However, the d band centers obtained from substituting Zr on the surface are the lowest among the other species and compared to the original RuO_2_ oxide. Note that the left shift in the d band centers signals a reactivity decrease. However, there are two competing reactions: catalysis and corrosion, our further analyses are oriented to determine which of these two reactions are more impacted by the change in the electronic behavior. On the other hand, all species exhibit the same shift trends for the O_B_ p-band centers: higher values obtained from the 6D surfaces (higher than in the pristine surface) and lower ones from the 5D slabs.

#### 3.2.2 X-ray Photoelectron Spectroscopy

To complement our computational analysis of the effect of the metal substituents on the surface electronic structure, we analyzed the experimentally synthesized material using X-ray photoelectron spectroscopy (XPS). XPS analysis was conducted on the core-level and valence band regions at the surface region of RuO_2_-syn, Ru_0.87_Nb_0.13_O_2_, Ru_0.87_Ta_0.13_O_2_, and Ru_0.87_Zr_0.13_O_2_ catalysts. Analysis of the surface composition of the catalysts from the XPS survey spectra (Figure S8) showed a higher atomic percent of the metal substituents at the surface (Ru_0.56_Nb_0.44_O_2_, Ru_0.60_Ta_0.40_O_2_, and Ru_0.63_Zr_0.37_O_2_) when compared to the intended nominal synthesis loading and bulk composition determined from EDS analysis (Table S1). The higher concentrations of the substituents on the surface can be due to the different hydrolysis and condensation rates of the metal precursors and/or different surface energies, oxophilicity, or diffusion-coalescence processes generated during the thermal treatment that promote the migration of different metals to the surface.^116,117^ Considering the differences between bulk and surface compositions from EDS and XPS analyses shown in Table S1, the surface calculations are carried out on the 25% substituted surfaces, as a representative model of the actual surface atomic concentration. We note that the XPS experiment probes not just the topmost atomic layer but also atoms below the top surface layer (the inelastic mean free path of excited photoelectrons is on the order of ∼1–10 nm). Thus, as a compromise solution, we used the 25% substituent concentration for the top two surface layers in our computational studies. We note that the model assumes accumulation of the new element on the surface but does not assume the formation of a new phase.

**Figure 4:**
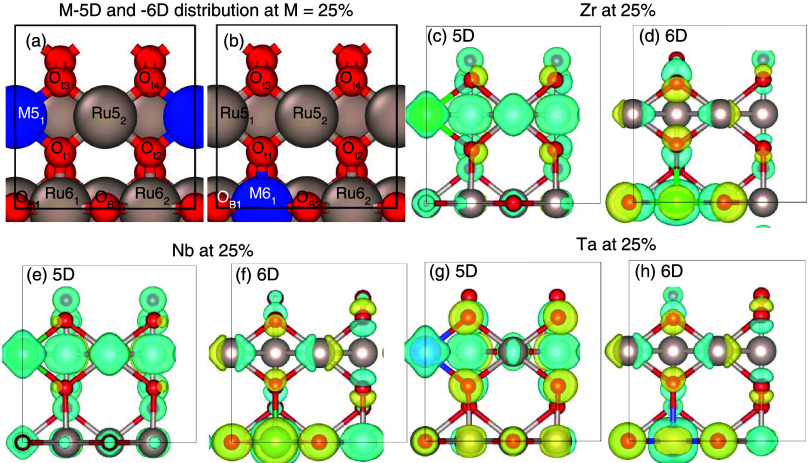
(a) M-5D and (b) M-6D surface distribution; electron density difference showing electron density accumulation (yellow) and depletion (light blue) for (c) Zr-25%-5D, (d) Zr-25%-6D, (e) Nb-25%-5D, (f) Nb-25%-6D, (g) Ta-25%-5D, and (h) Ta-25%-6D slabs doped at 25% concentration, respectively.

XPS spectra of the O-2s bands of RuO_2_-syn, Ru_0.87_Nb_0.13_O_2_, Ru_0.87_Ta_0.13_O_2_, and Ru_0.87_Zr_0.13_O_2_ are shown in Figure 3e and tabulated in Table S6. The binding energy of the O-2s peak of RuO_2_-syn appears at 21.2 eV, and the O-2s peaks of Ru_0.87_Nb_0.13_O_2_ (21.3 eV) and Ru_0.87_Nb_0.13_O_2_ (21.5 eV) are shifted to higher binding energies. Conversely, the incorporation of Zr shifts the O-2s to a lower binding energy (21.1 eV). The XPS valence band regions of RuO_2_-syn, Ru_0.87_Nb_0.13_O_2_, Ru_0.87_Ta_0.13_O_2_, and Ru_0.87_Zr_0.13_O_2_ are shown in Figure 3f. Peaks in the XPS valence band region result from M–O σ bonding, π bonding, and π antibonding between the metal ions, Ru^4+^ ([Kr] 4d^4^), Zr^4+^ ([Kr]), Nb^5+^ ([Kr]), and Ta^5+^ ([Xe] 4f^14^), and O^2–^ ([He] 2s^2^2p^6^) and are assigned to O-2p_σ_, O-2p_π_, and Ru-4d peaks.^118,119^ The binding energies of the O-2p_σ_ and O-2p_π_ peaks of RuO_2_-syn, Ru_0.87_Nb_0.13_O_2_, and Ru_0.87_Ta_0.13_O_2_ are within the experimental error; however, within Ru_0.87_Zr_0.13_O_2_, the O-2p_σ_ and O-2p_π_ peaks are shifted to lower binding energies relative to RuO_2_-syn. From our XPS analysis of the oxygen (O-2s and O-2p) peaks, the presence of the higher oxidation state Nb^5+^ and Ta^5+^ substituents shift O-2s peaks to higher binding energies, while Zr^4+^ substitution results in shifts of O-2s and O-2p peaks to lower binding energies. The experimental shifts to higher binding energies (farther away from the Fermi level) for oxygen within the Nb^5+^- and Ta^5+^-substituted materials are consistent with our calculations of O-2p band center shifts at O_B_ and O_t_ atoms within the bulk structures, where they shift from −3.05 eV within the original RuO_2_, up to −3.66 and −3.90 eV within the Ru_1–*x*_M_*x*_O_2_ for M = Nb, and Ta at 50%, respectively, while experimental observation of a shift to lower binding energies for oxygens with the Zr^4+^-substituted material agrees with the calculated O-2p shifts closer to the Fermi level with values up to −2.89 eV for Zr-substituted bulk structures, as reported in the section above and in Figures S3–S5. Moreover, O-2p band center calculations for O_B_ atoms within the Ru_1–*x*_M_*x*_O_2_-(110) cleaved surface reveal that there is a specific effect according to the substituent site, i.e., 5D or 6D position, as reported in Table S5, similar to the electron density effects reported in Figure 4. We note that the calculated p-band center shift of the Ru_0.75_M_0.25_O_2_-6D-(110) surface is in agreement with the experimentally observed shift of O-2p peaks to lower binding energy for Ru_0.87_Zr_0.13_O_2_, which may suggest the prevalence of 6D substituted atoms within the Ru_0.87_Zr_0.13_O_2_ synthesized materials. The correlations between electronic-structure calculations of p-band center shifts and experimental shifts of XPS binding energies for O-2p peaks of Ru_0.87_Nb_0.13_O_2_ and Ru_0.87_Ta_0.13_O_2_ are complicated by the resolution of the XPS experiment and may be influenced by other factors (e.g., penetration depth, specific surface structure, etc.). Further analysis is needed to determine the surface sites of the synthesized materials.

We analyzed the binding energies of the Ru-4d peaks of the XPS valence band region and found the Ru-4d peaks of Ru_0.87_Nb_0.13_O_2_ and Ru_0.87_Ta_0.13_O_2_ are within the experimental error; however, the Ru-4d peaks of Ru_0.87_Zr_0.13_O_2_ are shifted to lower binding energies relative to RuO_2_. The shift to lower binding energies is in line with our calculations of sites that Zr substitutions show larger shifts on the Ru-5C 4d band centers. The comparison of the XPS binding energy shifts and calculated Ru-5C 4d band center and electron density changes suggest that specific metal and oxygen sites may be expressed within the synthesized materials; however, additional factors including the effects of surface facets and surface disorder on the electronic structure and differences between the surfaces of the calculated structures and experimentally synthesized materials need to be further considered.

We performed XPS analysis of core Ru 3d, Nb 3d, Ta 4f, and Zr 3d regions (Figure S9) and found binding energies that are consistent with the presence of Ru^4+^, Nb^5+^, Ta^5+^, and Zr^4+^ at the surface region of the catalysts. The Ru 3d core-level spectrum consists of two spin–orbit doublet peaks corresponding to Ru(IV) 3d_5/2_, 3d_3/2_, and their satellite peaks. The 3d_5/2_ and 3d_3/2_ peaks are located at 280.82 and 285.10 eV, respectively. The two Ru 3d_5/2_ and Ru 3d_3/2_ satellite peaks are located at 282.91 and 286.69 eV and are in good agreement with previous reported peak positions.^99,102,120^ The Nb 3d core-level spectrum consists of the Nb^5+^ doublet peaks located at 206.59 eV and 209.31 corresponding to the Nb 3d_5/2_ and 3d_3/2_ spin–orbit doublet, and their binding energies are in agreement with the previously reported values.^121−123^ The core-level XPS spectrum of the Ta 4f region shows a doublet that corresponds to Ta^5+^ 4f_7/2_ and 4f_5/2_ located at 25.55 and 27.38 eV, and is within the range of values reported in the literature.^124,125^ The core-level XPS spectrum of Zr 3d shows a doublet that corresponds to Zr^4+^ 3d_5/2_ and 3d_3/2_ located at 184.34 and 181.92 eV and is within the range of values reported in the literature.^126^ XPS spectrum of the O 1s region (Figure S10) of RuO_2_-syn showed asymmetric features that suggest a predominantly anhydrous RuO_2_ surface,^99^ and similar features consistent with an anhydrous surface were observed for Ru_0.87_Nb_0.13_O_2_, Ru_0.87_Ta_0.13_O_2_, and Ru_0.87_Zr_0.13_O_2_. An anhydrous surface was expected due to the thermal treatment performed during the synthesis. Significant binding energy shifts due to the substituents were not able to be resolved beyond experimental error.

To understand the substituent effect in M–O bonding interactions, we carried out integrated projected crystal orbital Hamilton population (IpCOHP) calculations. The IpCOHP results are summarized in Table 1. IpCOHP serves as an indicator of the bonding strength interaction between two specified atoms, i.e., the more negative the IpCOHP value, the stronger the atom pair interaction and the higher the energy cost to remove or separate any of the two species. From these results, it is found that Zr has the weakest bonding effect among all three dopant species, given by a less negative average value for the IpCOHP in the bulk structure. A similar although less severe effect is shown by Nb. On the other hand, Ta shows an inverse effect, where the average value for the IpCOHP becomes more negative, corresponding to stronger M–O interactions as the concentration of Ta increases.

**Table 1:** Average of the Integrated Projected Crystal Orbital Hamilton Population (IpCOHP) [eV] for Selected M–O Pairs in Bulk Structures

[M] (%)	Zr	Nb	Ta
0	–1.87	–1.87	–1.87
12.5	–1.66	–1.77	–1.97
25	–1.63	–1.78	–2.09
50	–1.43	–1.57	–2.11

Geometric and electronic analyses show that there is a complex relationship between substituent incorporation and their crystalline and electronic effects due to their intrinsic properties and the induced level of structural rearrangement. This phenomenon arises because the crystal structure and properties of metal oxides are directly linked to the arrangement of their basic MO*_n_* polyhedral building blocks.^127,128^ Atomic-level understanding of these rearrangements is crucial as the local distortions and defects on the MO*_n_* structure can alter intrinsic electrical properties and growth kinetics of metal oxides.^129,130^ The rutile structures evaluated here are composed by metal octahedrons (MO_6_) and the main point of distortion is the different octahedron size for each species, i.e., the M–O bond distances are slightly different for Ru, Zr, Nb, and Ta as can be inferred from the radial distribution functions shown in Figure S11. This dissimilarity induces an uneven repulsion between the anions/cations of the same charge (mainly O–O, and M–M at increasing concentrations of the substituent species).^131^ Recalling the previously analyzed Ti effect, in TiO_2_, the Ti^4+^ shows a distorted octahedral environment with four shorter Ti–O distances in the equatorial plane and two longer Ti–O distances in the axial plane (tetragonal elongation), whereas the contrary occurs for RuO_2_ that exhibits four longer equatorial Ru–O distances and two shorter axial ones (tetragonal compression).^45^ Intrinsic structural behavior has also been reported in ZrO_2_ compounds.^132^ The structural differences among them are correlated to the respective electronic configurations of the metal in each metal oxide, as even though the metal ion can be formally considered as M^4+^, it has no d electrons in the case of Ti^4+^ and Zr^4+^, whereas Ru^4+^ possesses four d electrons.

Inclusion of Nb and Ta adds a higher degree of complexity in the M–O–Ru geometrical configuration as, even though MO_2_ compounds have been obtained and stabilized, these species tend to form M_2_O_5_ polymorphous compounds, whose atomic configuration highly depends on the synthesis conditions, mainly temperature.^133−139^ Moreover, the electron population might be highly affected as these species tend to form Nb^5+^ and Ta^5+^ compounds, in contrast with the predominant Ru^4+^ from our structures. These differences induce a certain degree of covalent character, and in an octahedral ligand field, this also leads to distorted geometrical configurations.^45^

#### 3.2.3 Calculations of Surface Electronic Distribution

To complement the analysis of the substituent effects on the (110)-Ru_1–*x*_M_*x*_O_2_ surface, the calculated electronic density differences are shown in Figure 4. Because of the electronic difference between Ru and the substituent species, a significant electron accumulation (yellow) is obtained at the substituted sites (M atoms) and electron depletion (light blue) between the substituted sites and the neighbor O_B_ and O_t_ atoms, as well as on or close to the neighbor Ru-5 sites. Zr-5D has an important effect inducing a relevant degree of electron density depletion on the Ru-5 atom, as shown in Figure 4c. The effect is more pronounced on the metal sites as well as the O_t_ sites, while the density difference on the O_B_ sites is lower but still associated with electron density depletion. In contrast, the Zr-6D type (Figure 4d), induces a high degree of electron accumulation on top of the O_B_ sites, while it has an interesting lateral effect on the penta-coordinated metal sites according to the proximity of a specific neighbor. For example, there is a lateral electron density depletion (blue cloud) on the side of Ru-5 closer to Zr-6D, while there is electron density accumulation (yellow cloud) on the side further to the substituted Zr-6D atom. Interestingly, Nb has similar effects as the ones described from Zr (Figure 4e,f), with slight differences in the cloud density sizes. On the other hand, Ta induces a huge electron density accumulation effect on top of both O_t_ and O_B_ sites as well on Ru-6 while causing a small depletion on top of Ru-5, as shown in Figure 4g. Ta-6D causes similar effects as Zr- and Nb-6D (Figure 4h).

### 3.3 Electrochemical Oxygen Evolution Reaction Activity and Reaction Mechanism of M-substituted RuO_2_

#### 3.3.1 Effect of Metal Substituents on the OER Mechanism

Activity of the Ru_1–*x*_M_*x*_O_2_ (M = Zr, Nb, Ta) materials was evaluated through the calculation of a series of proposed OER reactions. Following our previous work,^44^ we describe the OER as an initial step defined by water adsorption and subsequent oxidation, followed by a series of oxygen evolution steps. The following set of equations summarizes the water oxidation and O_2_ formation steps considered in this study.

Water splitting steps
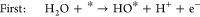
1
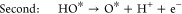
2

**Figure 5:**
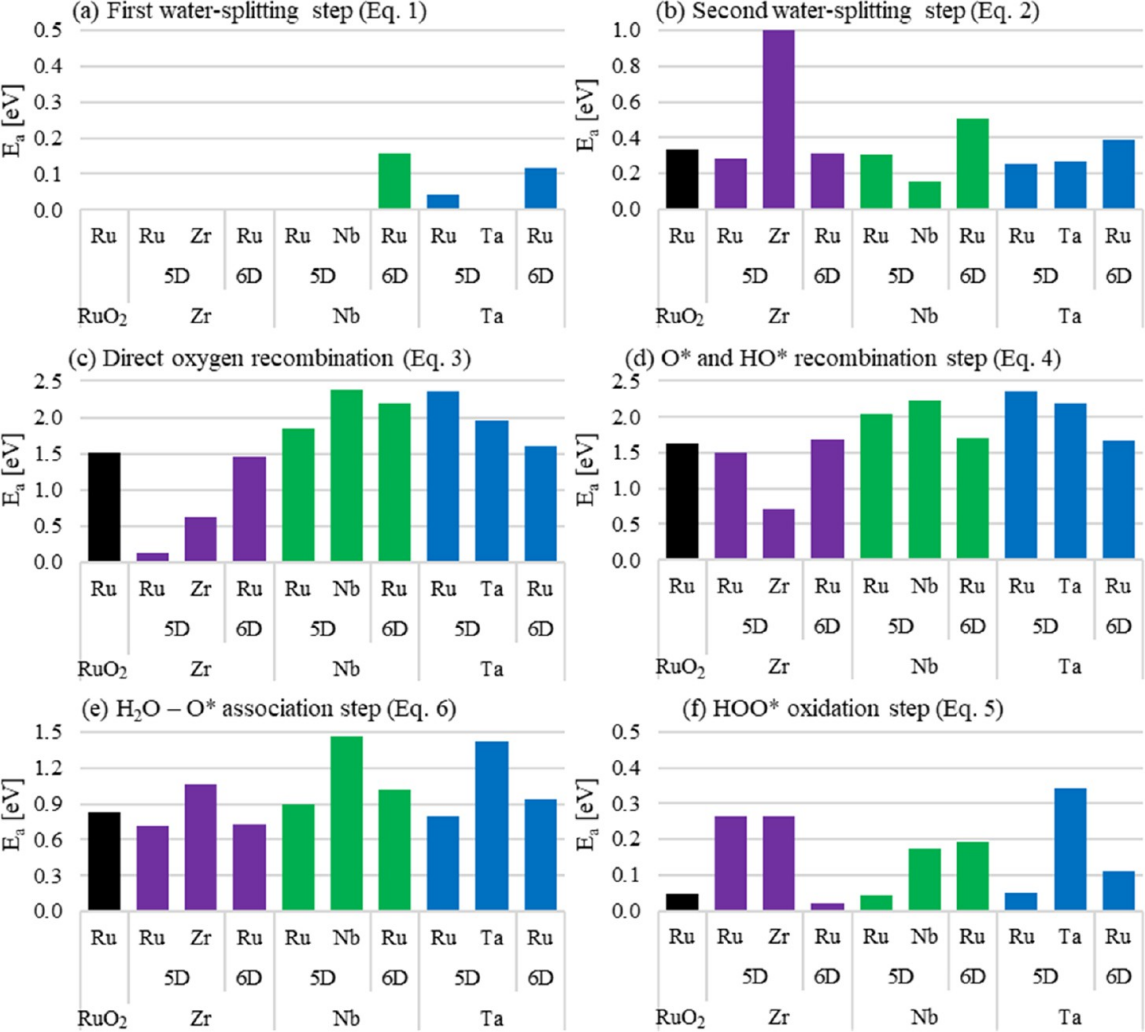
Comparative activation energies for (a) first and (b) second water splitting steps; (c) direct oxygen recombination mechanism; (d) first step of the oxygen and hydroxide recombination mechanism; and (e) first and (f) second stages of the water–oxygen associative mechanism on the 25% doped slabs. Black bars show the activation energy on the pristine RuO_2_ slab.

Direct oxygen recombination
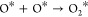
3Oxygen and hydroxide recombination steps
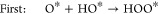
4
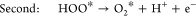
5Water–oxygen associative mechanism steps
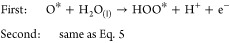
6First and second water oxidation steps (eqs 1 and 2) imply water adsorption and oxidation followed by hydroxide oxidation to form O* and 2(H^+^ + e^–^). After water oxidation steps, three different mechanisms of formation of molecular oxygen are evaluated: direct adsorbed oxygen recombination (eq 3), adsorbed oxygen and hydroxide recombination (eqs 4 and 5), and interfacial water and adsorbed oxygen association (eqs 6 and 5). First, we evaluate the adsorption energies (eV) of all surface species considered in eqs 1–7. In these analyses, we used an implicit solvation model with the water dielectric constant. Then, we evaluated the reaction and activation energies (eV) for each step using the climbing-image nudged elastic band (CI-NEB) method.^88−90^Figure 5 summarizes all activation energies.

#### 3.3.2 Water Splitting Steps

Relative activation energies for the first and second oxidation stages of the water molecule are shown in Figure 5a,b. The first oxidation step exhibits an almost exothermic nature in all cases with reaction energies ranging between 0.03 and −0.64 eV without major distinction between the results on Ru- or M-5 sites. In the same way, most of the different sites show barrierless profiles or very low activation energies 0.16 and 0.12 eV for the Ru site on Nb-25%-6D and Ta-25%-6D slabs, respectively. On the other hand, the hydroxide oxidation step shows an overall relatively low barrier on all systems, except for Zr. Zr shows low activation energies for the Ru sites on both the 25%-5 and 6D slabs; however, the activation energy on the Zr-5D site exceeds more than three times the activation energy on the pristine RuO_2_ surface (1.05 and 0.34 eV, respectively).

#### 3.3.3 Oxygen Evolution Steps

##### (a) Direct Oxygen Recombination

In this mechanism (eq 3, Figure 5c), two water molecules undergo complete oxidation (eqs 1 and 2) to form two O* species *on adjacent active sites*, followed by their combination to form O_2_* and its consecutive detachment from the surface to form gaseous oxygen. When evaluated on the pristine RuO_2_ surface, the reaction energy is very unfavorable. However, an interesting thermodynamic and kinetic behavior is observed when the surface is doped with the different species. As found in Figure 5c, Zr substitution offers an overall exceptional low activation energy down more than 90% (0.13 eV) over Ru-5 sites on Zr-25-5D. However, this behavior is highly dependent on the preference of Zr on the penta- or hexa-coordinate sites, as the activation energy on the Zr-25%-6D slab is 1.46 eV, almost as high as in the original RuO_2_ slab. Note that this mechanism requires O adsorbed in two adjacent sites, which also reduces the chances of finding the favorable active sites. In addition, these results must be related to the stability of the respective sites. As shown later, Zr may be slightly easier than Ru to dissolve in aqueous solution; therefore, if Zr is on the surface, the easiness for dissolution would reconstruct the surface to lower substituent concentration conditions. These results reinforce the fact that each site type, i.e., the penta- and hexa-coordinate sites, will play a different role regarding surface activity and stability. On the other hand, Nb and Ta show higher activation than the original Ru oxide energies for all active sites evaluated.

##### (b) Oxygen and Hydroxide Recombination

This is a two-step reaction (eqs 4 and 5, Figure 5d), that considers the combination of an adsorbed oxygen with a partially oxidized hydroxide molecule to form an adsorbed hydroperoxyl group (HOO*). This species suffers a subsequent oxidation to form O_2_* that finally detaches in gaseous form. The first step is summarized in Figure 5d and is the least favorable of all evaluated reactions in this work. On the pristine RuO_2_, it shows an activation energy of 1.64 eV, which hardly improves in the presence of the dopant species. Ru-5 sites in the presence of Zr show considerably better performances on the Zr-5D sites. The activation energies for this reaction in the presence of Zr are 1.49 eV on Ru-5 and 0.72 eV on Zr-5D from the 25%-5D, in contrast with the 1.69 eV required on Ru-5 from the 25%-6D slab. Additionally, Zr-5D sites show a reduction of around 64% of the energy required to trigger the reaction (0.58 eV), while Ru sites still exhibit an activation energy close to the one in the RuO_2_ sites (1.47 eV). Despite these apparent advantages, the overall step is unfavorable and the Zr dissolution issue is another factor against this step. In summary, even though there is a significant improvement given by the effect of Zr on the surface, the overall energetics of this first step of mechanism 2 are substantially unfavorable so the actual probability for this reaction step to occur is considerably low. The second step (HOO* oxidation) is the same as the one evaluated for the associative mechanism, and it is discussed below.

##### (c) Associative Mechanism

In this mechanism (eqs 6 and 5, Figure 5e,f), a free interfacial water molecule interacts with an adsorbed oxygen and a bridging oxygen (O_B_) to form a hydroperoxyl group (HOO*), which then follows the oxidation mechanism reacting with the surface oxygen to form O_2_. The first reaction step has an activation energy close to 0.80 eV when evaluated on the pristine RuO_2_ surface. Among the various substituents, Zr and Ta show a slight improvement in the energetics over the Ru-5 sites. Contrary to what was found in the recombination step (b), Zr-5D sites exhibit higher activation energies with 1.06 eV from the 25%-5D slab. Ta follows a similar trend but shows less favorable energies.

The hydroperoxyl oxidation is the second step of this mechanism; this reaction is highly favorable on the original RuO_2_ surface, where the activation energy is almost negligible, as shown in Figure 5f. Even though different substituents exhibit overall higher activation energies, they are not significantly higher to worsen the reaction. Hence, from this analysis, we can conclude that the hydroperoxyl formation is the complex limiting step for this mechanism to occur, in contrast with the fast and almost spontaneous oxidation step to form the molecular oxygen species.

In summary, this theoretical calculation of activation and reaction energies shows that, from the substituent species evaluated, Zr might offer some potential advantage in the oxygen recombination step. However, its effect is highly dependent on the dopant site type and concentration, and distribution of adsorbed species. Interestingly, Zr makes the adsorbed oxygen recombination more favorable compared to the original oxide as well as to the Ti substituent shown in our previous analysis.^44^ From these results, we observe an important contrast between the energetics of the O_2_ evolution mechanism given by eq 3 and the one given by eqs 4 and 5, where all Ru-5C sites in the presence of the Zr-5D substituent exhibit lower activation energies than the original RuO_2_ sites and the ones with the presence of other substituents. The occurrence of the direct oxygen recombination mechanism requires water to go through complete oxidation in order to get oxygen adsorbed on the surface active sites (usually as O_CUS_). Thus, the presence of Zr might require a combination of at least two mechanisms that drive the OER reactions, instead of the occurrence of the only one predominant proposed in our previous work.^14,44^ Moreover, this observation on the role of Zr may be a manifestation of the formation of oxygen vacancies, which has been suggested as a consequence of aliovalent doping that could also affect the OER mechanisms.^140^ We come back to this analysis after presenting the dissolution results, which demonstrate that both reactions, OER and metal corrosion, compete and cannot be analyzed separately.

Furthermore, the applied potential effect is added calculating the free energy profiles for the reactions involving one electron transfer (eqs 1, 2, 4, and 5) using the Norskov’s approach,^13,49,141,142^ which allows for calculating the stability of reaction intermediates of electrochemical processes based on electronic-structure calculations. Although recent reports^143,144^ have suggested that charges induced by applied fields may impact the energetics of electrocatalytic reactions, these effects are more likely to be large in 2D materials and less in 3D structures. Based on the results from Figure 5, direct oxygen recombination is not expected due to the large activation energies predicted from our calculations. For this reason, in the following analysis, the final O_2_ product is proposed to be formed by decomposition of the HOO* intermediate with the reaction taking place at the penta-coordinated sites (eqs 1, 2, and 5). From this approach, the free energy difference at each step is summarized as follows (full derivation in refs (13,142))
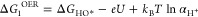
7
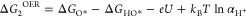
8
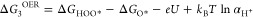
9
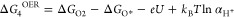
10where −*eU* includes the effect of a potential bias on all states involving one electron in the electrode, *U* being the electrode potential relative to the standard hydrogen electrode, and *k*_B_*T* ln α_H_^+^ allows for the correction of the free energy of H^+^ ions at pH different from zero, with ln α_H_^+^ = ln[H^+^]^−1^ = ln 10*pH. Figure S12 summarizes the results of these calculations for the pristine RuO_2_-(110) surface at pH ∼ 0. It is concluded that an *overpotential of 0.82 V* above the theoretical 1.23 V is needed to have every step exhibiting an exothermic nature. The effect of substituent species Zr, Nb, and Ta shown in Figures S13 and S14 for Ru_1–*x*_M_*x*_O_2_-(110) 25%-5D, and 25%-6D surfaces, respectively, suggest that the presence of hexa-coordinated substituents *reduces the overpotential needed* for the reactions occurring on Ru-5 sites at the 25%-6D coverage compared to the pristine surface. Moreover, Nb and Ta exhibit a highly favorable effect on the reduction of the overpotential needed for every step becoming downhill (0.20 V at 25%-5D and 0.09 V at 25%-6D, respectively, for Ta; and 0.14 V at 25%-6D for Nb). However, changes in the potential are known to be dependent to some degree on changes in the surface composition.^145^

#### 3.3.4 Activity descriptors and OER Overpotential

The reactivity of adsorbed oxygen is generally used as a key descriptor for oxidation chemistries as the OER.^146^ To evaluate the activity descriptors from our surfaces, we calculated the energy needed to deprotonate an adsorbed OH* and form adsorbed O*. This descriptor Δ*G*_O*_ – Δ*G*_OH*_ [eV] is an indicative of the reactivity of the oxygen atom due to the nature of the adsorption site. In this sense, it is not a characteristic only of the oxygen nature, but it is due to its interaction with the metal active site, giving an indirect measure of the surface activity. The results are summarized in Table S8.

**Figure 6:**
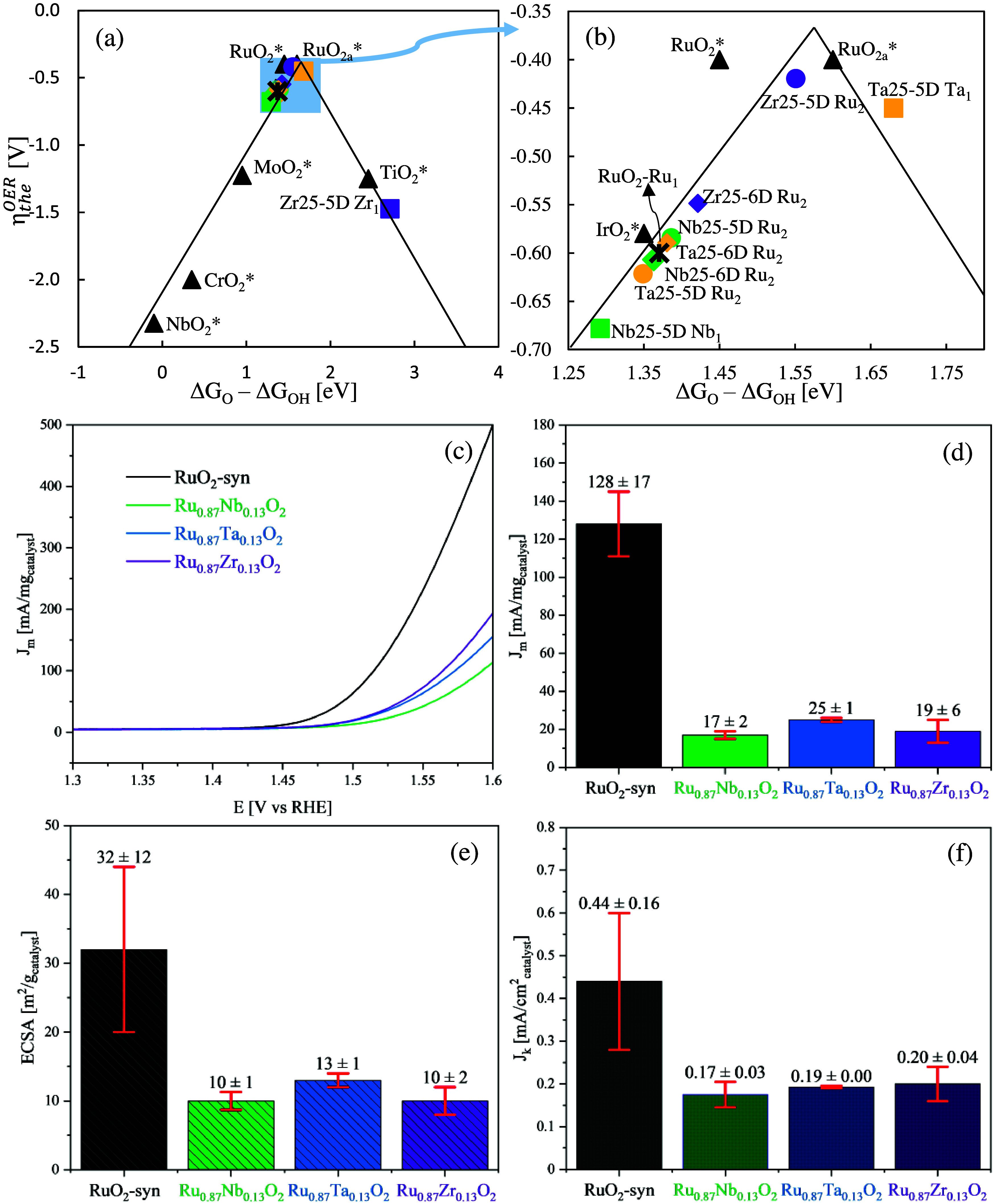
Theoretical overpotential vs activity descriptor indicates the activity trend compared with previous reports from ref (141) (black triangles). (a) Activity descriptor obtained from the favorable electrochemical reactions on all adsorption sites evaluated, (b) Inset from the blue region in panel (a). Geometric representation: circles, Ru-5 sites from 25%-5D slabs; squares, M-5 sites from 25%-5D slabs; rhombus, Ru-5 sites from 25%-6D slabs. Color code: purple, Zr; green, Nb; blue, Ta. Initial OER activity current in the oxygen evolution reaction (OER) voltage region for RuO_2_-syn, Ru_0.87_Nb_0.13_O_2_, Ru_0.87_Ta_0.13_O_2_, and Ru_0.87_Zr_0.13_O_2_ tested in Ar-purged 0.1 M HClO_4_ under rotation at 2500 rpm, (c) current from linear sweep voltammetry, normalized to catalyst mass, (d) comparison of OER mass activities at 1.51 V_RHE_, (e) comparison of electrochemical surface area (ECSA), and (f) comparison of OER specific activities at 1.51 V_RHE_ current from linear sweep voltammetry, normalized to the electrochemical surface area.

It is found that the deprotonation energy is a strong function of substituent coverage and active site type for each species. Considering the deprotonation energy on Ru-5C sites for the evaluated Ru_1–*x*_M_*x*_O_2_-5D-(110) surface concentrations, the highest deprotonation energy is obtained from the active sites within the Zr-substituted surface (1.55 eV), followed by the Nb-substituted surface (1.39 eV), while the original RuO_2_ surface exhibits a deprotonation energy of 1.37 eV and finally the Ta-substituted surface (1.35 eV). Furthermore, Zr substitution affects the deprotonation energy the most for both the 5D- and 6D-substituted surfaces, while the range of values in the presence of Nb and Ta remains closer to the deprotonation energy obtained from the original RuO_2_ surface, as shown in Table S8. Kinetic factors are also influenced by external potentials, included in our calculations for each active site using a previously reported relationship.^147^ Moreover, we use the Δ*G*_O*_ – Δ*G*_OH*_ activity descriptor to calculate the theoretical overpotential from DFT calculations from the expression^147^
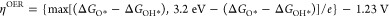
By convention, the negative of this theoretical overpotential (i.e., −η^OER^) is tabulated and plotted as shown in Table S8 and Figure 6a,b. The blue region in Figure 6a shows that most of the active sites evaluated lie close to the volcano top region, which indicates a good theoretical relationship between the overall low overpotential η^OER^ ([V]) and low deprotonation energy (Δ*G*_O*_ – Δ*G*_OH*_ [eV]), while the specific case of Zr-25%-5D Zr-5_1_, exhibits the highest overpotential. Figure 6b shows the low-overpotential region in more detail. The Ru-5 sites on Zr at 25%-5D surfaces exhibit overall best performance when compared to the previous analysis regarding surface activity. Overall, all sites with negative overpotential values higher than ∼−0.55 V (upper region in Figure 6b) have a potentially better performance than the active sites from the original oxide and the Ti-doped surfaces reported in our previous work.^44^ So, Ru-5 sites from Zr at 25% both 5D and 6D surfaces show a potential improvement, while the effects of Nb and Ta are highly dependent on the concentration and active site type.

#### 3.3.5 Experimental Analysis of the Effect of Metal Substituents on the OER Activity and Mechanism

We evaluated the initial surface of thin films of RuO_2_-syn, Ru_0.87_Nb_0.13_O_2_, Ru_0.87_Ta_0.13_O_2_, and Ru_0.87_Zr_0.13_O_2_ using cyclic voltammetry (CV). The CVs (Figure S15) showed very similar voltammetric features for all samples, and no distinct features were observed. The presence of niobium, tantalum, or zirconium within the RuO_2_ structure did not promote any new oxidation/reduction process in comparison to unsubstituted RuO_2_. Within the CVs, lower currents are observed with the incorporation of different metals, consistent with the calculated electrochemical active surface areas (ECSAs) for Ru_0.87_Nb_0.13_O_2_, Ru_0.87_Ta_0.13_O_2_, and Ru_0.87_Zr_0.13_O_2_ (Figure 6e), which are lower when compared to the RuO_2_ ECSA. The ECSA values were determined from the double layer capacitance values; however, obtaining ECSA values from either double layer capacitance or pseudocapacitance measurements that have high level of agreement with other surface area measurements (e.g., BET) is complicated by the relative contribution of the subsurface charge from a RuO_2_ surface region that is hydrated,^148^ alterations of the degree of surface order and stoichiometry,^149^ and adsorption-related charges.^150^

The initial oxygen evolution reaction (OER) activity of the samples was assessed using linear scan voltammetry (LSV) at a scan rate of 20 mV/s in a three-electrode setup as described in the Methods section. The LSV, mass activity, ECSA, and specific activity results of RuO_2_-syn, Ru_0.87_Nb_0.13_O_2_, Ru_0.87_Ta_0.13_O_2_, and Ru_0.87_Zr_0.13_O_2_ samples are shown in Figure 6c–f. From the mass normalization of the LSV data, the OER mass activity of RuO_2_-syn (128 mA/mg_cat_) was significantly higher than that of Ru_0.87_Nb_0.13_O_2_, Ru_0.87_Ta_0.13_O_2_, and Ru_0.87_Zr_0.13_O_2_ catalysts, which had mass activities of 17, 25, and 19 mA/mg_cat_, respectively, which shows that from our experimental analysis, the incorporation of different metals results in lower mass activity. The OER specific activity, related to the effectiveness of the reaction per active site, was also significantly higher for RuO_2_-syn (0.44 mA/cm_cat_^2^) in comparison to 0.17, 0.19, and 0.20 mA/cm_cat_^2^ for Ru_0.87_Nb_0.13_O_2_, Ru_0.87_Ta_0.13_O_2_, and Ru_0.87_Zr_0.13_O_2_, respectively. The OER specific activity shows a clear decrease with the incorporation of different metals, but the Ru_1–*x*_M_*x*_O_2_ catalysts all show very similar OER specific activities to each other. The decrease in mass and specific activity by the incorporation of niobium, tantalum, and zirconium within the rutile phase can be associated with different factors. One factor is the change in the electron distribution around the Ru active sites, which as shown in previous sections leads to a change of binding energies of the oxo-containing intermediate species (O*, OH*, OOH*) in the OER process and/or the formation of these adsorbed species that can alter the oxygen evolution reaction kinetic barriers. From our calculations, the effect of the metal substituent is highly dependent on the site and substituent concentration. The reduction in activity observed in the experiments generally agrees with the calculated activation barriers shown in Figure 5. We note that the activation barriers of the Ru sites for the metal-substituted oxides are in most cases higher than those in the pristine surface, thus corresponding to lower reaction rates. The only case that shows a few lower barriers is some of Zr sites. However, as discussed in the following section, Zr sites in the studied configurations are also very prone to spontaneously dissolve, which brings about a surface reorganization that may result in reaction sites with lower activity. In addition, differences between the model and the experimental surface structures, substituent metal concentrations, and expressed surface sites may also affect the comparison between experimentally measured OER activities and calculated energy barriers; and additional studies are needed to further connect experiments and theory.

We note that the change of the electronic structure can also lead to a reduction of the electronic conductivity by increasing the amount of Nb–O, Ta–O, or Zr–O bonds. The addition of Nb, Ta, and Zr, which are all considered insulating in their stable oxide form, with a band gap of 3.4, ∼4, and 5 eV, respectively,^123,151,152^ can yield a less-conductive material and affect the OER activity due to a high ohmic resistivity.^4^ However, a previous study analyzed the electronic conductivity of IrO_2_–TiO_2_ at varying Ir–Ti ratios and showed a decrease in electronic conductivity with increasing Ti, but satisfactory electronic conductivities were obtained even at 60 mol_M_% of Ti.^153^ Considering the 12.5 atom % substitution ratio, the addition of Nb, Ta, and Zr may not significantly reduce electronic conductivity; however, further analysis of the effect on substituents on electronic conductivity is needed. The decrease in OER activity with the substituents may be also due to differences in the surface structures, surface metal concentrations, specific surface sites expressed, contributions of the additional phase present, and surface evolution upon exposure to OER potentials.

To probe reaction mechanisms from our experimental analysis, we prepared Tafel plots derived from linear sweep polarization curves between 1.49 and 1.52 V vs RHE (Figure S16) and determined average Tafel slopes of 74, 75, 70, and 87 mV dec^–1^ for RuO_2_-syn, Ru_0.87_Nb_0.13_O_2_, Ru_0.87_Ta_0.13_O_2_, and Ru_0.87_Zr_0.13_O_2_, respectively. The Tafel slopes of the Ru_1–*x*_M_*x*_O_2_ catalysts are comparable within the error, which suggests that all catalysts follow similar reaction pathways for the synthesized materials. We note that Tafel slope values reported in the literature for RuO_2_ vary between different samples and can be dependent on experimental conditions, particle size, and crystallographic orientation of the materials.^154−156^ Tafel slope values ranging from 55 mV dec^–1^ for the (101) plane to 140 mV dec^–1^ for the (011) plane have been reported.^156^ Tafel slopes were also shown to decrease with decreasing average RuO_2_ particle size, indicating that the change in the particle size affects the electron transfer rate for oxygen evolution.^155^ Our experimentally determined Tafel slopes are in good agreement with previously reported RuO_2_ values at low overpotentials (e.g., <∼1.52 V).^39,157^ The direct connection of our experimental Tafel slopes to a specific theoretical mechanistic pathway is complicated by the contributions of the adsorbed species, rearrangements of reaction sites, and influence of the electrolyte, and the symmetry factor as described in our prior study.^158^

### 3.4 Metal Dissolution and Electrochemical Stability of Zr-Substituted RuO_2_

#### 3.4.1 Computational Analysis of the Dissolution of Metal Atoms from the Mixed Oxide

To further investigate the correlation between the OER activity and the corrosion and dissolution of metal oxides, constrained AIMD (c-AIMD) simulations were performed to evaluate the dissolution of Ru and M surface atoms from the pristine RuO_2_(110) surface and Ru_0.75_Zr_0.25_O_2_-5D and 6D surfaces. Zr was selected as a metal representative substituent. In this analysis, the dissolution of penta- and hexa-coordinated metals on the surfaces is evaluated using the thermodynamic integration within the slow-growth approach as implemented in the Blue Moon ensemble method in VASP.^159−163^ This approach allows us to evaluate the activation barriers involved in the cation dissolution, starting from an initial configuration on the surface and following the free energy profile until it forms a stable and fully dissolved species in the aqueous media. As a result, we identify important intermediate steps along the dissolution path.^164−166^ The electrolyte phase (not shown in the figures below) contains 27 water molecules (approximately two monolayers on top of the surface). Numbered states along the free energy profile for metal dissolution start from the initial surface configuration (I) to the fully dissolved, stable compound in the electrolyte phase (V or VI, depending on the case). Each penta-coordinated metal (both Ru and Zr) starts from configuration I, where it is bonded to a subsurface oxygen located right below it, shares four bonds with surface oxygens, which comprise three-coordinated oxygen atoms (O_t_) and an oxygen coordinatively unsaturated site (O_CUS_) adsorbed to it (for the fully oxidized case). For the case of the hexa-coordinated metals, the initial surface configuration has Ru sharing bonds with two subsurface oxygen atoms, two surface O_t_ atoms, and two oxygen bridges (O_B_ atoms).

**Figure 7:**
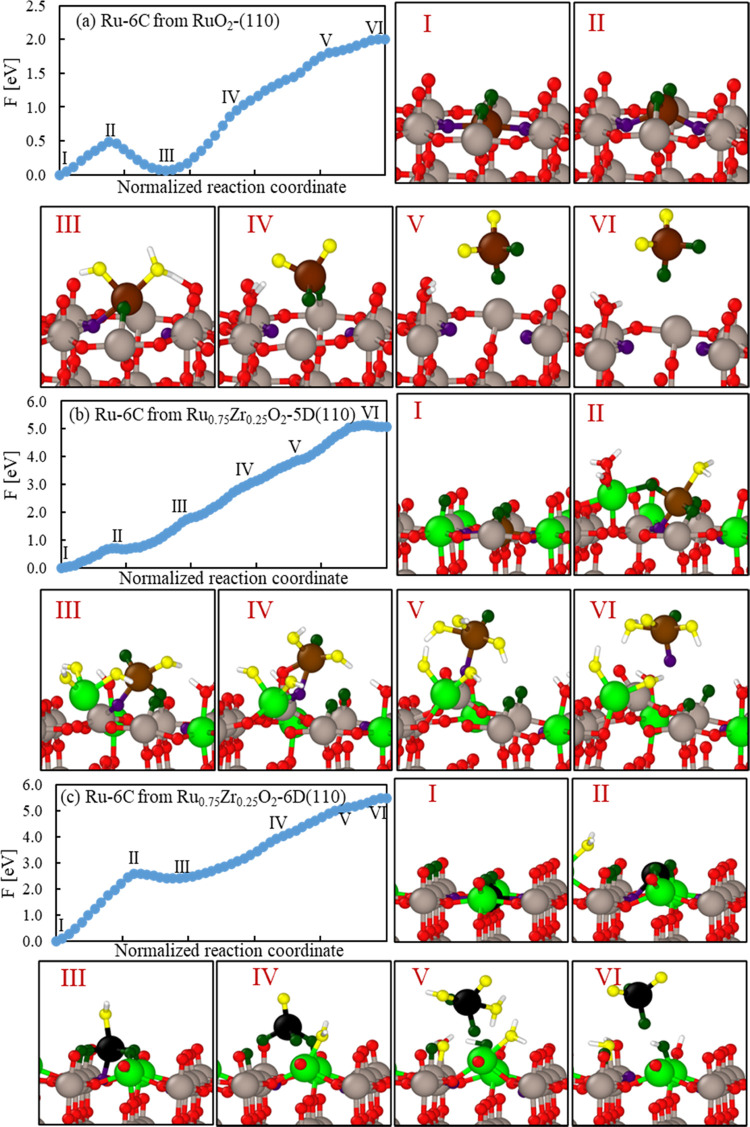
Free energy profile of ruthenium hexa- (6C) coordinated dissolution from (a) pristine RuO_2_-(110), (b) Ru_0.75_Zr_0.25_O_2_-5D(110), and (c) Ru_0.75_Zr_0.25_O_2_-6D(110) surfaces, with the intermediate events along the dissolution path labeled from I (initial state) to VI (fully dissolved species). See the text for explanation of the intermediate events. Color code: Ru, silver; Zr, light green; dissolving Ru, black/brown; O, red; H, white; Ot, purple; O_CUS_, dark green; and O from free water molecule, yellow. Water molecules in the electrolyte are not shown for clarity.

As illustrated in Figure 7, our analysis of Ru dissolution pathways shows that the reaction pathways and energy barriers are highly dependent on the site. *Ru-6C dissolution* from the RuO_2_-(110) pristine surface exhibits a rather peculiar free energy profile as shown in Figure 7a. The uphill free energy profile from state I to II indicates the requirement of ∼0.48 eV for the dissolving Ru to break the two bonds with the subsurface oxygens (much easier than for Ru-5C, as explained below). However, the intermediate coordination is stabilized by the adsorption of two free water molecules, and in the process of adsorption and first oxidation of one of the molecules, the system reaches an energetic stable configuration represented by state III. In that configuration, the dissolving Ru-6C atom keeps one OH group adsorbed (product of water oxidation) and one adsorbed water molecule. An energy intake of ∼1 eV is required for the Ru atom to move from III to IV. During this step, Ru attracts the first O_B_ from the neighboring Ru-6C, leaving this 6C site in a 5C configuration prompted to act as an active site due to its original coordination imbalance, as well as attracting a second O_B_ atom, generating two new Ru-5C sites due to the coordination imbalance explained above. Along this path, full oxidation of the adsorbed species occurs, which defines a dissolving RuO_4_ adsorbed on the neighbor Ru-6C site. Moreover, it can be observed (in intermediate events in Figure 7a) how one of the O_CUS_ on the surface helps to promote the oxidation step of the adsorbed water molecules on the dissolving Ru by adsorbing H to form an adsorbed H_2_O* species. Finally, the transition from state IV to states V and VI is regarded to the solvation energy of the dissolving complex and its reconfiguration into the aqueous media.

When Zr is present on the surface, Figure 7b, Ru-6C dissolution from the Ru_0.75_Zr_0.25_O_2_-5D(110) slab shows a rather interesting dynamic interaction between the Ru-6C and Zr-5D neighbor atoms, i.e., both Ru and Zr sharing the same O_t_ atom. The energy profile shows that ∼0.70 eV is required to reach state II. However, in state II, one can see that the dissolution starts to happen *from a metal cluster* instead of a single metal atom where the *neighbor Zr-5D atom is dissolving along with the Ru-6C metal*. Zooming in, the dissolving Ru-6C has broken one of its O_t_ bonds and adsorbed a free water molecule. Interestingly, the neighbor Zr-5D atom has broken four of its main surface bonds, adsorbing and partially oxidizing two water molecules, and additionally bonding with an O_B_ atom. With an additional ∼1.09 eV, the dissolving system reaches state III, where the dissolving Ru-6C breaks one of its O_B_ bonds and partially oxidizes the adsorbed water molecule. Moreover, at this point, Zr-5D has broken all its structural bonds with the surface and remains attached only to the dissolving metal via a partially oxidized free water molecule adsorbed, while Ru-6C remains attached to the surface via two bonds: its remaining O_B_ and O_t_ bonds. Additional ∼1.19 eV take the system to state IV, where the dissolving Ru-6C has broken its last O_B_ bond and remains attached to the neighbor Ru-5C atom via the O_t_ and O_CUS_ bonds, as it attracts the shared HO* from Zr-5D. This interaction pulls the Ru-5C metal into dissolution dynamics as it can be seen in the intermediate image that this atom has already broken its subsurface O bond and starts to go away from the surface. Interestingly, at this point, the Zr-5D atom involved in the initial dissolution cluster, after losing the HO* group to Ru-6C, reattaches to some of its structural O_t_ bonds, sharing them with both the Ru-6C and Ru-5C atoms that undergo the dissolution process. An energy intake of ∼1 eV will be necessary for the system to reach state V, where the dissolving cluster starts to separate as the originally dissolving Ru-6C atom breaks the O_CUS_ bond and adsorbs a free water molecule. Finally, the dissolving system requires additional ∼1.15 eV to break the dissolving cluster into the dissolved Ru-6C metal, which forms RuO_3_(H_2_O)(OH) dissolved species, and the Ru-5C-Zr-5D cluster reattached to the surface, with vacant metal and oxygen sites due to the surface reconstruction observed. Thus, while Figure 7a shows a “clean” Ru-6C dissolution from the pristine surface, the role of Zr trying to impede Ru dissolution is clear from Figure 7b, that shows a much higher overall energy barrier, suggesting that Zr is indeed successful impeding Ru dissolution. Moreover, although the neighbor Zr atom itself also was at the verge of dissolution, it remained on the surface with significant surface reconstruction.

Next, we look at an equivalent Ru-6C site but Zr located in a different (6D) position, i.e., Ru-6C dissolution from the Ru_0.75_Zr_0.25_O_2_-6D(110) slab, summarized in Figure 7c. The free energy profile shows another difficult Ru dissolution. About ∼2.59 eV is required to start breaking three of the main Ru–O bonds from the initial state I until it reaches state II. At that point, the dissolution exhibits a slightly exothermic performance until it reaches state III, where the dissolving metal has stabilized its coordination by creating new bonds with two neighbors O_CUS_ and by adsorbing a free water molecule. Adding ∼1.41 eV allows the system to reach state IV, where the dissolving metal has attracted one of the O_CUS_ and one of the O_B_ from the surface. This event forms two new active sites on the surface, one typical vacant Ru-5C and one new type of Zr-5D site from the original Zr-6D site that has lost one of its O_B_ to the dissolving atom. This can be seen in the figure as a water molecule adsorbed on the Zr-D atom. Moreover, the dissolving metal fully oxidizes the adsorbed water molecule and remains attached to the surface by its last Ru–O_B_ bond. Continuing with the dissolution process, an energy intake of ∼1.20 eV allows the dissolving metal to break its last Ru–O_B_ bond to detach from the surface and adsorb, partially oxidizing a free water molecule while weakly attracting a second one, reaching state V. The figure for this state also shows that a free water molecule has adsorbed on the vacant Ru-5C site left by the dissolving compound. Eventually, the dissolving compound completely incorporates into the aqueous media as stable RuO_4_ after a final energy intake of ∼0.28 eV to reach state VI. Overall, Figure 7b,c informs us that Ru should be much more stable on surfaces with some Zr atoms.

**Figure 8:**
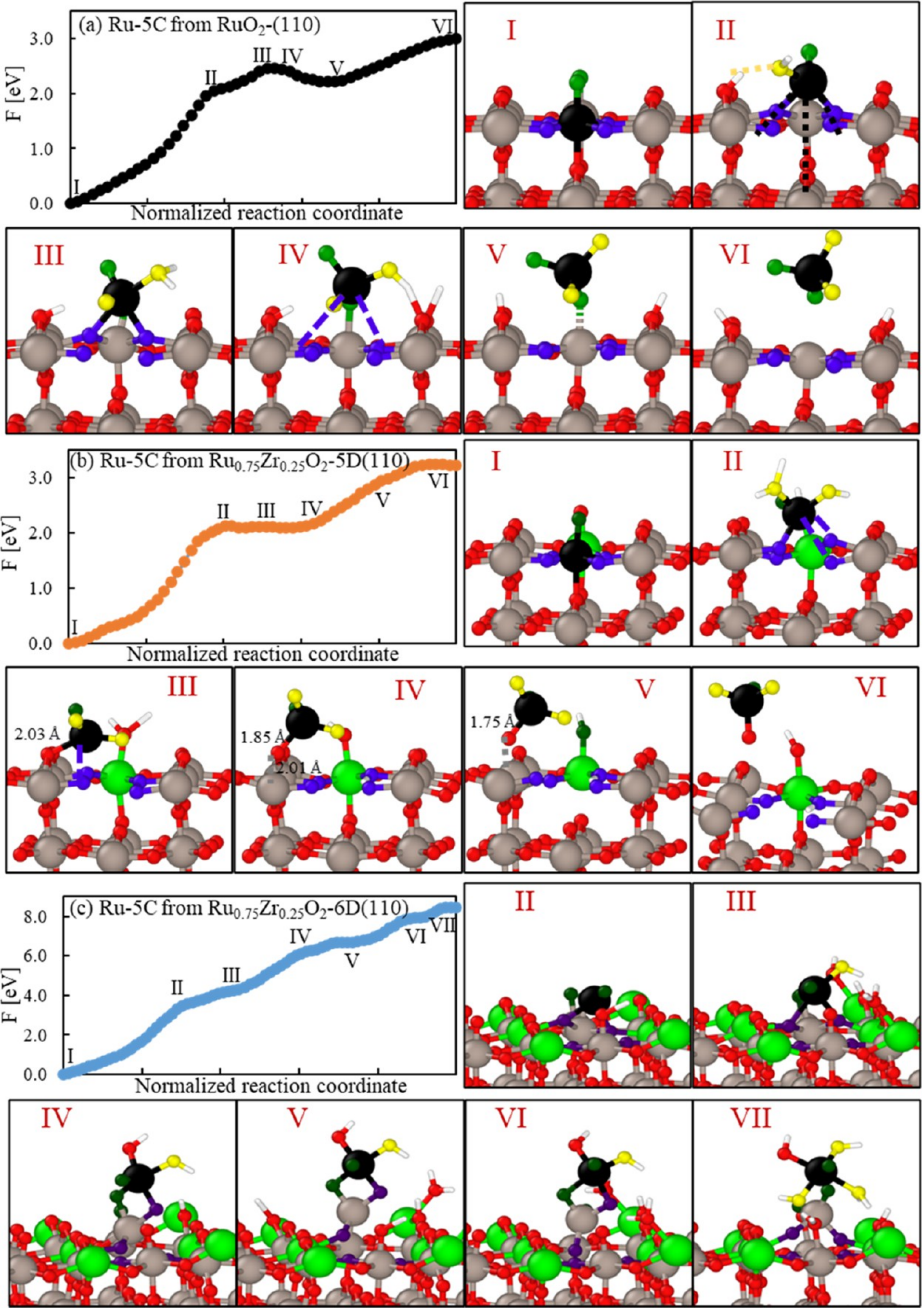
Free energy profile of ruthenium penta- (5C) coordinated dissolution from (a) pristine RuO_2_-(110), (b) Ru_0.75_Zr_0.25_O_2_-5D(110), and (c) Ru_0.75_Zr_0.25_O_2_-6D(110) surfaces, with the intermediate events along the dissolution path labeled from I (initial state) to VI (fully dissolved species), except for (c) where the initial step I (not shown) is the same as Figure 7c. See text for explanation of the intermediate events. Color code: Ru, silver; Zr, light green; dissolving Ru, black; O, red; H, white; Ot, purple; O_CUS_, dark green; O from free water molecule, yellow.

Figure 8a summarizes the free energy profile for the dissolution of ruthenium penta- (5C) from the pristine RuO_2_-(110) surface. It exhibits an initial energy cost of ∼2 eV to reach state II, where the dissolving Ru is bonded with only two of the surface oxygen atoms (O_t_) and adsorbs a free water molecule. An initial cost of ∼2 eV is needed for breaking the bond with the subsurface oxygen atoms and two bonds with two surface O_t_ atoms. This coordination is stabilized by the adsorption of a free water molecule that undergoes a first oxidation step, releasing a proton. Following the dissolution path, an additional energy intake of ∼0.45 eV takes the dissolving system to state III, where the initial adsorbed water molecule is fully oxidized and a second water molecule is adsorbed to the dissolving metal atom. Change to state IV is thermodynamically stable and occurs without any additional energy. In this state, the second adsorbed water molecule goes through a first oxidation step and the remaining H starts an interaction with the surface O_B_. Moreover, the dissolving Ru atom breaks its remaining bonds with the O_t_ atoms and attracts a neighboring O_CUS_. In state V, the dissolving compound stabilizes, reaching a lower-energy state compared to state IV, after the oxidation step occurring in the last *OH group adsorbed to form a RuO_4_* species adsorbed on a Ru-5C active site via Ru–O_CUS_ bonds. Finally, along the uphill energy profile shown between states V and VI, the RuO_4_ compound is desorbed, leaving a Ru-5C active site. The additional energy required to reach state VI (∼0.53 eV) represents the solvation energy related to the stable incorporation of the dissolved RuO_4_ into the aqueous media.

The complete free energy profile in Figure 8b is for a surface of Zr-substituted Ru_0.75_Zr_0.25_O_2_-5D. The initial activation barrier for Ru-5C dissolution remains almost unaltered compared to the barrier in the pristine RuO_2_ slab (∼2.53 vs ∼2.46 eV, respectively). An energy intake of ∼2.13 eV is needed to push the system from state I to state II, where the dissolving metal has already broken four of its main surface bonds, with only the last Ru–O_t_ bond keeping it bonded to the surface. Two free water molecules are readily adsorbed and one of them is rapidly oxidized. States III and IV represent an energetic stabilization where the dissolving compound fully oxidizes the attached water molecules and breaks its last surface bond to rapidly attract the closest O_B_ that becomes adsorbed. The oxidation of the attached water molecule occurs with participation of some of the neighbor O_CUS_ that attract some of the protons that become adsorbed (not all shown). Next, the dissolving species requires an energy surplus of ∼0.41 eV to reach state V from state IV, in which it finally attracts the O_B_ atom, breaking the second original bond, leaving the same *new kind* of Ru-5C sites observed with the Ru-5C dissolution on the pristine surface. Dissolved RuO_4_ is formed in state V, but it requires an additional ∼0.71 eV to fully incorporate and stabilize into the aqueous media. We highlight that while Ru dissolution occurs, one specific Zr-5D (not shown in figures) breaks some of its main surface bonds to attract and oxidize different water molecules. This is shown in SI to be due to a dynamic Zr preference to dissolve compared to Ru. Thus, Ru-5C dissolution in the presence of Zr-5 coordinated is as favorable as in the pristine surface.

A different profile is shown for Ru-5C dissolution from Ru_0.75_Zr_0.25_O_2_-6D (Figure 8c), where there is a high initial intake energy of ∼3.58 eV to reach state II. At this state, the dissolving Ru-5C atom has already broken three of its main bonds and started to strongly interact with a second Ru-5C atom. An additional energy intake of ∼0.65 eV allows the system to reach state III. In such a state, the dissolving atom has adsorbed and partially oxidized a free water molecule, while some others are adsorbed and oxidized on the surface by interaction with Zr atoms. Moreover, state IV is reached after an energy requirement of ∼1.98 eV, where the initially dissolving Ru-5C has adsorbed and partially oxidized a second free water molecule, breaking its bond with one of the remaining O_t_ atoms while attracting the other one from the surface. The O atoms shared with the second Ru-5C atom strongly attract it, inducing the second Ru-5C to break its bond with the subsurface oxygen attached to it, triggering its participation in forming a dissolving cluster compound by two metal atoms. From state IV to state V, the free energy profile exhibits an energy increment of ∼0.48 eV, which makes the second Ru-5C from the dissolving cluster break two of its Ru–Ot bonds as it bonds to the closest O_CUS_ from a third Ru-5C on the surface. After an energy intake of ∼1.22 eV, the system reaches state VI, where the dissolving cluster (Ru_2_O_5_(OH)_2_) is formed after the second Ru-5C in the cluster retains the last Ot and the O_CUS_ from the surface. However, the complete compound does not incorporate into the electrolyte; instead, the bottom part, i.e., the second metal and its O atoms remain in an intermediate interfacial solvation-type interaction with the surface and the vacancy formed by the dissolution of the whole complex. Hence, to finally induce the dissolving compound to incorporate into the aqueous media, the initially dissolving Ru-5C is pushed from the surface, which requires an additional ∼0.56 eV until it reaches state VII, where the dissolving cluster separates, and the second metal partially reincorporates to the surface. In doing so, the fully dissolved metal atom adsorbs and partially oxidizes two additional free water molecules, forming a RuO_5_H_5_ species stable up to the end of the simulation time. Overall, the events in Figure 8c are highly energy-demanding and unlikely to happen, which also confirms the role of Zr impeding Ru dissolution in this case.

We also determined the reaction pathways and energy Zr-5D and 6D from the Ru_0.75_Zr_0.25_O_2_-5D and -6D slabs (see Figure S17 and supporting text in the SI) A complete discussion of these reactions is included in SI. From these results, it is important to highlight that in the free energy profiles for both Zr-5d and 6D, two different energy range regions can be identified: the short energy range where the Zr atoms require low energy input to break the structural bonds, and the long energy range where, after breaking the surface bonds, the dissolving compound requires a higher energy input to incorporate into the electrolyte. This trend indicates that despite its easiness to break bonds with the subsurface, the energies for complete dissolution of Zr atoms are comparable to those of Ru atoms dissolving from the pristine surfaces.

From the analysis of the dissolution of Ru and Zr in various sites, we concluded that the predominance of Zr-5D increases the initial activation energy for dissolving Ru-6C by more than 40% compared to the original RuO_2_ and the prevalence of Zr-6D atoms on the surface induces considerably higher initial activation barriers for both Ru-5C and Ru-6C (up to 60 and 240% higher energy requirements, respectively). This would imply that the presence of Zr makes it more difficult for Ru to dissolve from these sites. However, Zr-5D and -6D dissolution is much easier based on the considerably lower initial activation energy (Figure S17). Thus, we can conclude that Zr dissolves first from surface sites, either reaching a lower “equilibrium” concentration (i.e., a minimum Zr concentration that is stable in this specific system) or leaving a surface with similar composition but different structural characteristics to a pristine one. Even though some positive effects may exist over Ru dissolution, this Zr dissolution effect should predominate. However, the prevalence for higher solvation energies compared to the initial activation energies for dissolution indicates that once Zr breaks its structural bonds, it may remain coordinated to the surface instead of fully incorporating to the aqueous media, which may also influence the surface activity and Ru stability.

#### 3.4.2 Experimental Analysis of Electrochemical OER Stability and Metal Dissolution

**Figure 9:**
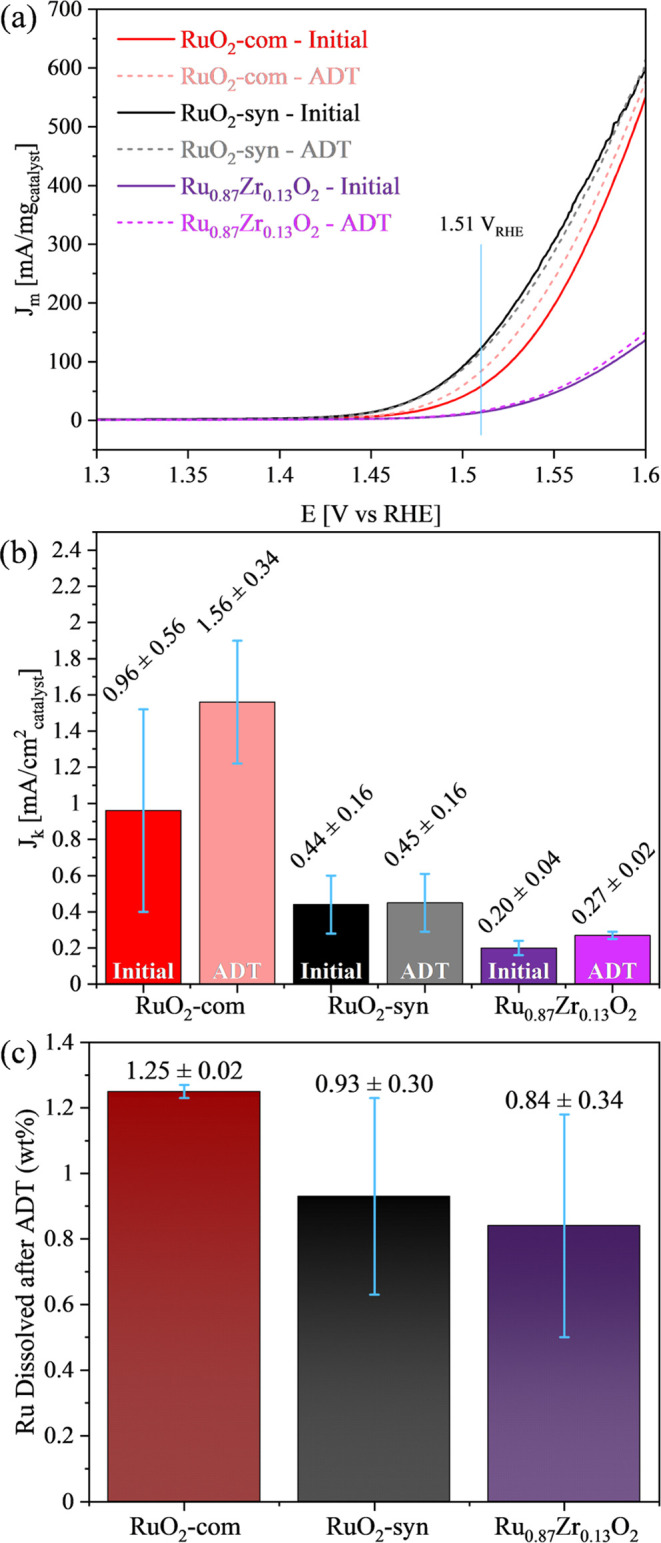
Comparison of rotating disk electrode (RDE) results and Ru dissolution after accelerated durability test (ADT) for RuO_2_-com, RuO_2_-syn, and Ru_0.87_Zr_0.13_O_2_ tested in Ar-purged 0.1 M HClO_4_ under rotation at 2500 rpm: (a) current from linear sweep voltammetry, normalized to catalyst mass, (b) OER specific activities evaluated from the current at 1.51 V_RHE_ from linear sweep voltammetry normalized to the electrochemical surface area, and (c) wt % of Ru dissolved after ADT.

To compare with our calculations, the electrochemical stability of our synthesized materials, RuO_2_-syn and Ru_0.87_Zr_0.13_O_2_, and a commercial RuO_2_ material (notated as RuO_2_-com) was evaluated using an accelerated durability test (ADT), used by our group and others,^24,167^ which consists of applying a constant potential (1.6 V_RHE_) for 13.5 h. The linear sweep voltammograms of the initial LSV and LSV after ADT are shown in Figure 9a. Figure 9a shows higher mass activities for RuO_2_-com and Zr-doped materials after ADT; however, a reduction was observed for RuO_2_-syn. OER mass activities obtained at 1.51 V_RHE_ from the initial LSV and ADT LSV are shown in Figure S18a. The changes in the OER mass activity from initial to after ADT result from several factors including changes to the ECSA, changes to the active site, and dissolution. Using the determined ECSA values (Figure S18b), we determined the specific activity of RuO_2_-com and Ru_0.87_Zr_0.13_O_2_ increased after ADT, while the specific activity of RuO_2_-syn is within the experimental error (Figure 9b). Our testing of ECSA of the catalysts (Figure S18b) showed initial and ADT ECSA values within the experimental error. From our experimental analysis of factors contributing to the mass activities, the higher initial mass activity of RuO_2_-syn versus RuO_2_-com and Ru_0.87_Zr_0.13_O_2_ is attributed due to its higher surface area (more active sites). On the other hand, RuO_2_-com, despite its lower ECSA, has a higher mass activity than Ru_0.87_Zr_0.13_O_2_ due to its higher specific activity (Figure 9b). After ADT, RuO_2_-com, despite decreasing about 30% of its ECSA (lower stability), showed a higher mass activity due to an increase in specific activity, which is associated with changes to the surface structure. The slight reduction of mass activity of RuO_2_-syn after ADT can be attributed to the combination of a similar specific activity and less active sites. After ADT, Ru_0.87_Zr_0.13_O_2_ retains its active surface area (high stability) and increases its specific activity (by 35%), which adds to an overall higher mass activity than initially. Combining what we learned from experiments and simulations, we suggest that the high retention of mass catalytic activity in the presence of Zr may be due to two reasons: (1) Ru active sites are protected from dissolution because of the Zr sites and (2) some Zr and Ru dissolution may facilitate surface restructuring and creation of alternative active sites, which will be addressed in the future work.

To correlate the OER stability with dissolution, we collected the electrolyte after our ADT and determined the amount of Ru and Zr ions dissolved via ICP-MS. The evaluation of the stability is affected by the RDE film differences, changes in the surface structure of the catalyst during the RDE experiments, and subsequent surface reconstruction, which all play a critical role in correlating OER activity and stability. Correlating ICP-MS with RDE can provide metal dissolution rates and/or profiles of catalysts^50,168^ but can also yield inconclusive correlation between stability and activity,^50^ in part due to the challenges noted above. For our study, the weight % of Ru dissolved during the ADT for different catalysts is shown in Figure 9c. RuO_2_-com had the largest amount of dissolved Ru when compared to the synthesized RuO_2_-syn and Ru_0.87_Zr_0.13_O_2_. For RuO_2_-com, the increase in the OER mass activity after ADT and the high amount of Ru dissolved suggest that the dissolved ions alter the surface and result in more active surface sites, which may explain the high mass activity retention of RuO_2_-com under these test conditions. From our theoretical calculations, it is possible that a Ru-6C site dissolves and leaves two new Ru-5C active sites with better catalytic activity. Our analysis supports that the extended time under OER conditions over the ADT protocol results in metal dissolution and an increase in the specific activity for Ru_0.87_Zr_0.13_O_2_. In addition, RuO_2_-syn and Ru_0.87_Zr_0.13_O_2_ show lower Ru dissolution rates compared to RuO_2_-com.

The comparison of Zr dissolution from Ru_0.87_Zr_0.13_O_2_ and Ti dissolution from Ru_0.87_Ti_0.13_O_2_ from our prior work^167^ shows that Zr dissolution of 4.32 ± 3.89 wt % (Table S9) from Ru_0.87_Zr_0.13_O_2_ is lower than Ti dissolved (10.6 ± 1.6 wt %) from the Ru_0.87_Ti_0.13_O_2_ samples that may indicate that Zr substituted into RuO_2_ may be more stable than Ti substituted into RuO_2_ under these testing conditions. The lower amount of Zr dissolved when compared to Ti agrees with a previous study where anodic oxide films on zirconium were found to be more stable than those formed on titanium in nitric acid solutions.^169^ The experimental Zr dissolution aligns with the theoretical calculations of activation barriers for Zr dissolution having lower overall activation barriers than Ru dissolution. Furthermore, it is observed from theoretical calculations that as Zr is dissolved, a reconstructed surface left behind new active sites, and there is a complex structural relationship among the remaining metal atoms on the surface. Moreover, Zr dissolution energies suggest that Zr tends to leave its structural sites on the surface but requires a higher energy input to fully incorporate into the aqueous media. This behavior may suggest that Zr–surface interaction prevails, affecting both activity and stability of the surface sites. This could also explain the large deviations of Ru dissolution (within error of RuO_2_-syn) in the Ru_0.87_Zr_0.13_O_2_ samples. We also note that the theoretical model of Zr dissolution considers a dissolution from a single site but does not quantify what would happen to the entire surface that could involve surface and subsurface Zr at different concentrations. Considering the comparatively observed low energy barriers for Zr dissolution and experimentally observed Zr dissolution from Zr-substituted RuO_2_, we consider that the Zr dissolution process for a substituted Zr ion from a RuO_2_ lattice may be much different than Zr dissolution from anodically formed zirconium oxide films that were highly stable.^169^ The effect of the initial metal dissolution on surface reconstruction and its effect on subsequent metal dissolution energy barriers and dissolution rates remains to be determined. We note a prior study of RuO_2_ particles and well-defined surfaces showed the amount of Ru corrosion is generally much more pronounced during the first stability test but is lowered during the second stability test, which was attributed to different amounts of surface defects and surface reconstruction.^50^

## 4 Conclusions

From our combined computational and experimental study of Zr, Nb, and Ta substitution into rutile RuO_2_, we find that the metal substitutes significantly alter the atomic and electronic structure, OER activity, and metal dissolution. The effects on the atomic and electronic structure are highly dependent on the specific metal substituent, concentration, and substitution site. The Zr, Nb, and Ta substituents alter the rutile lattice parameters, with anisotropic shifts in “*a*” and “*c*” lattice parameters, which depend on the substituent and concentration. From the experiments using our hydrothermal synthesis route, X-ray diffraction and Rietveld fitting analysis of the materials with Zr and Ta substitution at 12.5 atom % showed that the materials exhibited a majority phase with the substituent metal and a smaller percentage of a separate RuO_2_ phase. Nb substitution at 12.5 atom % showed two clearly distinct phases were present. The experimental Zr-, Nb-, and Ta-substituted materials showed higher substituent concentrations compared to the bulk, which may result from differences in the hydrolysis and condensation reaction rates, surface segregation during thermal treatment, and/or removal of volatile RuO_4_ during thermal treatment. From computations, the electronic structure is altered by the substituents, and computations show changes in the surface density of states and regions of electron density depletion and accumulation that depend on the site, substituent, and concentration. Consistent with our calculations, X-ray photoelectron spectroscopy experiments show shifts in the binding energies of O-2s peaks for all substituents and show shifts in O-2p and Ru-4d peaks for Zr substitution.

From computational analysis of multiple OER mechanistic pathways, the Zr, Nb, and Ta substituents alter the activation barriers of the OER reaction steps. Computations of the OER at rutile (110) surfaces show that the associated mechanism is the most probable single site reaction pathway at RuO_2_ and Nb- and Ta-substituted RuO_2_ surfaces; however, Zr substitution may result in a different OER mechanistic pathway as Zr substitution also favors the direct oxygen recombination and slightly improves the activation energy for the oxygen–OH recombination reaction. From the experiments, Zr, Nb, and Ta substitution at 12.5 atom % results in lower OER mass and specific activity relative to RuO_2_, which agrees with computations of the average of all sites. Zr and Ta substitution at specific sites results in higher theoretical OER activity compared with RuO_2_. However, this specific distribution of sites might not be always exposed. Computational analysis of metal dissolution shows that the reaction pathway and energy barriers are highly dependent on the site of the dissolving ion, and the dissolution process involves cooperative interactions between multiple surface sites and water from the electrolyte. From experiments, comparable amounts of Zr and Ru are dissolved into the electrolyte, which is in line with our calculations. Theoretical evaluation of the dissolution pathways clearly shows the role of a substituent like Zr on impeding Ru dissolution, while the surface is actively reconstructed. On the other hand, substituents like Zr may be also relatively easy to be dissolved, while in the process, contributing to surface evolution and formation of new active sites. Activity measurements after accelerated dissolution tests point to high retention of mass catalytic activity in the presence of Zr, which based on our combined approach may be attributed to two reasons: (i) Ru active sites are protected from dissolution because of the Zr sites, and (ii) some Zr and Ru dissolution may facilitate surface restructuring and creation of alternative active sites.

Indeed, our results support that dissolution of both active and supporting metals plays a critical role in changing the surface and influencing the OER activity and stability, and metal dissolution and OER activity are highly dependent on the substituent, concentration, and site. Further understanding of factors influencing activity and stability of OER electrocatalysts contributes to developing catalysts with lower cost, improved activity, and enhanced durability. We consider the importance of surface evolution/reorganization, metal dissolution, and deviations from ideal surfaces as important to further elucidate the complex relationships between OER activity and stability. In addition, studies of metal-substituted RuO_2_ OER electrocatalysts, including inline time and potential resolved dissolution profiles, multiple stability tests, stability tests using different voltages, and testing in membrane electrode assemblies, and characterization of surface structure during operation are needed to further understand the interrelationships between the surface structure, activity, and dissolution.

## Supporting Information Available

EDS and XPS analysis of the oxides (Table S1), XRD data (Figures S1, S2 and Table S2), crystallographic parameters (Table S3), atomic positions and occupancies (Table S4), DOS (Figures S3–S7), Ru-4d band and O-2p band centers for various oxides (Table S5), XPS data (Figures S8–S10 and Tables S6, S7), radial distribution functions (Figure S11), effects of applied potential on the potential energy profiles (Figures S12–S14), activity descriptor and overpotential (Table S8), cyclic voltammetry (Figure S15), Tafel plots (Figure S16), potential energy profiles for metal dissolution (Figure S17), and accelerated durability test (Figure S18 and Table S9) (PDF)
