# Supplementary material for: Catalytic Activity and Electrochemical Stability of Ru1–xMxO2 (M = Zr, Nb, Ta): Computational and Experimental Study of the Oxygen Evolution Reaction
Source: ACS Appl Mater Interfaces. 2024 Mar 19;16(13):16373–98. doi: 10.1021/acsami.4c01408 (PMC10995909; doi:10.1021/acsami.4c01408)
Supplement: Supplementary file 1 — am4c01408_si_001.pdf [file am4c01408_si_001.pdf]

Supporting Information

Catalytic Activity and Electrochemical Stability of $Ru_{1-x}M_xO_2$ (M = Zr, Nb, Ta): Computational and Experimental Study of the Oxygen Evolution Reaction

Francisco Ospina-Acevedo,[1, #] Luis A. Albiter,[2, #] Kathleen O. Bailey,[3] Jose Fernando Godínez-Salomón,[3] Christopher P. Rhodes,[2,3*] and Perla B. Balbuena[1*]

[1] Department of Chemical Engineering, Texas A&M University, College Station, TX 77843, United States

[2] Materials Science, Engineering and Commercialization Program, Texas State University, San Marcos, TX 78666, United States

[3] Department of Chemistry and Biochemistry, Texas State University, San Marcos, TX 78666, United States

*Corresponding authors: e-mail: balbuena@tamu.edu; cprhodes@txstate.edu

# *These authors contributed equally to this work.*

Table S1. Physicochemical characteristics of synthesized $Ru_{0.87}Nb_{0.13}O_2$, $Ru_{0.87}Ta_{0.13}O_2$, and $Ru_{0.87}Zr_{0.13}O_2$ obtained from energy-dispersive X-ray spectroscopy (EDS) mapping analysis and X-ray photoelectron spectroscopy (XPS).

Sample	Relative atomic % from synthesis	Relative atomic % from EDS analysis	Surface Composition, XPS (at. %)
$Ru_{0.87}Nb_{0.13}O_2$	$Ru_{0.875}Nb_{0.125}$	$Ru_{0.82}Nb_{0.18}$	$Ru_{0.56}Nb_{0.44}$
$Ru_{0.87}Ta_{0.13}O_2$	$Ru_{0.875}Ta_{0.125}$	$Ru_{0.76}Ta_{0.24}$	$Ru_{0.60}Ta_{0.40}$
$Ru_{0.87}Zr_{0.13}O_2$	$Ru_{0.875}Zr_{0.125}$	$Ru_{0.88}Zr_{0.12}$	$Ru_{0.63}Zr_{0.37}$

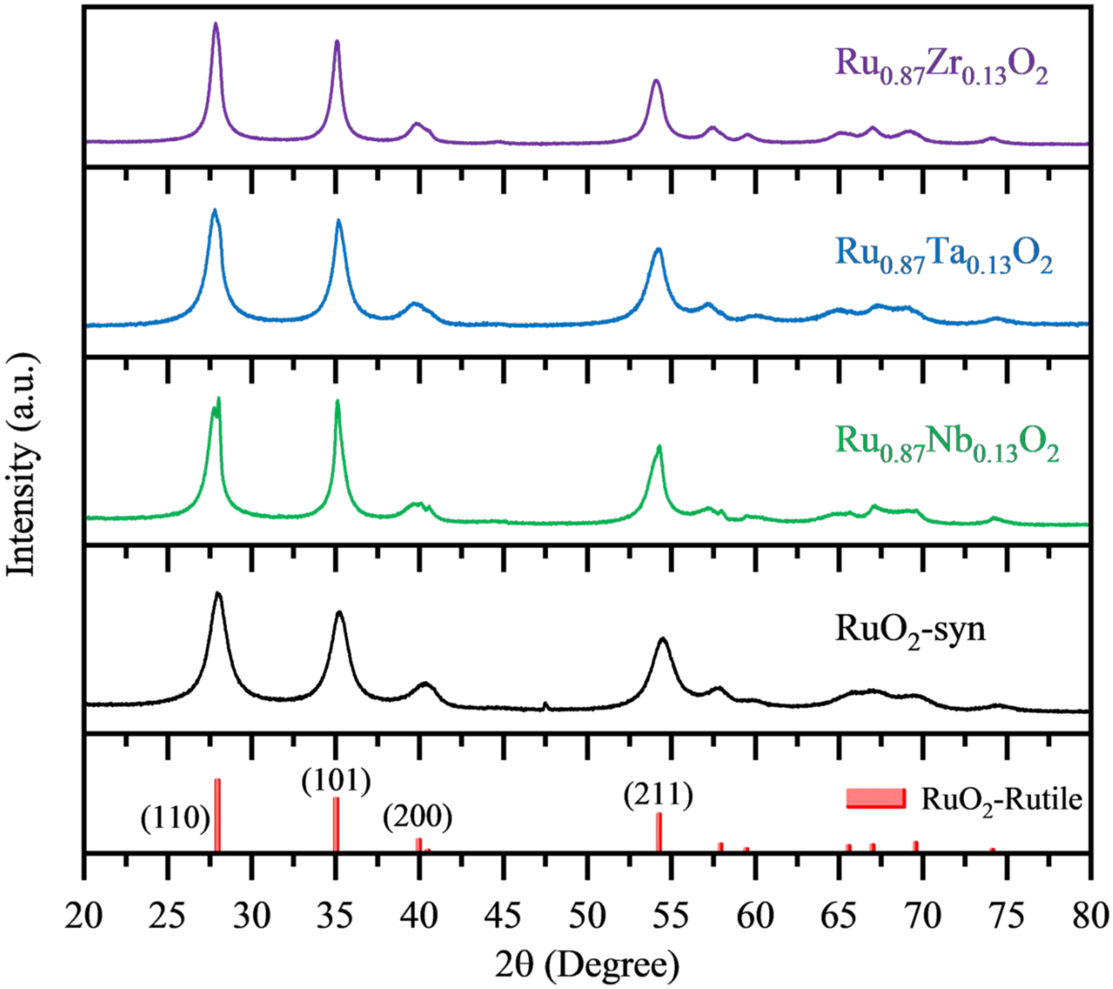

Figure S1. Full scan range of powder X-ray diffraction (XRD) patterns of RuO_2-syn, $Ru_{0.87}Nb_{0.13}O_2$, $Ru_{0.87}Ta_{0.13}O_2$, and $Ru_{0.87}Zr_{0.13}O_2$ and patterns for rutile RuO_2 reference (PDF: 01-071-4825).

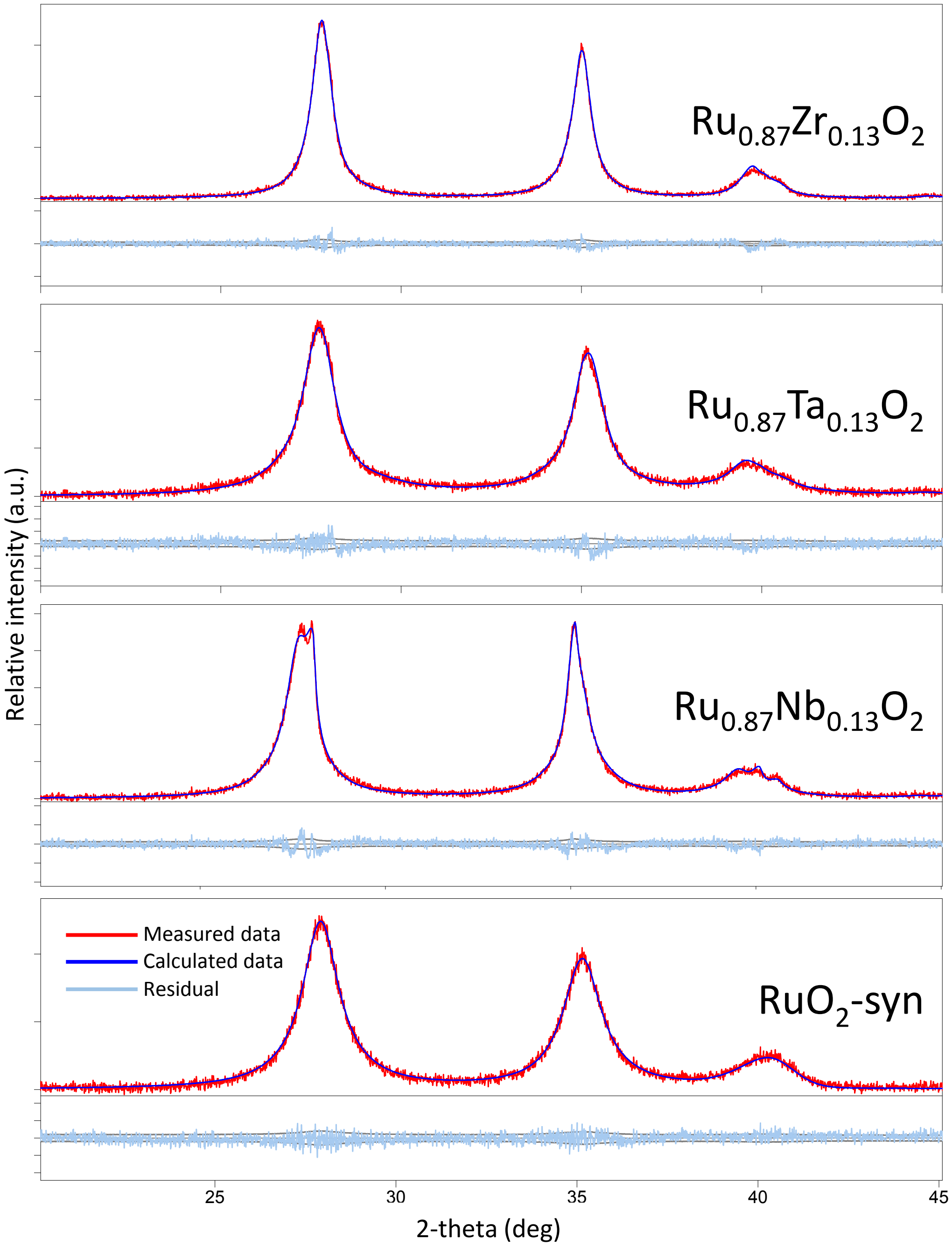

Figure S2. Powder X-ray diffraction (XRD) data in the 20-45 2θ°range and calculated patterns from Rietveld fitting of RuO_2-syn, $Ru_{0.87}Nb_{0.13}O_2$, $Ru_{0.87}Ta_{0.13}O_2$, and $Ru_{0.87}Zr_{0.13}O_2$; additional details are provided in the text.

Table S2. Theoretical and experimental 2θ° values of <110>, <101> and <200> planes; experimental data includes 2θ° values of two phases present within $Ru_{0.87}Nb_{0.13}O_2$, $Ru_{0.87}Ta_{0.13}O_2$, and $Ru_{0.87}Zr_{0.13}O_2$.

Material	2θ° value for lattice planes					
	<110>		<101>		<200>	
	Theoretical	Experimental	Theoretical	Experimental	Theoretical	Experimental
RuO_2-syn	28.21	28.01	35.15	35.22	40.33	40.04
$Ru_{0.87}Nb_{0.13}O_2$	27.8	27.68/28.07	35.27	35.14/35.25	39.8	39.55/40.12
$Ru_{0.87}Ta_{0.13}O_2$	27.81	27.69/28.02	35.27	35.06/35.23	39.88	39.57/40.04
$Ru_{0.87}Zr_{0.13}O_2$	27.8	27.84/28.04	35.27	35.03/35.06	39.8	39.79/40.14

Table S3. Summary of crystallographic parameters, phase percentage, and crystalline domain size RuO_2-syn, $Ru_{0.87}Nb_{0.13}O_2$, $Ru_{0.87}Ta_{0.13}O_2$, and $Ru_{0.87}Zr_{0.13}O_2$ obtained from Rietveld fitting of the experimental XRD patterns. Lattice parameters of a reference RuO_2 (International Centre for Diffraction Data (ICCD) Powder Diffraction File (PDF): 01-071-4825) are included for comparison.

Material	Lattice Parameter (Å)		Phase %	Crystalline domain size (nm)
	a	c		
RuO_2 (PDF: 01-071-4825)	4.51	3.11	-	-
RuO_2-syn	4.493	3.106	100	5.2
$Ru_{0.87}Nb_{0.13}O_2$	4.554	3.067	53	10.5
	4.491	3.101	47	9.5
$Ru_{0.87}Ta_{0.13}O_2$	4.551	3.070	77	5.4
	4.499	3.108	23	5.5
$Ru_{0.87}Zr_{0.13}O_2$	4.527	3.098	90	7.1
	4.489	3.115	10	7.1

Table S4. Details of atomic positions and occupancies obtained from Rietveld refinement of X-ray diffraction patterns of RuO_2, $Ru_{0.87}Nb_{0.13}O_2$, $Ru_{0.87}Ta_{0.13}O_2$, and $Ru_{0.87}Zr_{0.13}O_2$. Rietveld fitting utilized a tetragonal rutile phase with the space group $P4_2/mnm$, point group (D_{4h}^{14}), and two molecular formula units per unit cell (Z=2).

Material	Phase	Atom	Wyckoff site	x	y	z	Occ.
RuO_2-syn	Phase 1	Ru	2a	0	0	0	1
		O	4f	0.1946	0.8053	0.5	1
$Ru_{0.87}Nb_{0.13}O_2$	Phase 1	Ru	2a	0	0	0	0.80
		Nb	2a	0	0	0	0.20
		O	4f	0.2014	0.7986	0.5	1
	Phase 2	Ru	2a	0	0	0	1
		Nb	2a	0	0	0	0
		O	4f	0.2016	0.7983	0.5	1
$Ru_{0.87}Ta_{0.13}O_2$	Phase 1	Ru	2a	0	0	0	0.77
		Ta	2a	0	0	0	0.23
		O	4f	0.1984	0.8016	0.5	1
	Phase 2	Ru	2a	0	0	0	1
		Ta	2a	0	0	0	0
		O	4f	0.1888	0.8112	0.5	1
$Ru_{0.87}Zr_{0.13}O_2$	Phase 1	Ru	2a	0	0	0	0.87
		Zr	2a	0	0	0	0.13
		O	4f	0.1990	0.8009	0	1
	Phase 2	Ru	2a	0	0	0	1
		Zr	2a	0	0	0	0
		O	4f	0.1860	0.8140	0.5	1

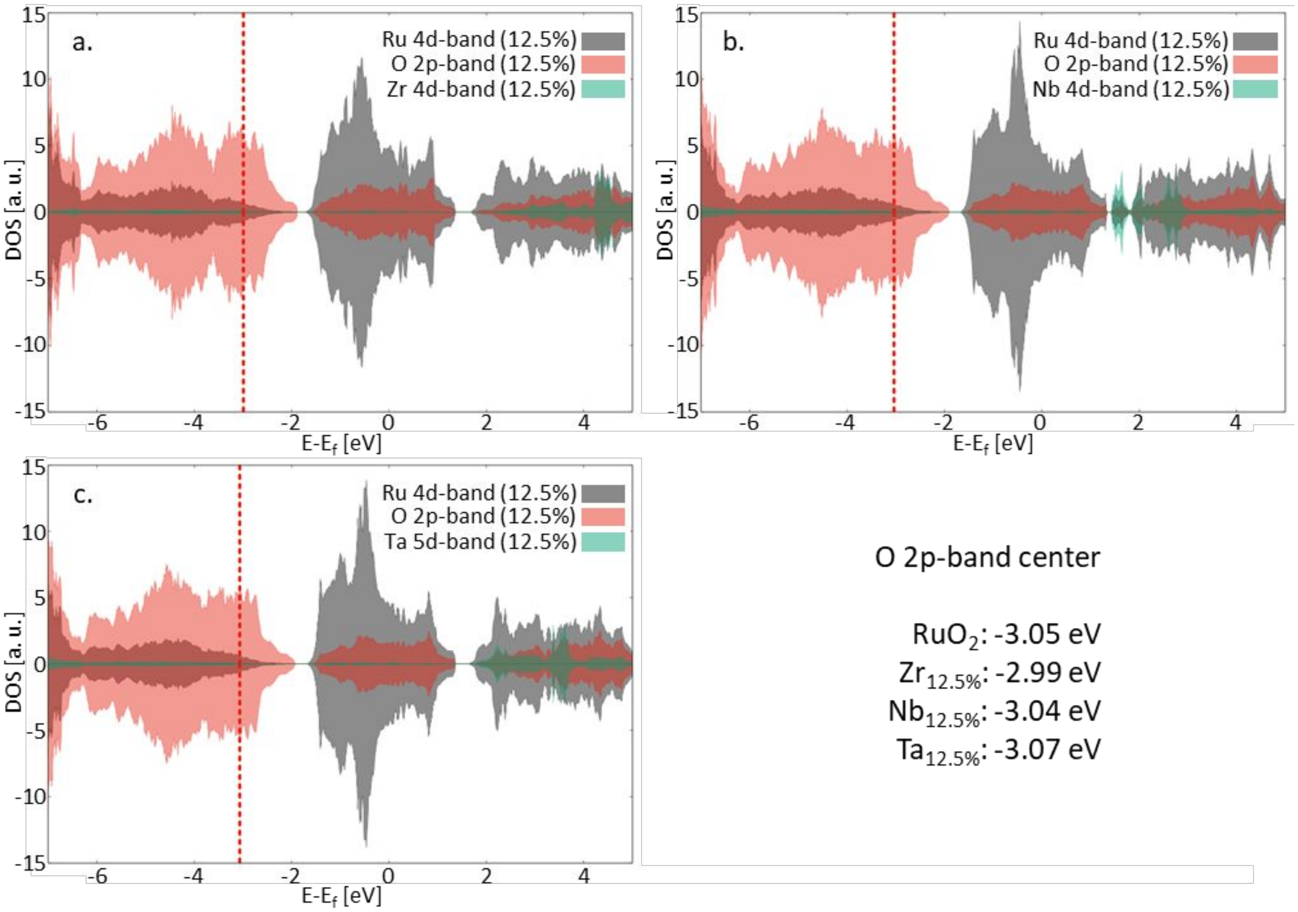

Figure S3. Contributions of the Ru-4d, O-2p-4d and M-d (Zr-4d, Nb-4d, and Ta-5d) bands to the total density of states (TDOS), respectively for each system at 12.5% M. Ru-4d band is represented in black, O-2p band in red, and M-d band in green for (a) $Ru_{0.875}Nb_{0.125}O_2$, (b) $Ru_{0.875}Zr_{0.125}O_2$, and (c) $Ru_{0.875}Ta_{0.125}O_2$. The table in the bottom, left side presents the O-2p-band center calculated for the $Ru_{0.875}M_{0.125}O_2$ bulk systems, shown as vertical red, dashed lines in each figure.

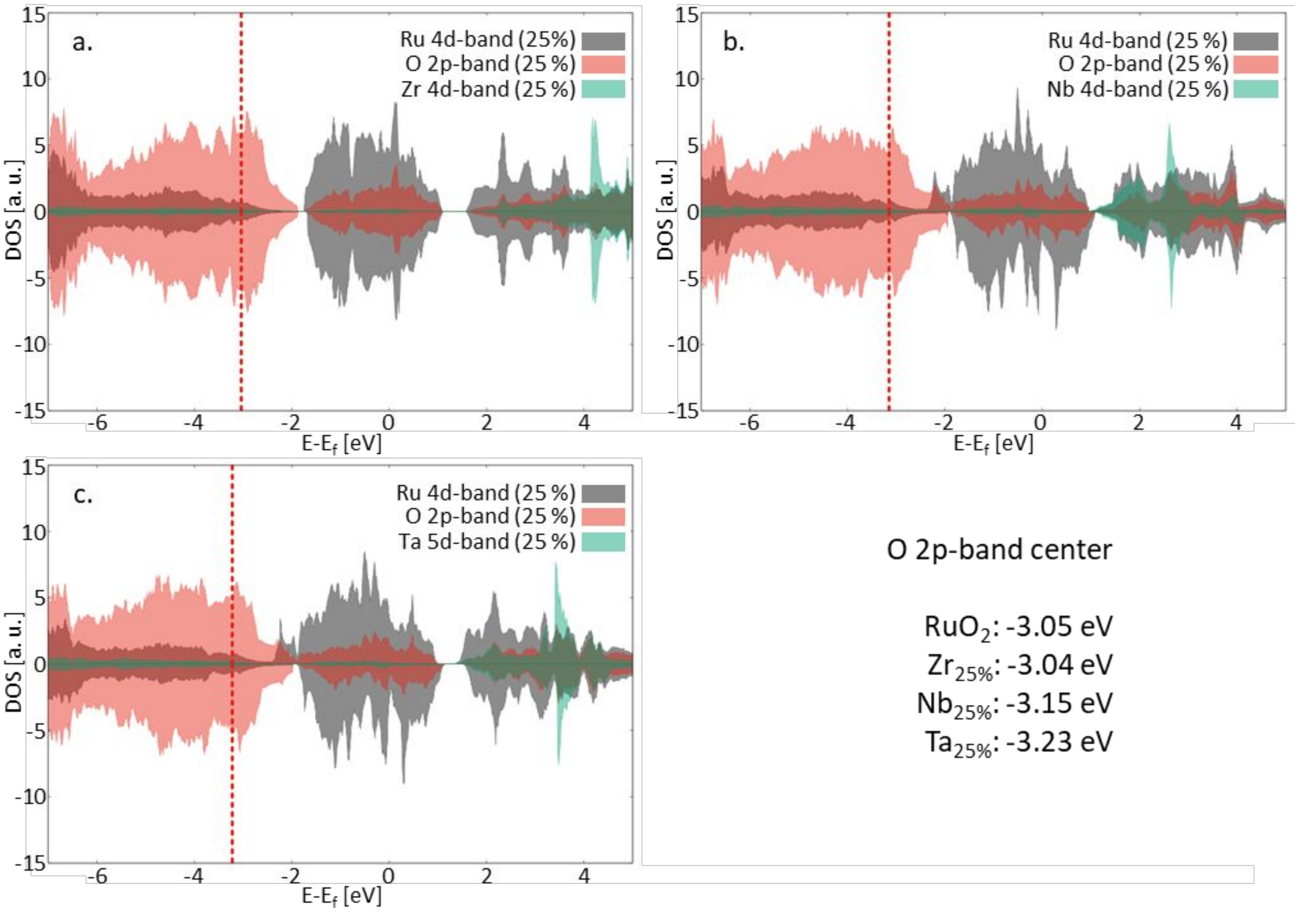

Figure S4. Contributions of the Ru-4d, O-2p-4d and M-d (Zr-4d, Nb-4d, and Ta-5d) bands to the total density of states (TDOS), respectively for each system at 25% M. Ru-4d band is represented in black, O-2p band in red, and M-d band in green for (a) $Ru_{0.75}Nb_{0.25}O_2$, (b) $Ru_{0.75}Zr_{0.25}O_2$, and (c) $Ru_{0.75}Ta_{0.25}O_2$. The table in the bottom, left side presents the O-2p-band center calculated for the $Ru_{0.75}M_{0.25}O_2$ bulk systems, shown as vertical red, dashed lines in each figure.

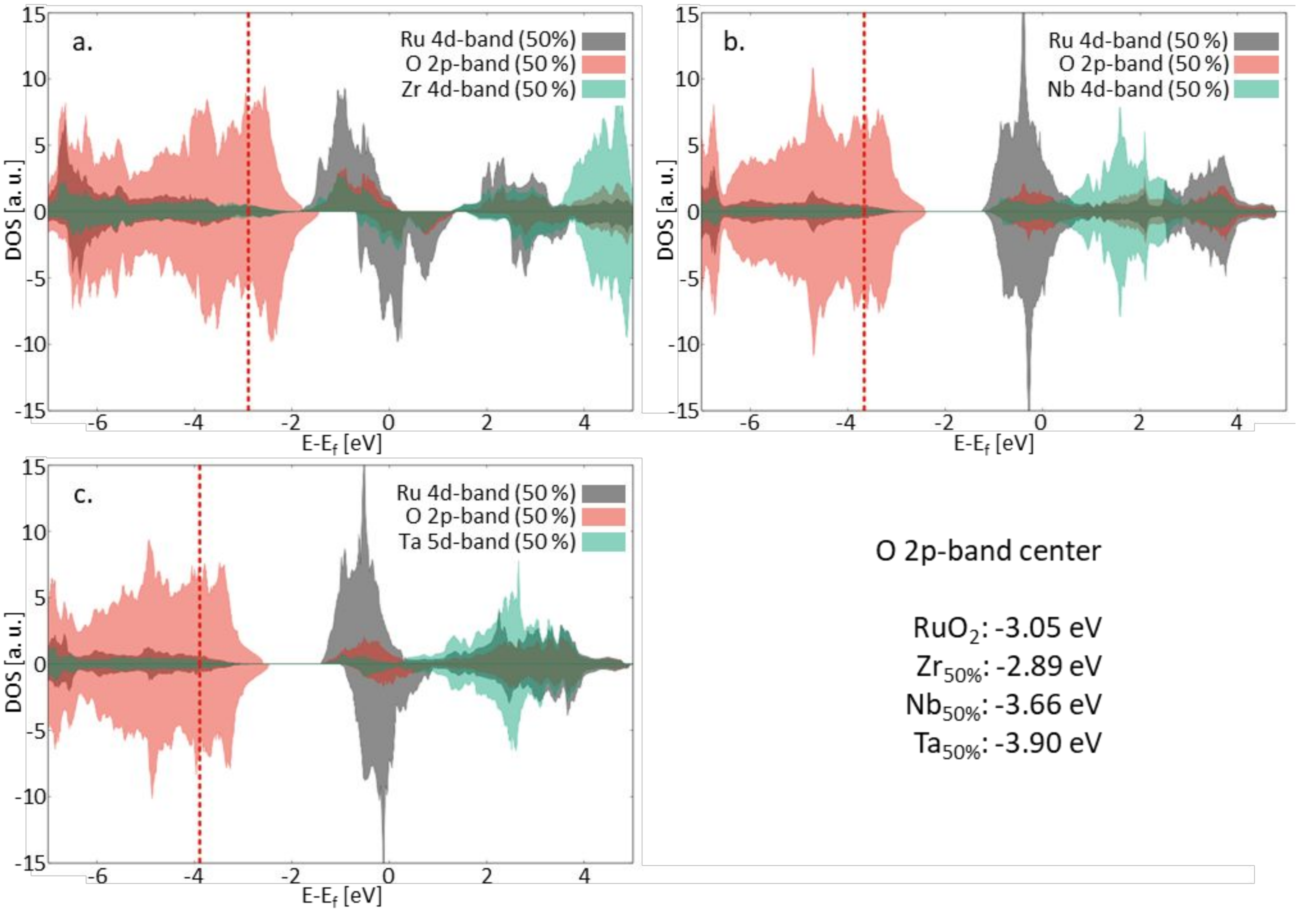

Figure S5. Contributions of the Ru-4d, O-2p-4d and M-d (Zr-4d, Nb-4d, and Ta-5d) bands to the total density of states (TDOS), respectively for each system at 50% M. Ru-4d band is represented in black, O-2p band in red, and M-d band in green for (a) $Ru_{0.50}Nb_{0.50}O_2$, (b) $Ru_{0.50}Zr_{0.50}O_2$, and (c) $Ru_{0.50}Ta_{0.50}O_2$. The table in the bottom, left side presents the O-2p-band center calculated for the $Ru_{0.50}M_{0.50}O_2$ bulk systems, shown as vertical red, dashed lines in each figure.

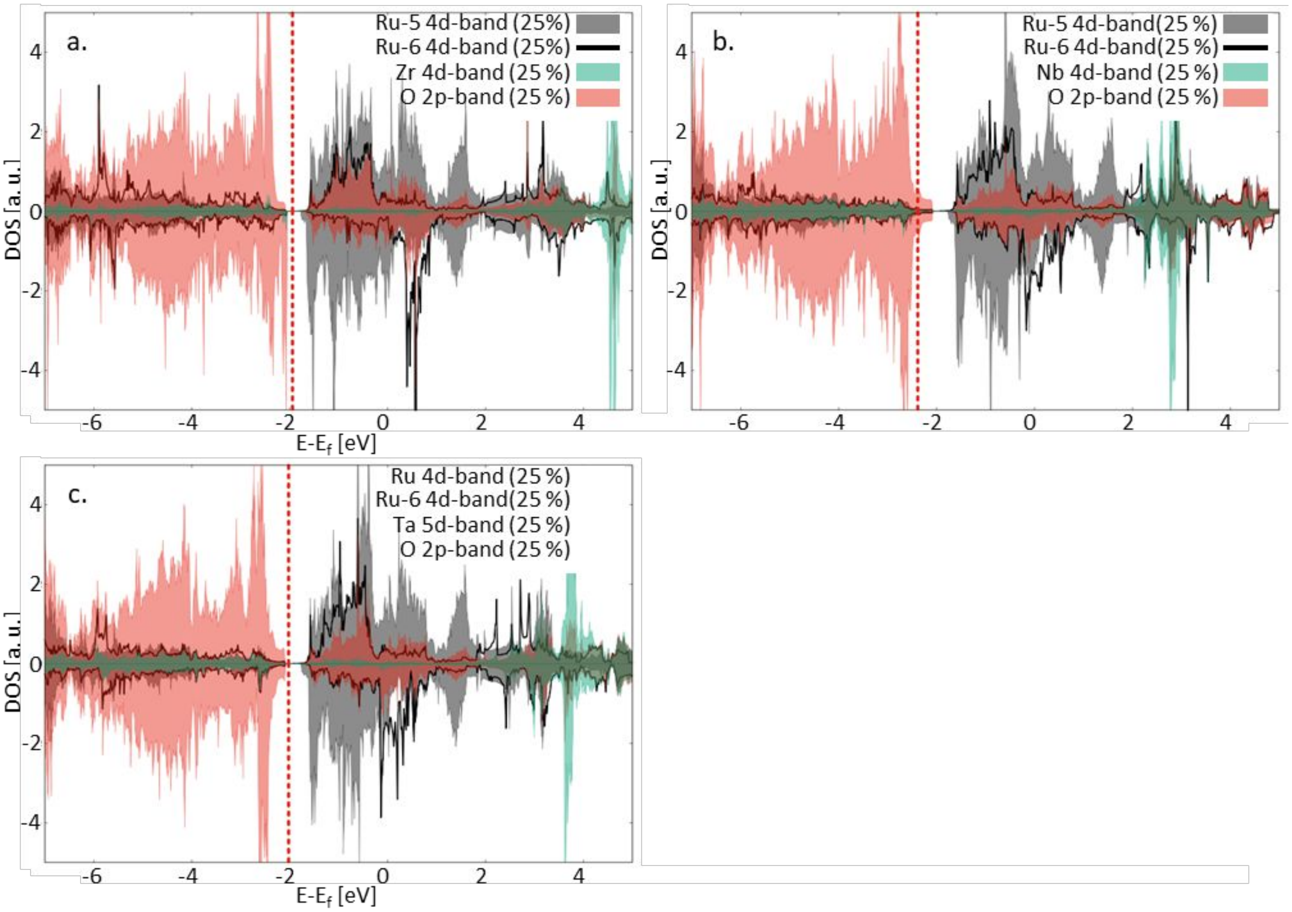

Figure S6. Contributions of the Ru-5C-4d, Ru-6C-4d, O-2p-4d and M-6D-d (Zr-4d, Nb-4d, and Ta-5d) bands to the (110) top layer density of states (TDOS), respectively for each system at 25% M-6D. Ru-5C-4d band is represented in grey, Ru-6C-4d ban in solid black line, O-2p band in red, and M-d band in green for (a) $Ru_{0.75}Nb_{0.25}O_2$, (b) $Ru_{0.75}Zr_{0.25}O_2$, and (c) $Ru_{0.75}Ta_{0.25}O_2$. The vertical red dashed line in each figure represents the O-2p band center.

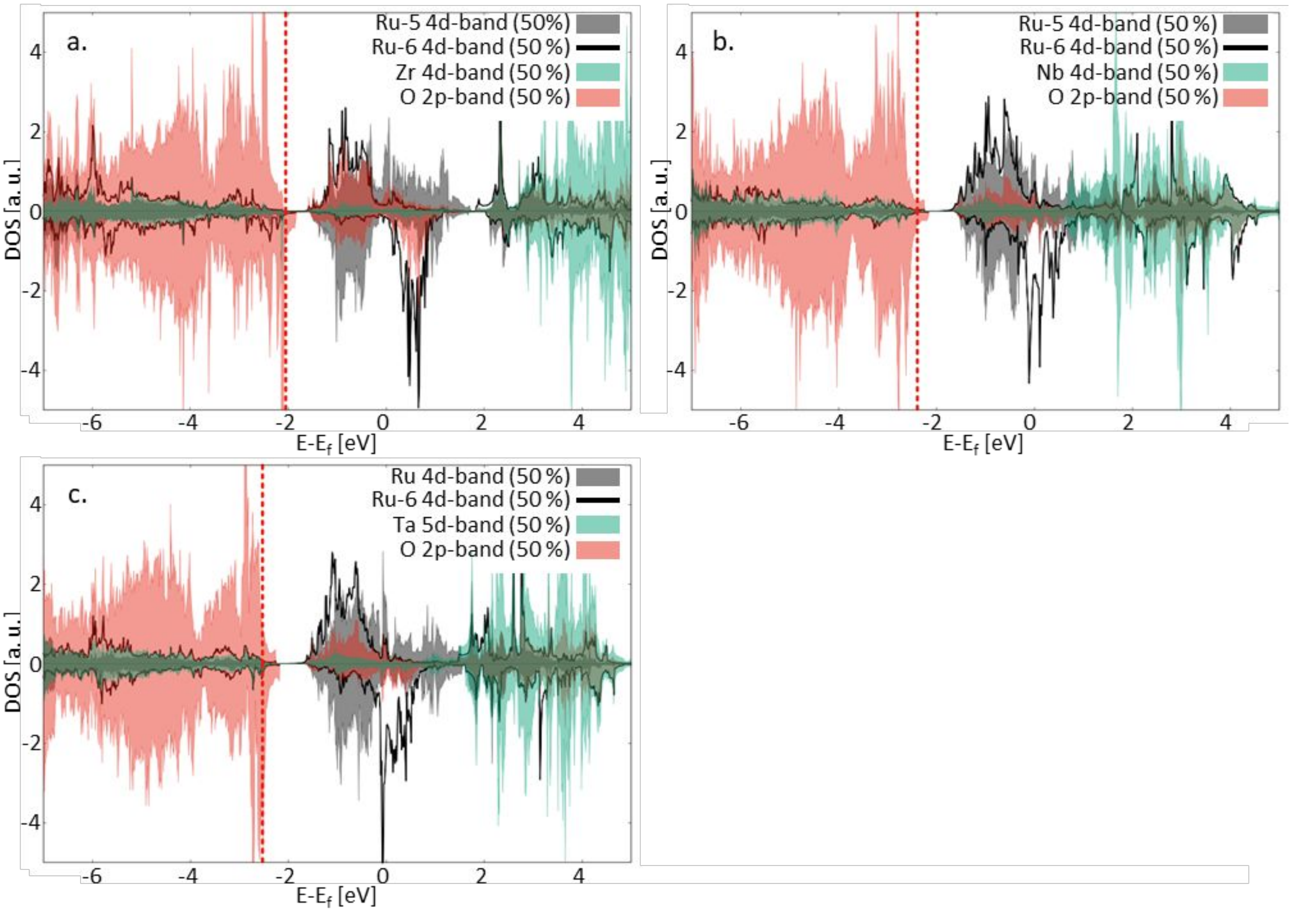

Figure S7. Contributions of the Ru-5C-4d, Ru-6C-4d, O-2p-4d and M-d (Zr-4d, Nb-4d, and Ta-5d) bands to the (110) top layer density of states (TDOS), respectively for each system at 50% M. Ru-5C-4d band is represented in grey, Ru-6C-4d ban in solid black line, O-2p band in red, and M-d band in green for (a) $Ru_{0.50}Nb_{0.50}O_2$-(110), (b) $Ru_{0.50}Zr_{0.50}O_2$-(110), and (c) $Ru_{0.50}Ta_{0.50}O_2$-(110). The vertical red dashed line in each figure represents the O-2p band center.

Table S5. Left: Calculated values for the Ru 4d-band and O 2p-band centers from the $Ru_{1-X}M_XO_2$ bulk systems (d-band center ref: -1.10 eV, p-band center ref: -3.05 eV)., right: calculated values for the Ru-5C 4d-band and O_B 2p-band centers from the $Ru_{1-X}M_XO_2$-(110) top layer (d-band center ref: -1.26 eV, p-band center ref: -2.49 eV).

Descriptor	$Ru_{1-X}M_XO_2$ Bulk				$Ru_{1-X}M_XO_2$-(110) Surface			
	[M]	Zr	Nb	Ta	[M]	Zr	Nb	Ta
d-band center [eV]	12.5%	-1.25	-1.19	-1.20	25%-5D	-1.59	-1.35	-1.34
	25%	-1.38	-1.40	-1.38	25%-6D	-1.48	-1.36	-1.38
	50%	-1.32	-0.96	-0.93	50%	-1.62	-1.31	-1.24
p-band center [eV]	12.5%	-2.99	-3.04	-3.07	25%-5D	-2.51	-2.60	-2.57
	25%	-3.04	-3.15	-3.23	25%-6D	-1.94	-2.38	-2.02
	50%	-2.89	-3.66	-3.90	50%	-2.05	-2.40	-2.53

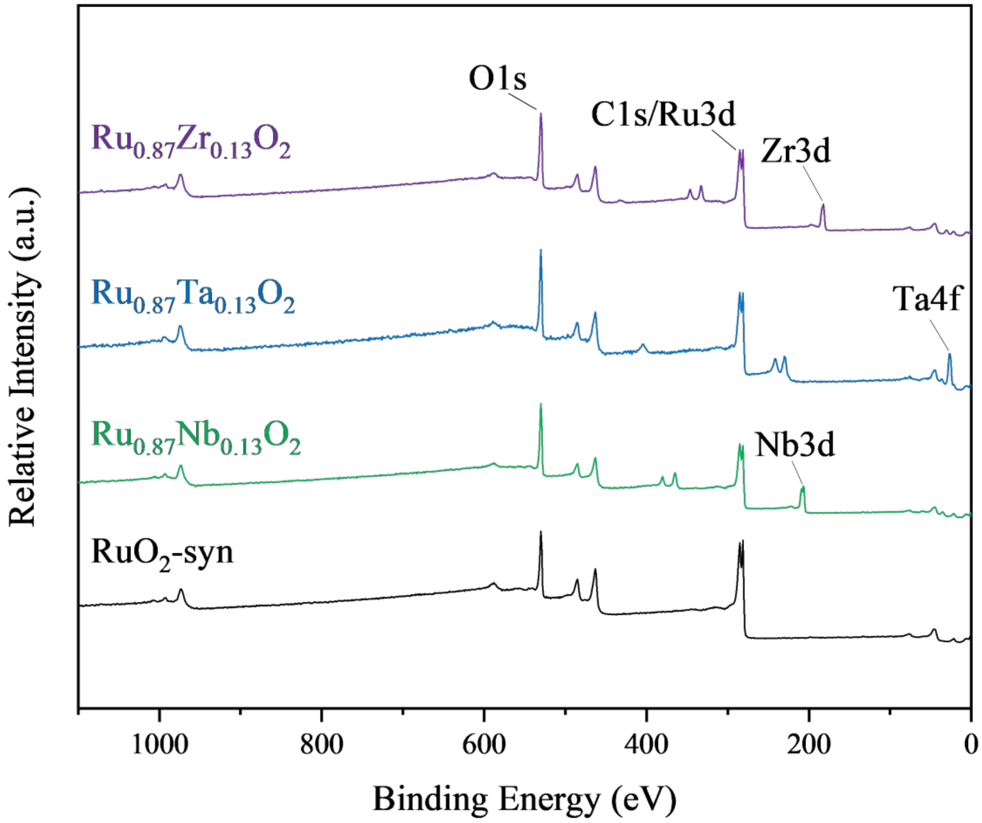

Figure S8. X-ray photoelectron spectroscopy (XPS) survey spectra of RuO_2-syn, $Ru_{0.87}Nb_{0.13}O_2$, $Ru_{0.87}Ta_{0.13}O_2$, and $Ru_{0.87}Zr_{0.13}O_2$, showing the O1s, C1s/Ru3d peaks and additional Zr3d, Ta4f, and Nb3d peaks of the metal substituted samples. The XPS survey spectra were analyzed using AVANTAGE v5.91 software (Thermo Fisher Scientific) and utilized peak areas and sensitivity factors to determine the relative atomic composition of Ru and other metals (Nb, Ta, Zr) at the surface; the relative atomic composition from XPS analysis is presented in Table S1.

Table S6. Peaks, binding energies, and relative areas from peak fitting analysis of X-ray photoelectron spectra of RuO_2-syn, $Ru_{0.87}Nb_{0.13}O_2$, $Ru_{0.87}Ta_{0.13}O_2$, and $Ru_{0.87}Zr_{0.13}O_2$ and assignments from prior studies of the O1s region,[1] C 1s-Ru 3d region,[1-3] Ru 3p region,[1-3] Nb 3d region,[4-6] Ta 4f region[7, 8] and Zr 3d regions.[9] The relative error in binding energies is estimated as ± 0.1 eV.

Region	Peak Label	Assignment	RuO_2-syn		$Ru_{0.87}Nb_{0.13}O_2$		$Ru_{0.87}Ta_{0.13}O_2$		$Ru_{0.87}Zr_{0.13}O_2$	
			Binding Energy (eV)	Area Ratio	Binding Energy (eV)	Area Ratio	Binding Energy (eV)	Area Ratio	Binding Energy (eV)	Area Ratio
O 1s	O1s, A	Ru-O	529.4	1.00	529.5	1.00	529.5	1.00	529.4	1.00
	O1s, B	Ru-O sat, M-O	530.7	0.27	530.7	0.26	530.8	0.41	530.8	0.45
	O1s, C	C-O	532.3	0.18	532.2	0.12	532.2	0.17	532.2	0.19
C 1s-Ru 3d	Ru $3d_{5/2}$	Ru^{4+}	280.8	1.00	280.8	1.00	280.8	1.00	280.8	1.00
	Ru $3d_{5/2}$ sat	Ru^{4+}	282.9	0.52	282.8	0.54	282.8	0.55	282.8	0.58
	Ru $3d_{3/2}$	Ru^{4+}	285.1	0.65	285.1	0.67	285.1	0.64	285.1	0.74
	Ru$3d_{3/2}$ sat	Ru^{4+}	286.7	0.38	286.6	0.45	286.6	0.33	286.8	0.42
	C 1s	C-C	284.8	0.03	284.8	0.03	284.8	0.02	284.8	0.04
Ru 3p	Ru $3p_{3/2}$	Ru^{4+}	262.5	1.00	462.5	1.00	462.5	1.00	462.5	1.00
	Ru $3p_{3/2}$ sat	Ru^{4+}	465.8	0.39	465.6	0.29	465.8	0.44	465.6	0.41
Nb 3d	Nb $3d_{5/2}$	Nb^{5+}	-	-	206.6	1.00	-	-	-	-
	Nb $3d_{3/2}$	Nb^{5+}	-	-	209.3	0.89	-	-	-	-
Ta 4f	Ta $4f_{7/2}$	Ta^{5+}	-	-	-	-	25.6	1.00	-	-
	Ta $4f_{5/2}$	Ta^{5+}	-	-	-	-	27.4	0.78	-	-
Zr 3d	Zr $3d_{5/2}$	Zr^{4+}	-	-	-	-	-	-	181.9	1.00
	Zr $3d_{3/2}$	Zr^{4+}	-	-	-	-	-	-	184.3	0.71

Table S7. Binding energies of the O 2s, O 2p$_\sigma$ and O 2p$_\pi$ peaks obtained from X-ray photoelectron spectroscopy of RuO_2-syn, $Ru_{0.87}Nb_{0.13}O_2$, $Ru_{0.87}Ta_{0.13}O_2$, and $Ru_{0.87}Zr_{0.13}O_2$.

Sample	O 2s	O 2p$_\sigma$	O 2p$_\pi$	Ru 4d
RuO_2-syn	21.2	7.1	5.0	0.6
$Ru_{0.87}Nb_{0.13}O_2$	21.3	7.1	5.0	0.6
$Ru_{0.87}Ta_{0.13}O_2$	21.5	7.1	5.0	0.6
$Ru_{0.87}Zr_{0.13}O_2$	21.1	7.0	4.9	0.5

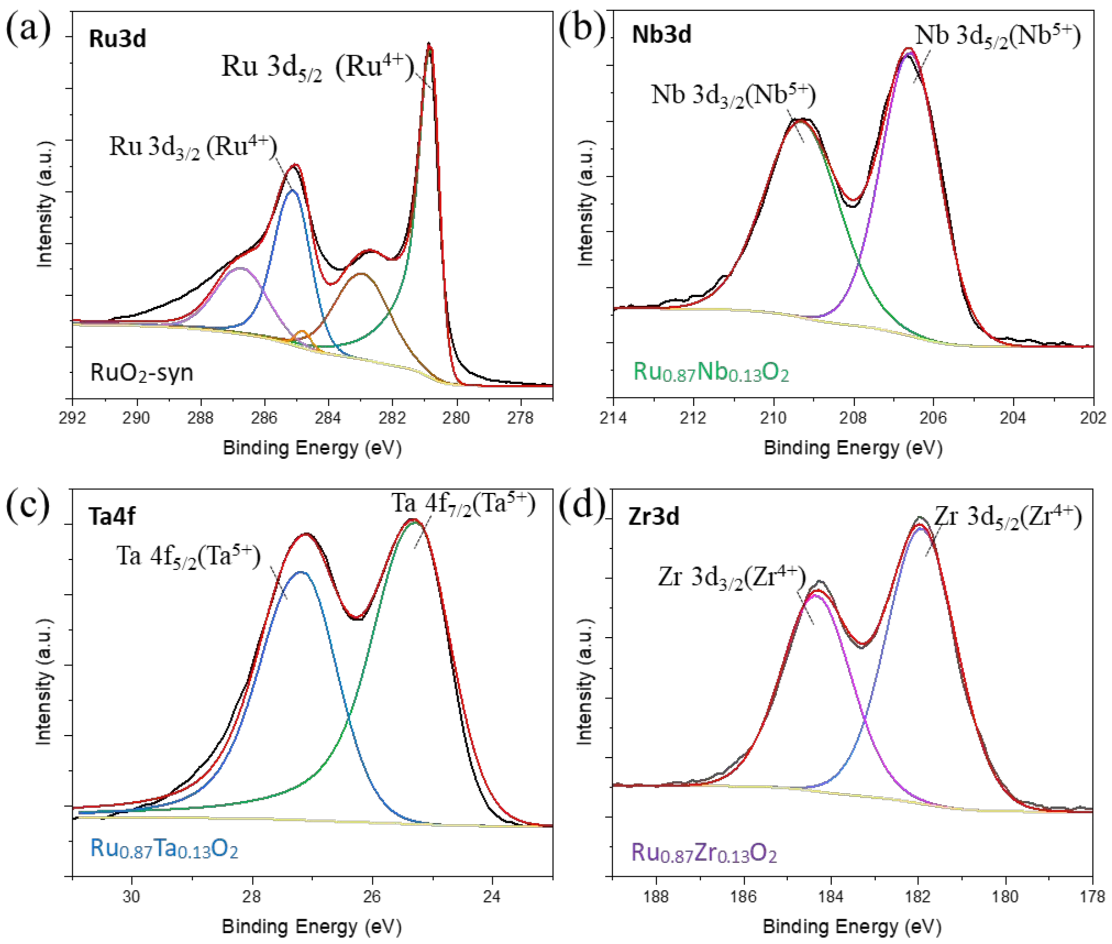

Figure S9. X-ray photoelectron spectra of (a) Ru3d (RuO_2-syn), (b) Nb3d ($Ru_{0.87}Nb_{0.13}O_2$), (c) Ta4f ($Ru_{0.87}Ta_{0.13}O_2$), and (d) Zr3d ($Ru_{0.87}Zr_{0.13}O_2$).

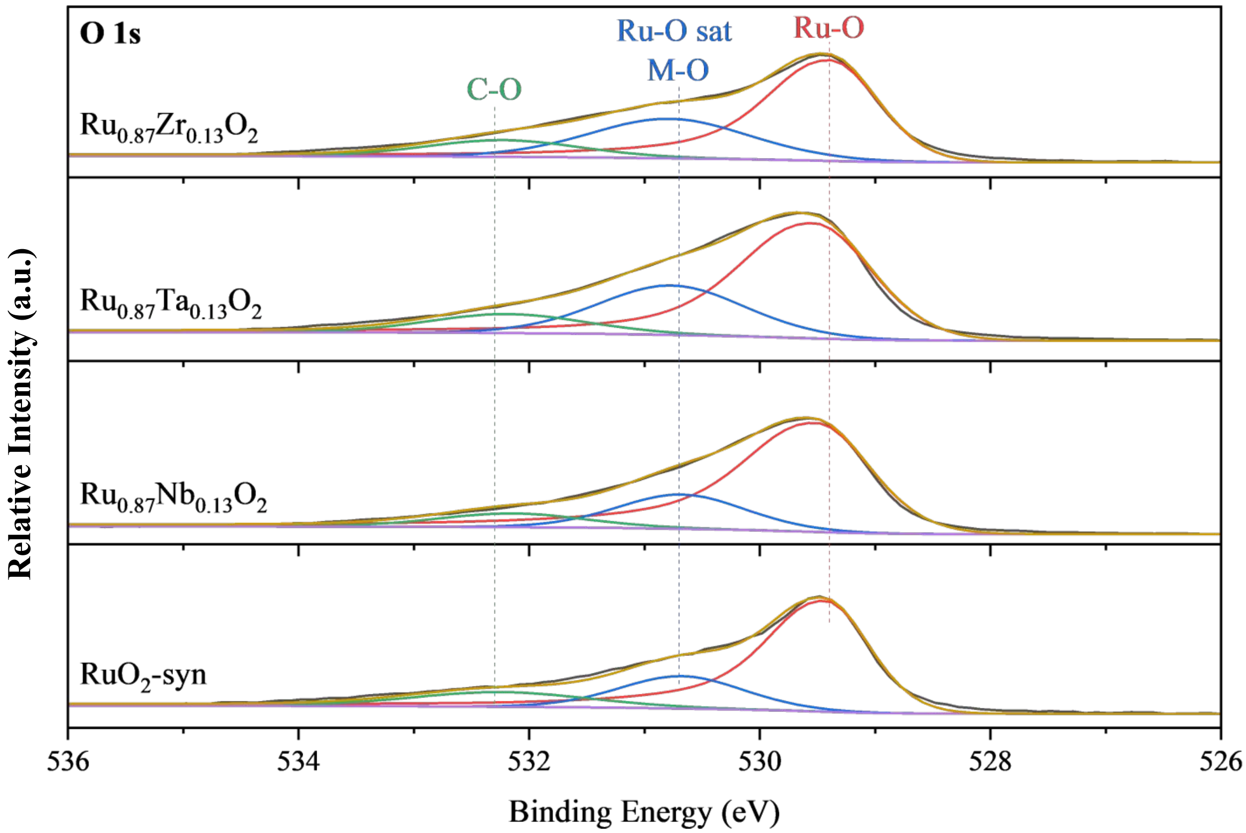

Figure S10. Fitted X-ray photoelectron spectra of the O 1s region of RuO_2-syn, $Ru_{0.87}Nb_{0.13}O_2$, $Ru_{0.87}Ta_{0.13}O_2$, and $Ru_{0.87}Zr_{0.13}O_2$

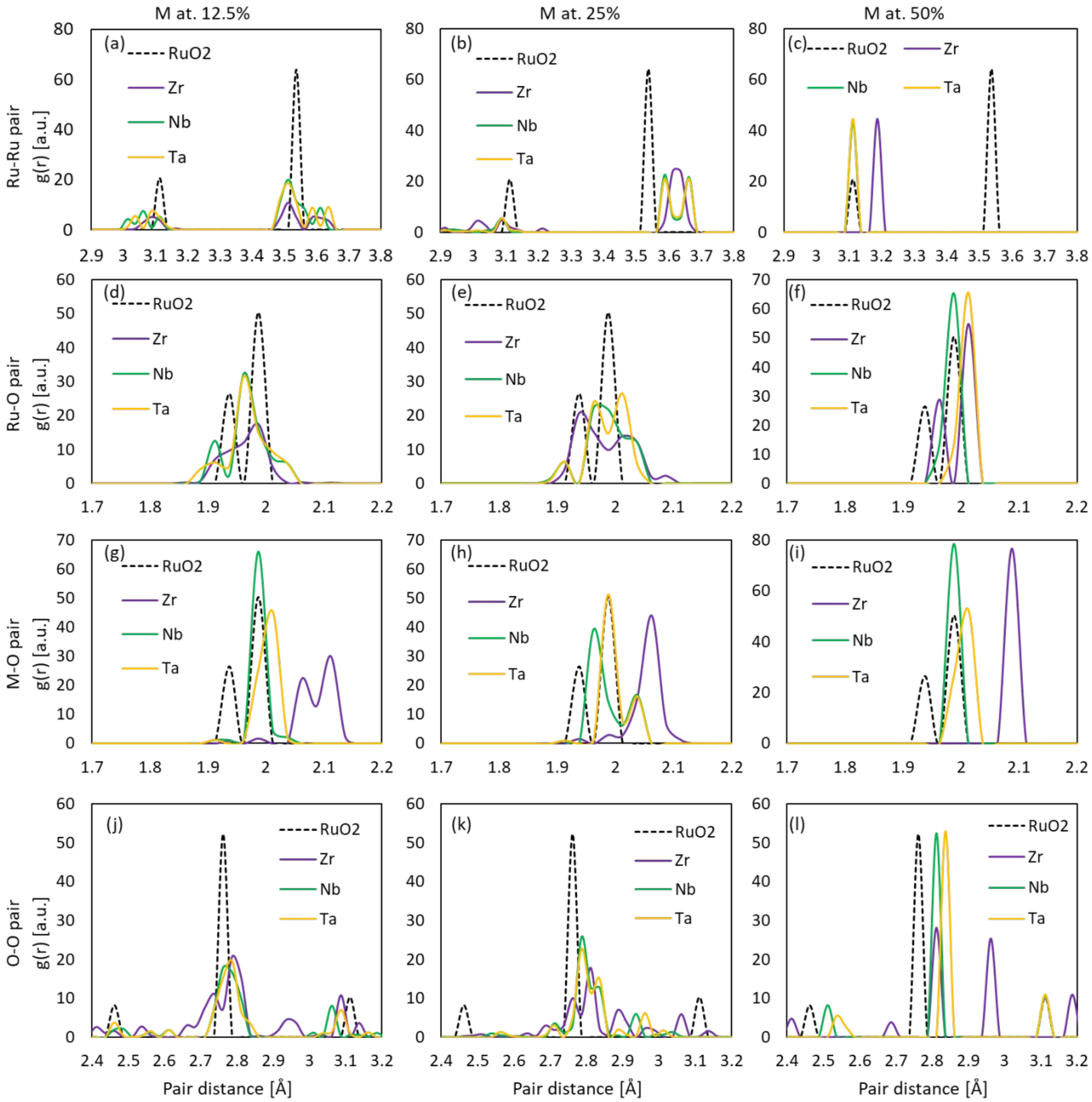

Figure S11. Pair radial distribution function (rdf) for the $Ru_{1-X}M_XO_2$ (M = Zr, Nb, Ta) bulk systems for (a to c) Ru-Ru pairs; (d to f) Ru-O pairs; (g to i) M-O pairs; and (j to l) O-O pairs. Each column presents the data from the systems with M substitution at 12.5%, 5%, and 50%, respectively. Color code: black (dashed line), from RuO_2; purple, $Ru_{1-X}Zr_XO_2$ systems; green, $Ru_{1-X}Nb_XO_2$ systems; yellow; , $Ru_{1-X}Ta_XO_2$ systems.

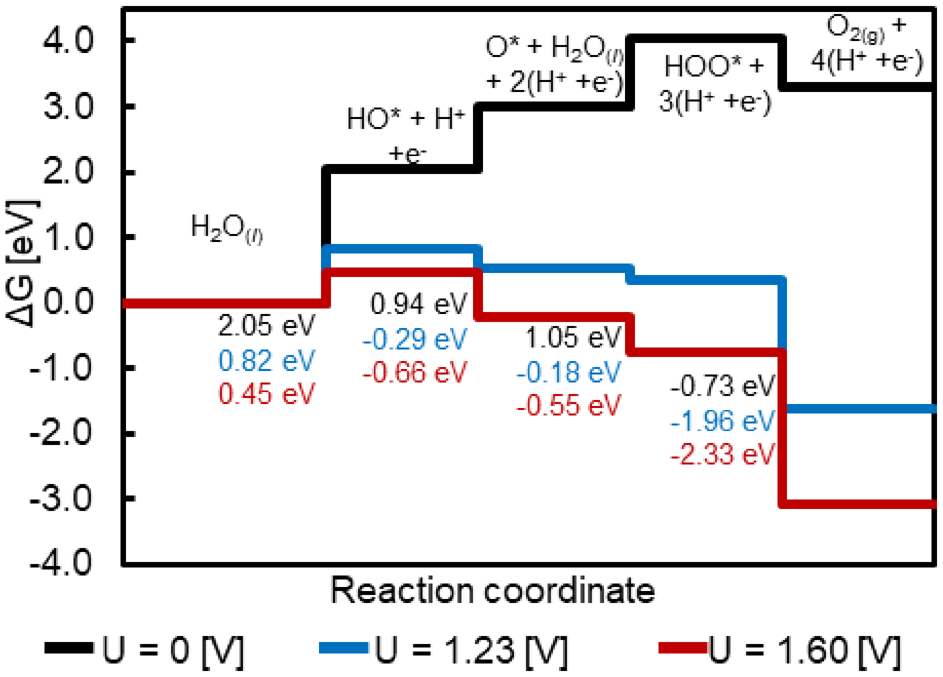

Figure S12. Gibbs free energy at pH ~ 0 diagram for the OER activity from water adsorption to oxygen release evaluated on Ru-5C active sites on the pristine $RuO_2(110)$ surface. DG is evaluated at three different potentials: U = 0 V (black), 1.23 V (blue), and 1.60 V (red).

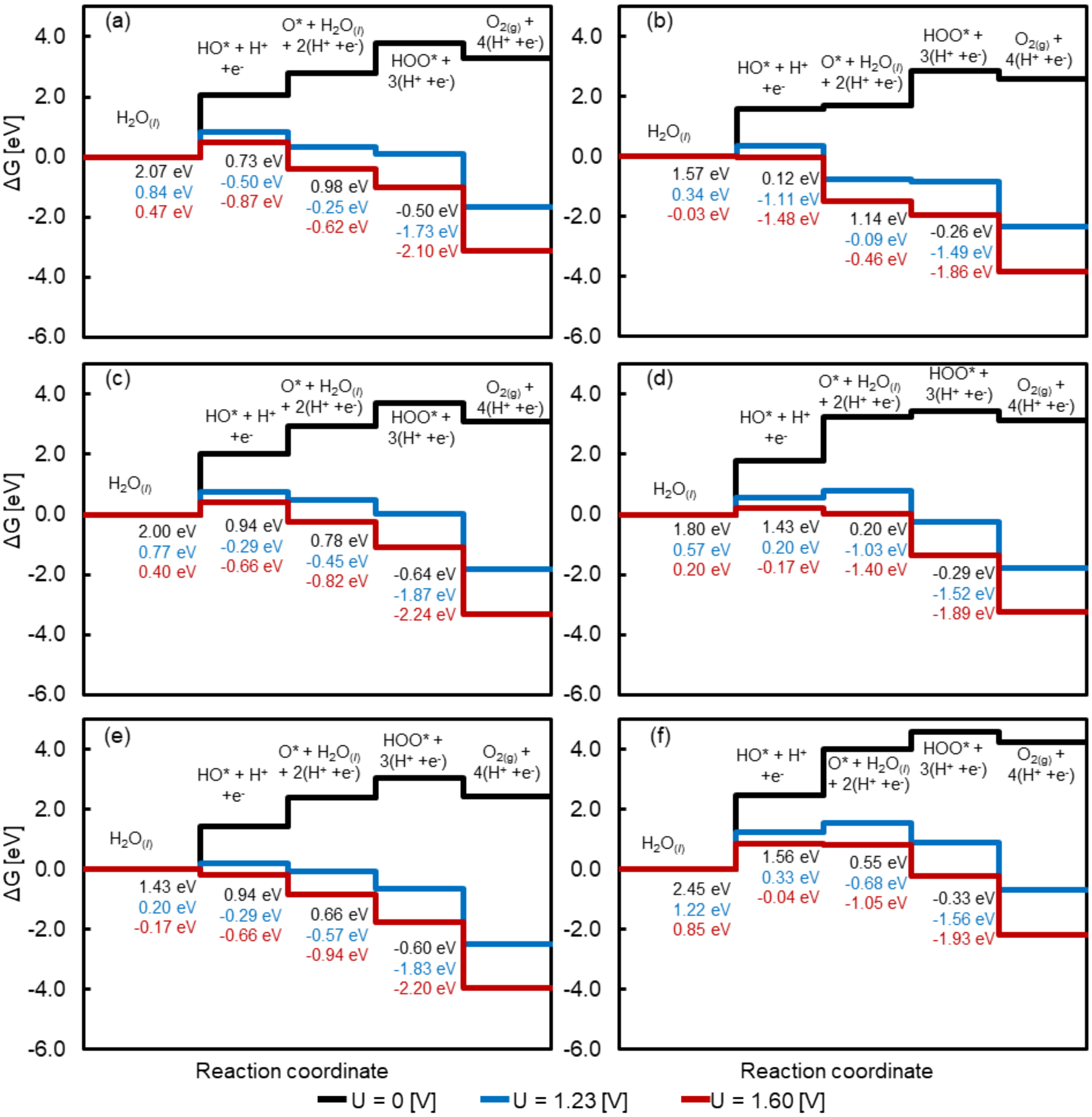

Figure S13. Gibbs free energy at pH ~ 0 diagram for the OER activity from water adsorption to oxygen release evaluated on Ru-5C (right column) and M-5D (left column) active sites on $Ru_{1-x}M_xO_2$-(110) 25%-5D surfaces for (a, b) Zr, (c, d) Nb, and (e, f) Ta substituted metal site surfaces. DG is evaluated at three different potentials: U = 0 V (black), 1.23 V (blue), and 1.60 V (red).

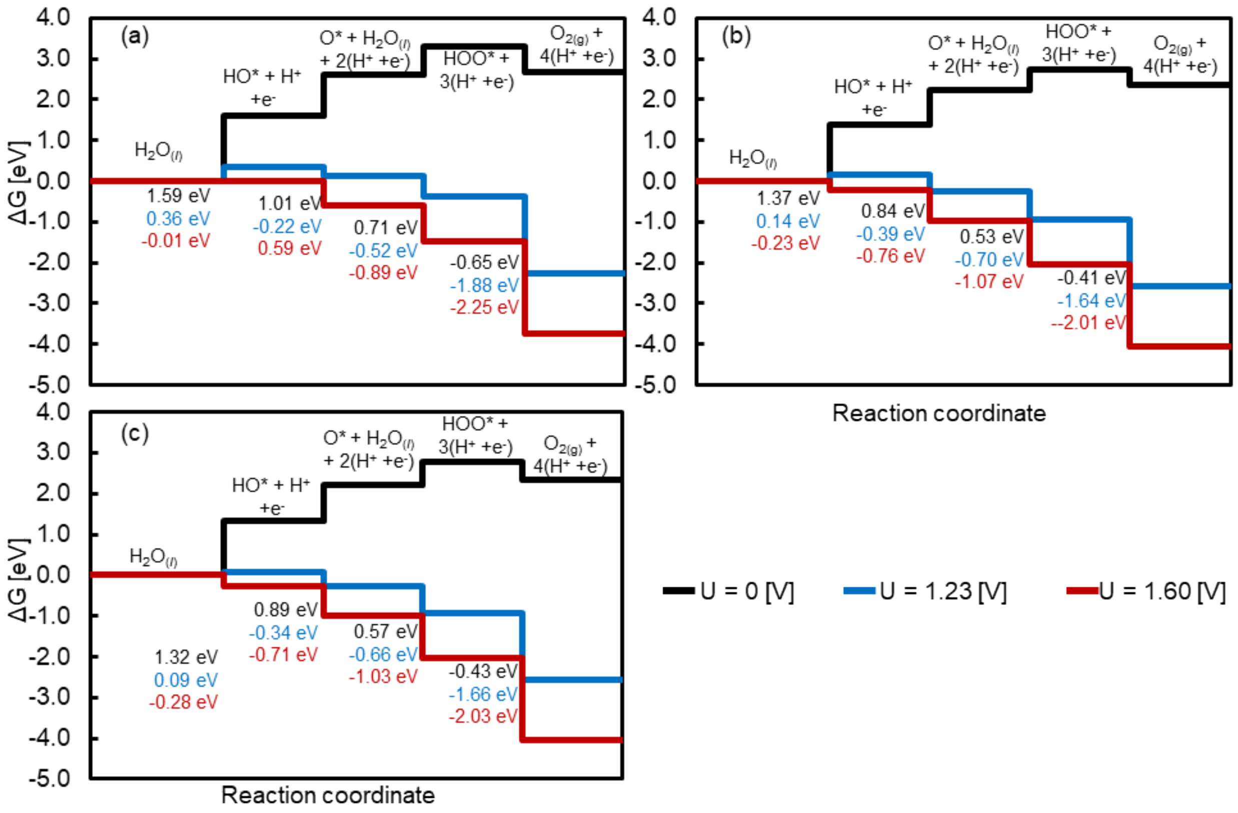

Figure S14. Gibbs free energy at pH ~ 0 diagram for the OER activity from water adsorption to oxygen release evaluated on Ru-5C active sites on $Ru_{1-x}M_xO_2$-(110) 25%-6D surfaces for (a) Zr, (b) Nb, and (c) Ta substituted metal site surfaces. DG is evaluated at three different potentials: U = 0 V (black), 1.23 V (blue), and 1.60 V (red).

Table S8. Activity descriptor $\Delta G_{O*} - \Delta G_{OH*}$ [eV], and negative value of the theoretical overpotential -η^{OER} [V] from the $Ru_{1-x}M_xO_2$-(110) active sites

Slab		Ads. Site	Activity descriptor	-η^{OER}
RuO_2		Ru	1.37	-0.60
25-5D	Ru-sites	Zr	1.55	-0.42
		Nb	1.39	-0.58
		Ta	1.35	-0.62
	M-sites	Zr	2.70	-1.47
		Nb	1.29	-0.68
		Ta	1.68	-0.45
25-6D	Ru-sites	Zr	1.42	-0.55
		Nb	1.36	-0.61
		Ta	1.38	-0.59

For the 25%-5D surfaces, the Ru-5 sites activity descriptor exhibit a variation of 13.14%, 1.46%, and -1.46% (1.55 eV, 1.39 eV, and 1.35 eV) from their values with respect to the 1.37 eV from the pristine surface on the Zr, Nb, and Ta, substituted surfaces, respectively. On the other hand, the activity descriptor for the M-5 sites ranges from 1.17 eV to 2.70 eV (2.70 eV, 1.29 eV, and 1.68 eV) for Zr, Nb, and Ta substituted surfaces, respectively. The 25%-6D surfaces show similar trend of values for the Ru-5 sites activity descriptor, with higher deprotonation energy from the active sites belonging to the Zr doped surface (1.42 eV), followed by the sites on the Ta doped surfaces (1.38 eV), showing similar behavior for the sites on Nb and Ta doped surfaces (1.36 eV for each one).

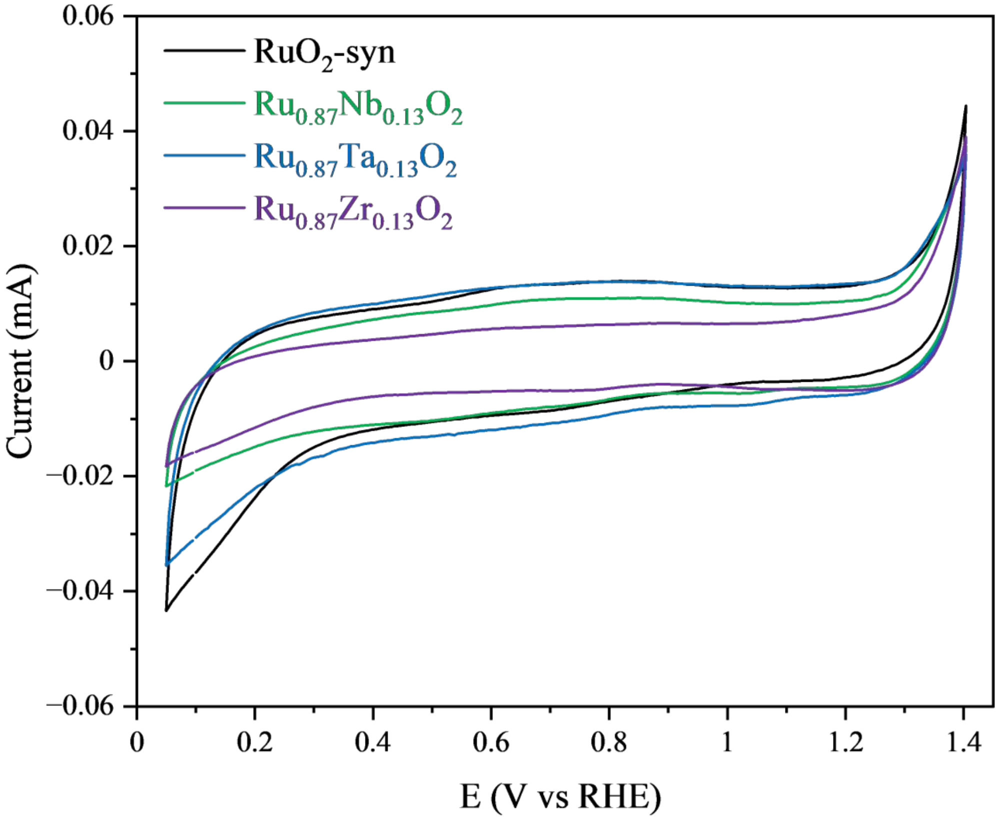

Figure S15. Comparison of the cyclic voltametric scans of RuO_2-syn $Ru_{0.87}Nb_{0.13}O_2$, $Ru_{0.87}Ta_{0.13}O_2$, and $Ru_{0.87}Zr_{0.13}O_2$ in an Au rotating disk electrode in the 0.1 V to 1.4 V_{RHE} region.

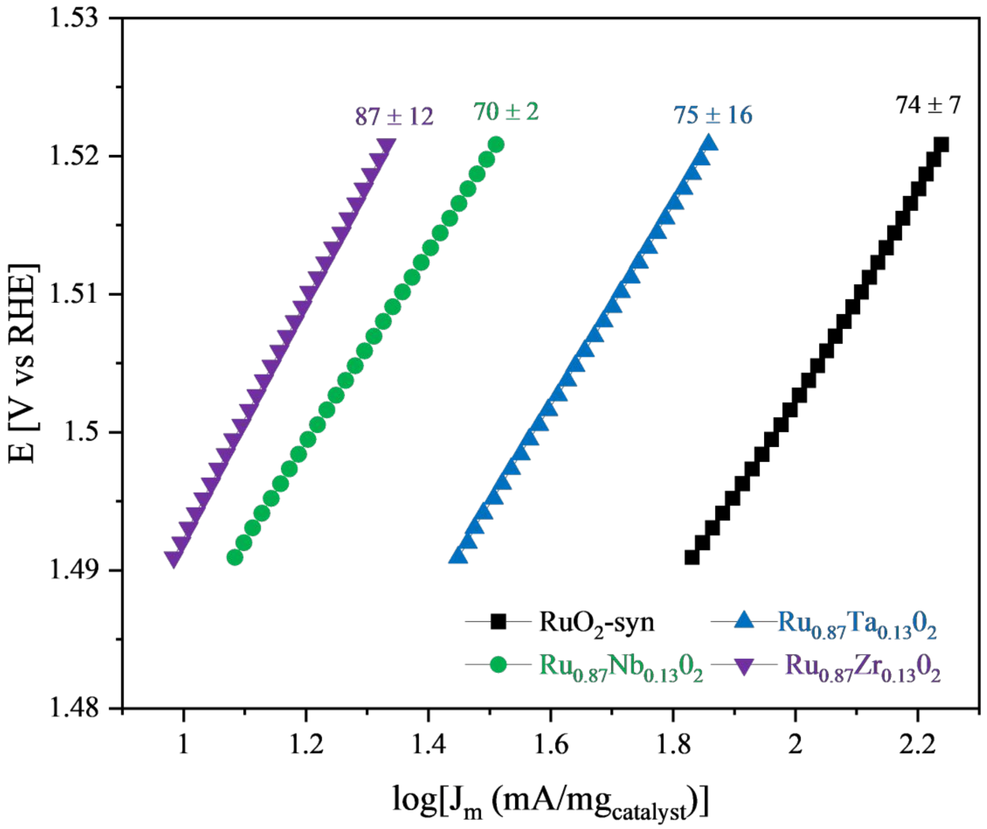

Figure S16. Tafel plots and slopes of RuO_2-syn, $Ru_{0.87}Nb_{0.13}O_2$, $Ru_{0.87}Ta_{0.13}O_2$, and $Ru_{0.87}Zr_{0.13}O_2$ within the 1.49−1.52 V_{RHE} voltage range determined by initial linear sweep voltammetry and normalized to catalyst loading.

Computational Analysis of Dissolution of Zr-5D and -6D from $Ru_{0.75}Zr_{0.25}O_2$-5D and 6D Slabs

In Figure S17a, one can see that the dissolving Zr-5D requires almost no energy intake (~0.09 eV) to break its bond with the subsurface oxygen located below it and reach state II. From it, the energy profile is downhill until reaching stabilization at state III, which is characterized by the dissolving metal adsorbing and partially oxidizing two water molecules, as it breaks one of its Zr-Ot bonds and forms an O_{CUS}-O_{CUS} bond between its O_{CUS} and a neighbor one. From such a state, it requires an additional ~0.78 eV to reach state IV, where the dissolving metal has broken all the surface bonds and keeps attached to the surface only due to the O_{CUS}-O_{CUS} bond described before. To go from state IV to state V the system needs ~1.07 eV that allows the dissolving Zr-5D to break its O_{CUS} bond, leaving an O_2* adsorbed on the surface and a ZrO_3H_4 dissolving species with two HO* and one H_2O* adsorbed to it. Finally, with an energy intake of ~0.36 eV the dissolving species fully incorporates into the aqueous media while solvated by two additional water molecules.

Finally, Zr-6D dissolution was also evaluated, as summarized in Figure S17b. As can be seen in the free energy profile, triggering Zr-6D dissolution is barrierless as the dissolving metal, breaking the main subsurface bonds and starting to lose the surface ones, exhibits no activation energy (state II). Moreover, ~0.44 eV are needed to break all surface bonds with the system reaching state III, where the dissolving metal has bonded with two O_{CUS} and has adsorbed two free water molecules and started to oxidize them. In comparison with Ru dissolution, dissolving Zr doesn't attract surface oxygens to it, so it doesn't leave behind new metal active sites. Additional ~0.39 eV allows the dissolving compound to reorient with respect to the surface and the electrolyte, breaking the first O_{CUS} bond formed in state III and keeping the bond to the second O_{CUS} on the surface in state IV. Regarding the incorporation into the aqueous media, an intake of ~0.96 eV is needed for the system to reach stable state V, where it has partially oxidized the initially adsorbed water molecules and has adsorbed a fourth one, forming a ZrO_5H_7 dissolved species.

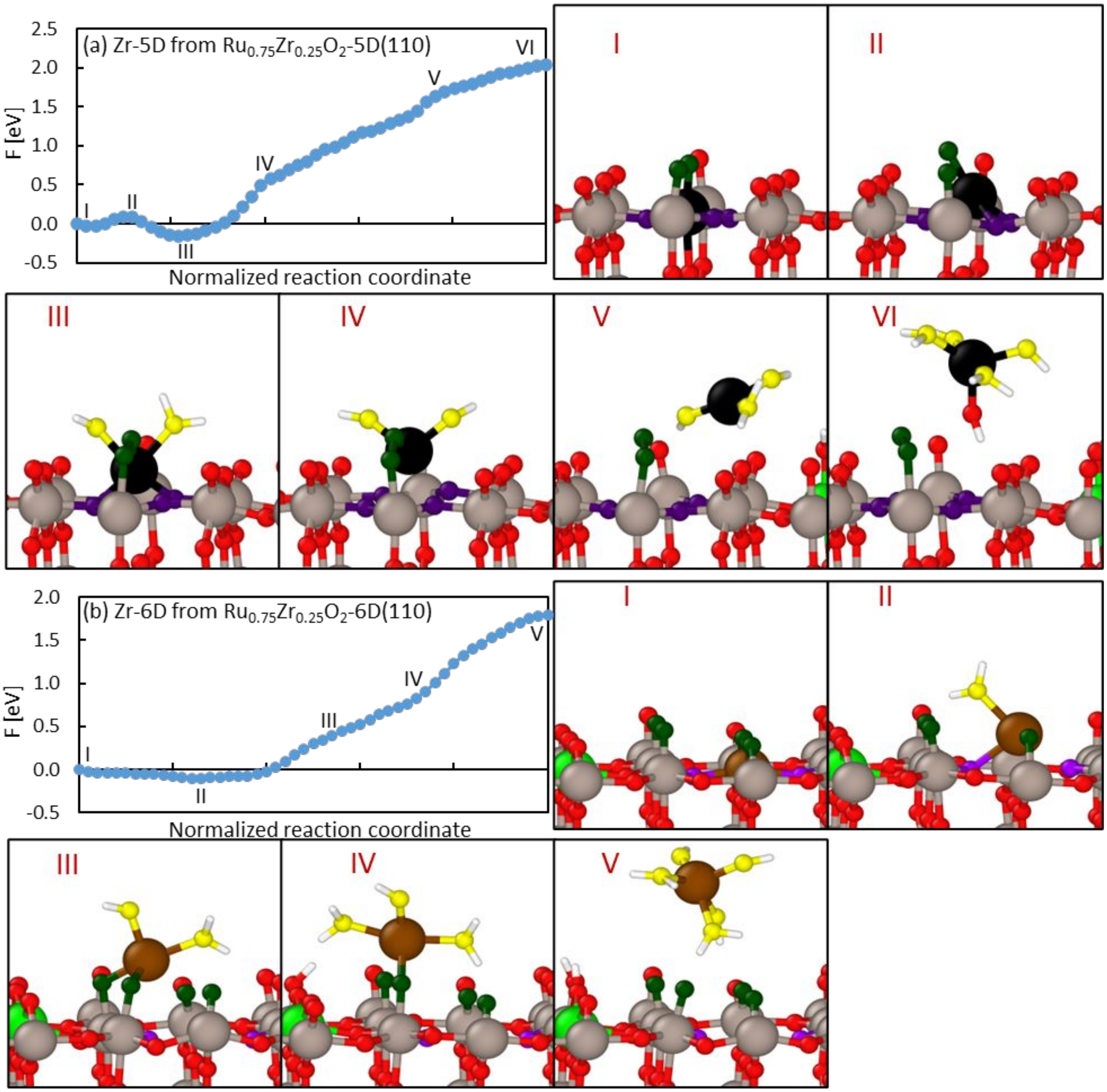

Figure S17. Free energy profile of zirconium (a) penta- (5D) and (b) hexa- (6D) coordinated dissolution from the $Ru_{0.75}Zr_{0.25}O_2$-5D and 6D (110) surfaces, with the intermediate events along the dissolution path labeled from I (initial state) to VI (Fully dissolved species). See main text for explanation of the intermediate events. Color code: Ru – silver, Zr – light green, dissolving Zr – black, O – red, H – white, Ot – purple, O_{CUS} and O_B – dark green, O from free water molecule – yellow.

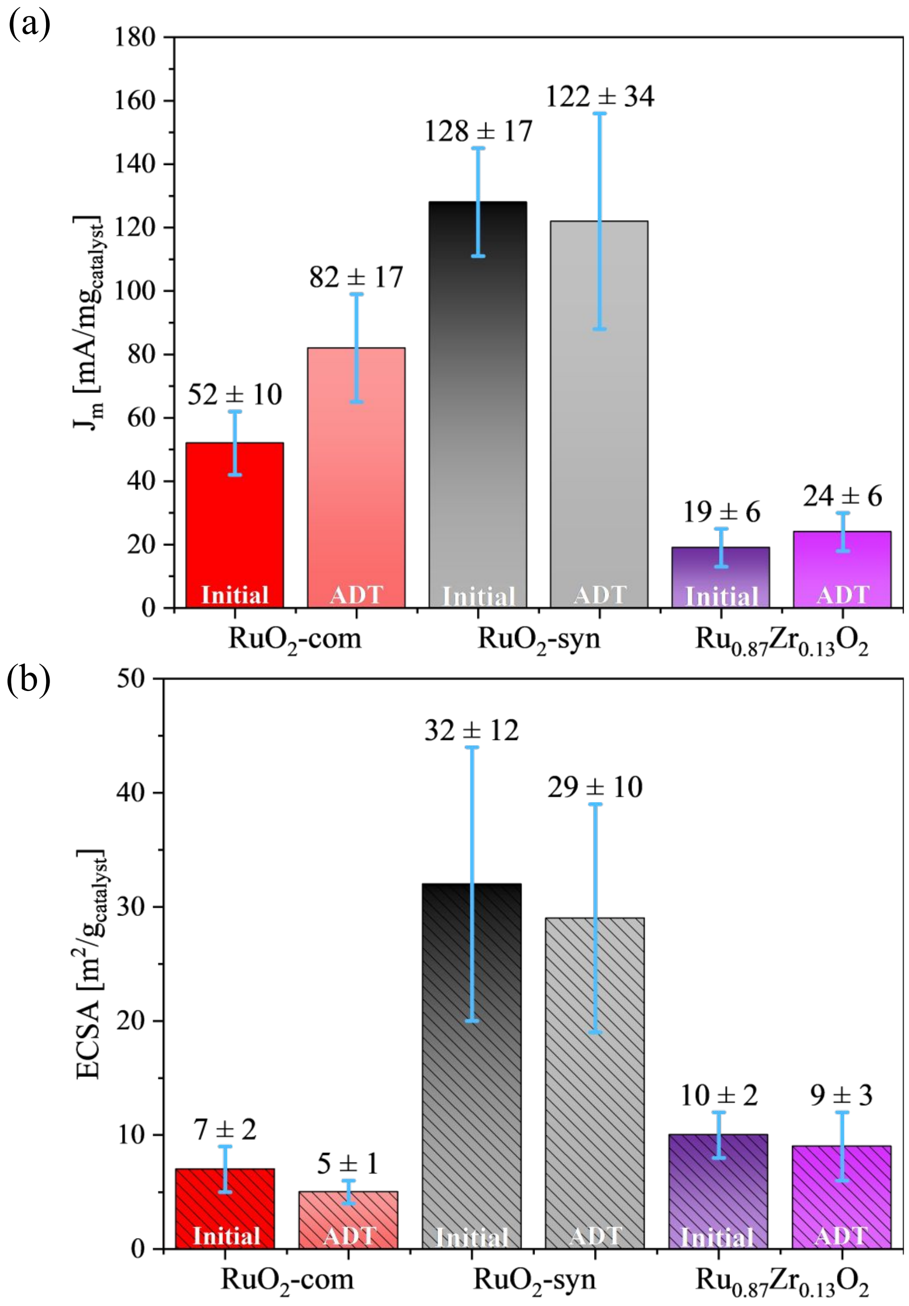

Figure S18. Comparison of rotating disk electrode (RDE) results initial and after accelerated durability test (ADT) for RuO_2-com, RuO_2-syn, and $Ru_{0.87}Zr_{0.13}O_2$ tested in Ar-purged 0.1 M $HClO_4$ under rotation at 2500 rpm: (a) OER mass activities at 1.51 V_{RHE} from linear sweep voltammetry, and (b) electrochemical surface area (ECSA).

Table S9. Ruthenium (Ru), and zirconium (Zr) concentrations determined from ICP-MS and wt% dissolved within the electrolyte after accelerated durability testing of RuO_2-com, RuO_2-syn and $Ru_{0.87}Zr_{0.13}O_2$.

Material	Ru Dissolution (ppb)	Zr Dissolution (ppb)	Ru Dissolution (wt%)	Zr Dissolution (wt%)
RuO_2-com	0.66 ± 0.01	-	1.25 ± 0.02	-
RuO_2-syn	0.51 ± 0.16	-	0.93 ± 0.30	-
$Ru_{0.87}Zr_{0.13}O_2$	0.36 ± 0.15	0.21 ± 0.19	0.84 ± 0.34	4.32 ± 3.89

References

1. Morgan, D. J., Resolving ruthenium: XPS studies of common ruthenium materials. *Surface and Interface Analysis* **2015,** *47* (11), 1072-1079.
2. Lin, Y.; Tian, Z.; Zhang, L.; Ma, J.; Jiang, Z.; Deibert, B. J.; Ge, R.; Chen, L., Chromium-ruthenium oxide solid solution electrocatalyst for highly efficient oxygen evolution reaction in acidic media. *Nat Commun* **2019,** *10* (1), 162-162.
3. Rochefort, D.; Dabo, P.; Guay, D.; Sherwood, P. M. A., XPS investigations of thermally prepared RuO2 electrodes in reductive conditions. *Electrochimica Acta* **2003,** *48* (28), 4245-4252.
4. Kim, K.; Kim, M.-S.; Cha, P.-R.; Kang, S. H.; Kim, J.-H., Structural Modification of Self-Organized Nanoporous Niobium Oxide via Hydrogen Treatment. *Chemistry of Materials* **2016,** *28* (5), 1453-1461.
5. Skrodczky, K.; Antunes, M. M.; Han, X.; Santangelo, S.; Scholz, G.; Valente, A. A.; Pinna, N.; Russo, P. A., Niobium pentoxide nanomaterials with distorted structures as efficient acid catalysts. *Communications Chemistry* **2019,** *2* (1), 129.
6. Weibin, Z.; Weidong, W.; Xueming, W.; Xinlu, C.; Dawei, Y.; Changle, S.; Liping, P.; Yuying, W.; Li, B., The investigation of NbO_2 and Nb_2O_5 electronic structure by XPS, UPS and first principles methods. *Surface and Interface Analysis* **2013,** *45* (8), 1206-1210.
7. Gonçalves, R. V.; Wojcieszak, R.; Uberman, P. M.; Teixeira, S. R.; Rossi, L. M., Insights into the active surface species formed on Ta_2O_5 nanotubes in the catalytic oxidation of CO. *Physical Chemistry Chemical Physics* **2014,** *16* (12), 5755-5762.
8. Wu, B.; Wang, H.; Wu, C.; Du, F.; Chu, J.; Wang, X.; Xiong, S., Ru-doped Ta_2O_5 supported Pt nanoparticles: an efficient electrocatalyst for methanol oxidation. *Ionics* **2021,** *27* (10), 4361-4369.
9. Bumajdad, A.; Nazeer, A. A.; Al Sagheer, F.; Nahar, S.; Zaki, M. I., Controlled Synthesis of ZrO_2 Nanoparticles with Tailored Size, Morphology and Crystal Phases via Organic/Inorganic Hybrid Films. *Scientific Reports* **2018,** *8* (1), 3695.
